# Mercury mobilization and export from the Greenland Ice Sheet using an ice-to-ocean approach

**DOI:** 10.1038/s43247-026-03818-z

**Published:** 2026-07-16

**Authors:** Amina N. Youssef, Sarah E. Janssen, Carl Lamborg, Michael T. Tate, Marissa Despins, Eva L. Doting, Brett A. Poulin, David McCabe, Leah M. Hopf, Emma K. Ferrer, Abe Doroshow, Guillaume Lamarche-Gagnon, Ian Delaney, Marjolein Gevers, Jade Hatton, Iris Kubler-Dudgeon, Jack G. Murphy, Amina T. Schartup, Marek Stibal, Johannes West, Jon R. Hawkings

**Affiliations:** 1 University of Pennsylvania, Philadelphia, PA USA; 2 United States Geological Survey, Madison, WI USA; 3 University of California, Santa Cruz, CA USA; 4 Norwegian Institute for Nature Research (NINA), Tromsø, Norway; 5 University of California, Davis, CA USA; 6 iC3, UiT – The Arctic University of Norway, Tromsø, Norway; 7 University of Lausanne, Lausanne, Switzerland; 8 UK Centre for Ecology & Hydrology, Bangor, Gwynedd, UK; 9 Charles University, Prague, Czechia; 10 Scripps Institution of Oceanography – University of California San Diego, San Diego, CA USA

**Keywords:** Element cycles, Cryospheric science, Element cycles, Element cycles

## Abstract

The Greenland Ice Sheet (GrIS) is a poorly constrained source of mercury (Hg) to Arctic ecosystems. We measured Hg concentrations and stable isotopes along an ice-to-ocean continuum to identify controls on GrIS Hg export. Early-season permafrost melt and rainfall produced high filtered total mercury (fTHg, ~17 pM) and monomethylmercury (MMHg, ~2 pM). As subglacial drainage evolved, particulate Hg doubled (from ~8 to 17 pM) and MMHg production remained elevated, indicating Hg mobilization from subglacial environments. Shifts in Hg stable isotope ratios and Δ^199^Hg mass balance show supraglacial sources contribute 20–48% of exported Hg, suggesting subglacial inputs dominate the seasonal Hg flux. Fjord waters were enriched in fTHg ( ~ 10 pM) and MMHg ( ~ 2 pM) relative to rivers, consistent with particulate Hg transformations and terrestrial Hg inputs. The estimated GrIS Hg yield ( ~ 23 mmol km^−2^ yr^−1^) is similar to that of Arctic rivers and will likely increase with climate-driven mass loss.

## Abstract (web summary)

Subglacial inputs dominate the export of mercury (Hg) from the Greenland Ice Sheet, and the estimated Hg yield is similar to that of Arctic rivers, according to analysis of Hg concentrations and stable isotopes from the southwestern Greenland Ice Sheet margin during the 2023 ablation season

## Introduction

Mercury (Hg) is a global contaminant that poses a threat to both ecosystems and human health. Anthropogenic activities, such as industrial emissions^1,2^, have increased atmospheric Hg concentrations (predominantly gaseous elemental mercury, Hg(0)) and altered its environmental distribution^3^, including the transport and deposition of human-derived Hg to Arctic regions^4^. Once in the atmosphere, Hg(0) undergoes oxidation and is deposited to the surface mostly as inorganic Hg(II), linking atmospheric transport to subsequent environmental cycling^5^. In addition to anthropogenic Hg, natural and climatically sensitive Hg reservoirs, including permafrost^6^ and glaciers^7^, are important Hg sources but remain poorly quantified in the Arctic Hg budget^8^. The Arctic is warming nearly four times faster than the global average, and therefore understanding the role of these climatically sensitive reservoirs in the Hg cycle is crucial^9^.

Meltwater from the Greenland Ice Sheet (GrIS) is important in a variety of biogeochemical cycles, particularly in the export of trace elements to coastal environments^10,11^. However, the contribution of the GrIS to the Arctic Hg cycle remains highly uncertain due to large differences in estimated Hg yields (1440–2310 mmol km^−2^ yr^−1^ versus 2.7–5.2 mmol km^−2^ yr^−1^)^12,13^. These previous studies are limited by their reliance on nontraditional Hg sampling methods, irregular sampling regimes, and/or short time series that fail to capture the full complexity of rapidly evolving glacial systems and major hydrological shifts in ice sheet discharge. High physical erosion rates beneath the GrIS result from ice sliding over bedrock, and subglacial hydrological networks flush finely ground, reactive particles from the ice-rock interface^14,15^. During peak ice melt, glacial hydrological systems efficiently transport surface and subglacial meltwater and eroded subglacial bedrock to the ice margin^16,17^. These glacial processes mobilize and transport Hg from both atmospherically deposited and geogenic sources^18,19^. Sediment-rich freshwater mobilized from the GrIS transits through fjords^20^, undergoing biotic and abiotic transformations (e.g., nutrient cycling, redox-driven changes in trace metal speciation, aggregation or desorption of particulates, and Hg methylation)^21–23^. Given that 97% of the Arctic glacial Hg pool is estimated to be stored within the GrIS^7^, it is critical to understand Hg sources, transformations, and fluxes from this reservoir.

**Fig. 1:**
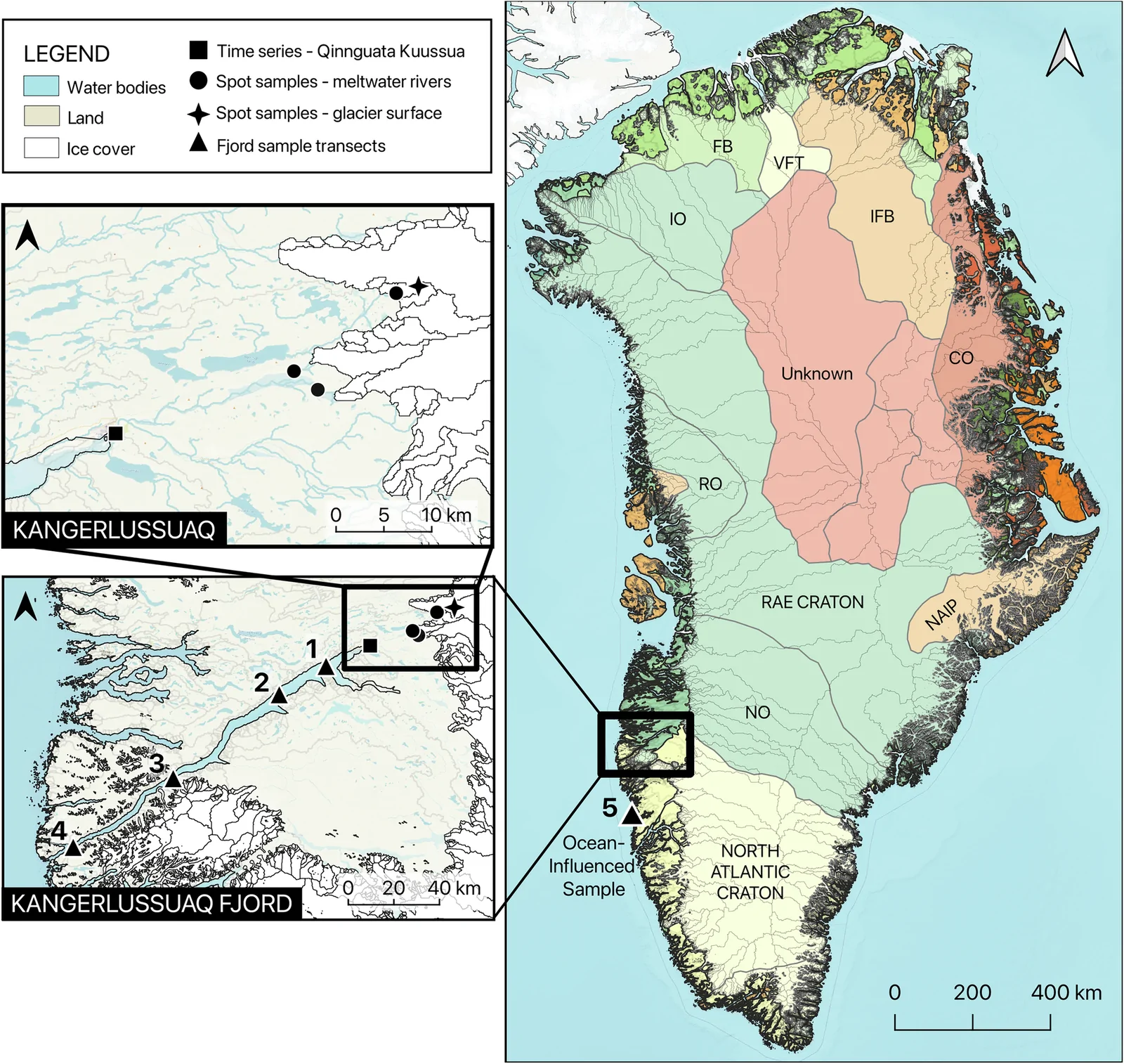
Map of the main sampling locations and subglacial geological provinces beneath the Greenland Ice Sheet^101^. Legend includes different sampling types and their locations. Fjord sample locations are distinguished by station number. Major geological provinces include the North Atlantic Craton and the Rae Craton. Other geological provinces (bottom to top) include: Nagssugtoqidian Orogen (NO), North Atlantic Igneous Province (NAIP), Rinkian Orogen (RO), Unknown Regions (Unknown), Caledonian Orogen (CO), Independence Fjord Basin (IFB), Victoria Fjord Terrane (VFT), Franklinian Basin (FB), and Inglefield Orogen (IO). Ice basins are shown as thin, black lines, overlaying geological provinces^126^. Figure made using QGreenland^127^ with basemap data from OpenStreetMap contributors^128^.

Here, we characterized Hg sources, transport, and export in meltwaters from the southwestern GrIS margin using Hg concentrations and Hg stable isotope ratios across changing hydrologic regimes. Glacial meltwater was collected daily across the 2023 ablation season from the Qinnguata Kuussua catchment near Kangerlussuaq, Greenland (Fig. 1; also known as the Watson River), with additional synoptic samples collected from proglacial streams and subglacial meltwater outlets. Hg concentrations and chemical species were measured in the receiving fjord (Fig. 1; Kangerlussuaq Fjord, also known as Søndre Strømfjord), representing an ice-to-ocean assessment of Hg sources, transformations, and sinks. Atmospheric Hg concentrations were measured to account for regional atmospheric Hg deposition. To test the hypothesis that rock weathering and subglacial meltwater flushing from beneath the GrIS mobilize Hg to receiving fjords, we used Hg stable isotope ratios to decipher geogenic and atmospheric source contributions in glacial meltwater, trace photochemical degradation, and provide context to seasonal variations in regional Hg export. The findings are critical for understanding contemporary and future Hg contributions from climatically sensitive Hg reservoirs, identifying future monitoring needs within Arctic environments, and informing modeling efforts to constrain global Hg budgets.

## Results and discussion

### Seasonal hydrology

**Fig. 2:**
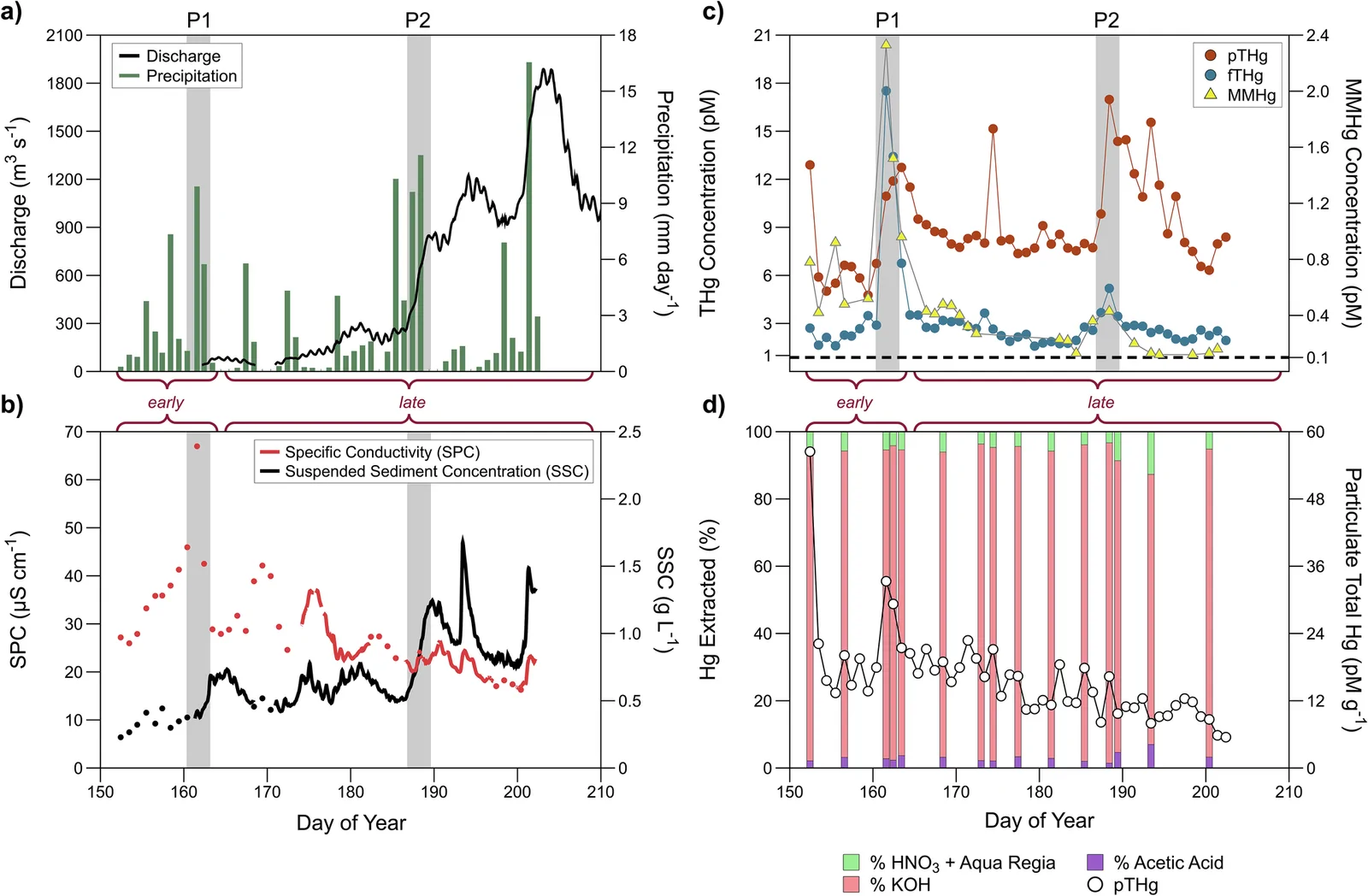
Time series of hydrological and geochemical data in the Qinnguata Kuussua in 2023. **a** discharge (black) and precipitation (green vertical bars); **b** specific conductivity (SPC, red) and suspended sediment concentration (SSC, black); **c** mercury (Hg) species and fractions (particulate total Hg (pTHg, >0.45 μm, red), filtered total Hg (fTHg, ≤0.45 μm, blue), and monomethylmercury (MMHg, yellow); and **d** sequential Hg extraction fractions (0.1 M acetic acid, purple; 12 M HNO_3_ + aqua regia, green; KOH, orange). Gray shaded regions identify large precipitation events (P1, P2) and early season (days 152–164) and late season (days 165–202) periods are denoted. In **c**, detection limits are identified (~1.0 pM for pTHg and fTHg, 0.10 pM for MMHg). In panel d), the daily particulate total Hg concentrations per gram of sediment (pM g^−1^) are overlaid.

High precipitation in 2023 resulted from a series of extreme weather events, including multiple atmospheric rivers that reached Western Greenland^24^. These rain events were observed in the field, with July 2023 being one of the wettest and warmest months since 1940^24^ (Fig. 2a). Two exceptionally large rain events (P1 and P2, gray vertical bars; Fig. 2a) occurred alongside rapid changes in river discharge. River discharge doubled after P1, from <40 m^3^ s^−1^ to ~80 m^3^ s^−1^. A peak in atmospheric temperature on day 188 (July 7th) corresponded with P2 and the beginning of the discharge rising limb (Fig. 2a).

Changes in suspended sediment concentration (SSC) closely tracked discharge (Fig. 2b). SSC gradually increased in the early season and was highest during peak melt (after P2; days 188–202), with spikes in SSC also occurring during or immediately after major rainfall events (P1 and P2). In contrast, specific conductivity (SPC) displayed a gradual seasonal decline (Fig. 2b). An early peak in SPC coincided with P1 (day 161), interpreted here to reflect the input of solute-rich terrestrial runoff into the river in response to high precipitation volumes and permafrost flushing. SPC values began to decrease with rising discharge (days 175–202), as dissolved ions became diluted by increased meltwater volumes from glacial surfaces and more efficient and rapid subglacial meltwater drainage^25^. For this study, we define “early” season (days 152–164) when discharge remains low (<100 m^3^ s^−1^), SPC is relatively high, and when the relative contribution of terrestrial runoff is higher. A subsequent “late” season (days 165–202) is defined by the onset of sustained discharge increase (>100 m^3^ s^−1^), and concurrent SPC dilution, indicating the progressive transition to rising supraglacial melt contributions.

### Organic carbon controls on early season Hg concentrations

High rainfall during the early sampling season (P1; Fig. 2a), led to saturated proglacial zones and appeared to enhance Hg mobilization into the Qinnguata Kuussua. Hg concentrations reached 17.5 pM fTHg, 2.3 pM MMHg, and 12.7 pM pTHg during the peak of the storm (Fig. 2c). The observed increases in Hg concentrations during P1 are best explained by hydrological connectivity between saturated proglacial zones and the Qinnguata Kuussua, indicating Hg mobilization is likely attributed to runoff from the active layer of permafrost. Previous research has shown that rising air temperatures increases soil temperatures, leading to deeper migration of the active layer depth and associated permafrost degradation^26^. Changes in the depth of the active layer perturbs soil hydrology, including soil water-retention capacity and hydrological connectivity, which can lead to surface ponding and runoff to downstream aquatic systems^27^.

**Fig. 3:**
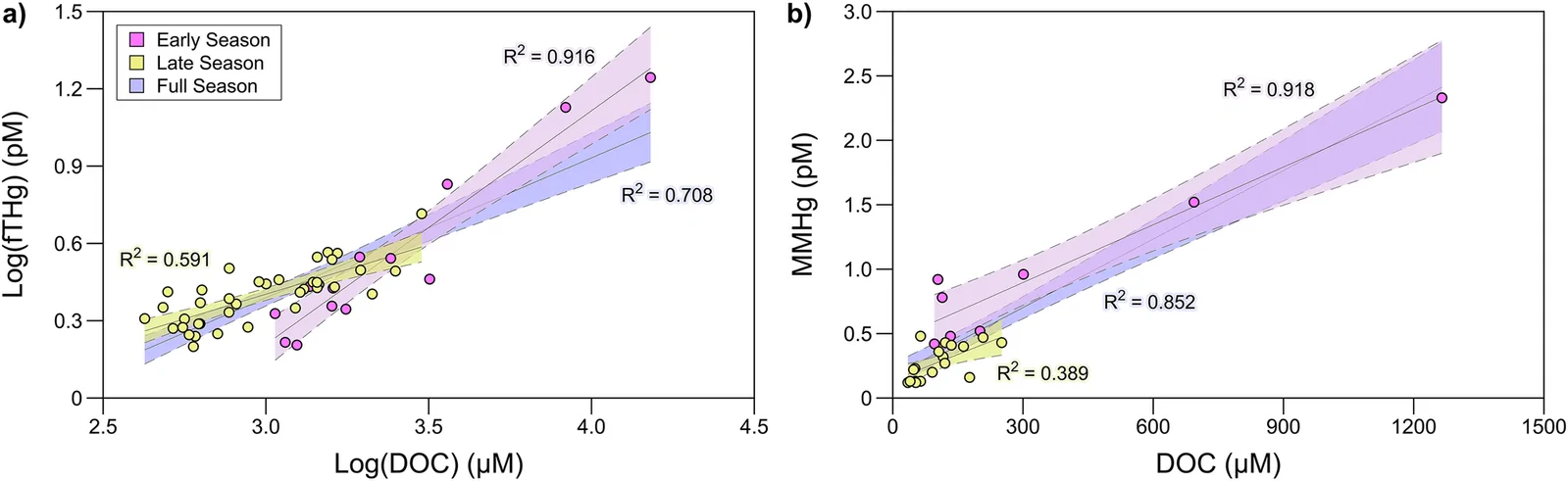
Correlations between filtered Hg fractions and dissolved organic carbon (DOC). **a** filtered total Hg (fTHg) and DOC on the log-log scale, and **b** monomethylmercury (MMHg) and DOC as a linear relationship. Colors distinguish three correlations: (1) early season (days 152 to 164; pink), (2) late season (day 165 to 202; yellow), and full season (152 to 202; purple). Gray lines show the fitted regressions (log–log for fTHg vs DOC and linear for MMHg vs DOC), and shaded regions show 95% confidence intervals of the fitted relationships, indicating the range of values statistically compatible with the data.

A permafrost melt stream sampled during P1 exhibited the highest fTHg concentrations measured across the season (~50 pM; Supplementary Fig. 1) and high DOC concentrations (4739 µM; Table S2) when compared to glacial meltwater (20–122 µM). Strong positive correlations were observed between concentrations of filtered Hg fractions (fTHg, MMHg) and DOC (Fig. 3a, b, R2 ≥ 0.92, *p* < 0.05) in early-season samples, which supports that filter-passing Hg fractions are dissolved species and Hg-DOC coupling drives episodic runoff of terrestrial matter and Hg into the Qinnguata Kuussua. During P1, discharge (~60 m^3^ s^−1^) appears to be primarily driven by precipitation with minimal contribution of glacier melt. Organic matter ligands bind inorganic Hg(II) and promote long-term Hg storage and sequestration in Arctic environments^28–30^, but under warming conditions, permafrost thaw and active layer saturation appears to become an important vector for transport of organic matter and Hg into nearby rivers and streams^6,31^. This is further confirmed by a Principal Component Analysis (PCA; Supplementary Fig. 3), which isolates P1 samples (days 161–162) at the positive extreme of PC1 and indicates that high precipitation, and the subsequent flushing of permafrost active layer, drives strong covariation among dissolved constituents (including fTHg, MMHg, and DOC), a relationship that is weakened in the absence of high precipitation events (Supplementary Fig. 3b).

**Fig. 4:**
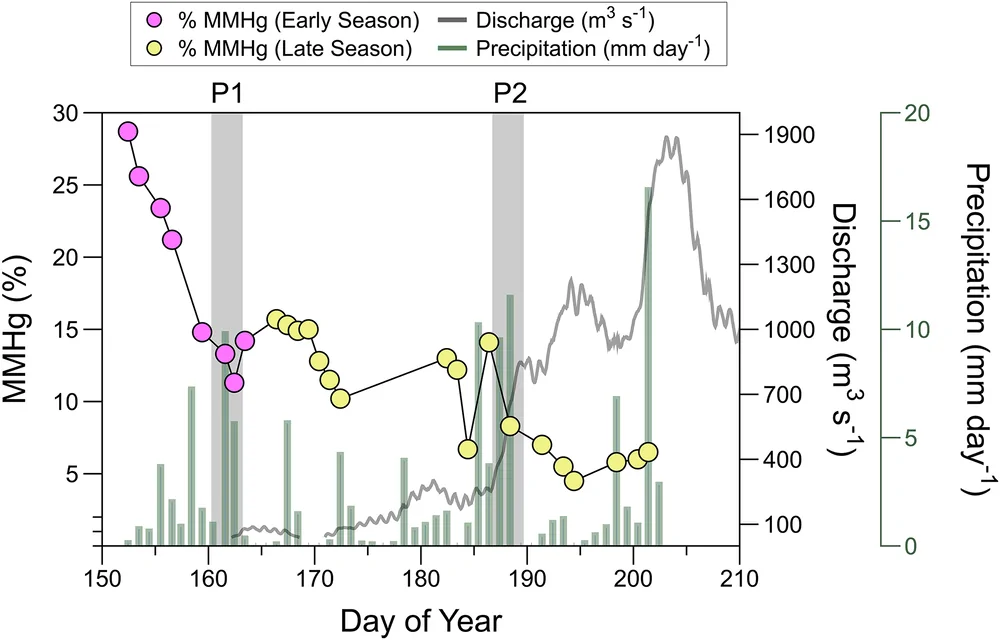
The proportion of MMHg relative to fTHg (% MMHg) in the Qinnguata Kuussua displayed over seasonal discharge (gray line) and seasonal precipitation measurements (green vertical bars). Early season (pink) and late season (yellow) are distinguished. P1 and P2 signify large precipitation events and noticeable changes in river discharge.

Flushing of active layer and permafrost thaw can also release substantial amounts of labile organic carbon, which can serve as an important substrate for microbial methylation of inorganic Hg(II)^32^. The proportion of MMHg relative to fTHg is an indicator of net methylation activity^33^. In the early season and during P1, MMHg in Qinnguata Kuussua accounted for 23.3% of the fTHg pool (termed % MMHg; Fig. 4). This percentage is higher than previously reported for Arctic freshwaters, which have observed % MMHg values between 1 and 10%^34,35^. Higher % MMHg (>20%) points to active methylation processes or enhanced Hg mobilization from zones of methylation, such as organic-rich environments, including active layers of permafrost^36^. MMHg concentrations showed a strong correlation to DOC in the early season (Fig. 3b, R^2^ ≥ 0.92, *p* < 0.05), further confirming that locations of high DOC are also locations of enhanced methylation processes in the Qinnguata Kuussua catchment^37,38^. Early-season conditions of high precipitation coupled to low meltwater river discharge (~30–60 m^3^ s^−1^; Fig. 4) appear to have enhanced MMHg production and/or mobilization from permafrost into the Qinnguata Kuussua. However, we see a decline in % MMHg as discharge increases and at the onset of P1. While ambient concentrations of MMHg were elevated during P1 (2.2 pM) these also corresponded to higher levels of fTHg (17.5 pM), causing a decline in the % MMHg ratio. Overall, we observed a distinctly higher release of MMHg in the early season when compared to the late season, demonstrating the importance of seasonality for MMHg production within this system.

### Ice sheet mobilized Hg

Concentrations for fTHg, MMHg, and pTHg reached maxima largely in response to proglacial inputs during P1, but post-P1, Hg concentrations were also not substantially diluted by increasing meltwater discharge in the late season (Fig. 2c). After P1, fTHg and MMHg concentrations returned to pre-storm levels (late season averages of 2.6 ± 0.7 pM and 0.28 ± 0.13 pM), and average late season pTHg concentrations increased from 6.6 ± 2.4 pM (before P1, days 152 to 160), to 9.4 ± 2.6 pM, suggesting a continuous background Hg source distinct from storm-driven inputs (e.g., P1).

Following P1 (days 165 onward; late season), the Qinnguata Kuussua rapidly shifted from an organic-rich, terrestrially influenced river to one dominated by meltwater input from the GrIS, which is relatively low in organic carbon compared to terrestrial sources^39,40^. DOC concentrations decreased from an average of 285 ± 335 µM (avg ± 1 stdev, *n* = 13) in the early season to an average of 92 ± 51 µM (*n* = 38) in the late season (Table S1). During this transition, the proportion of MMHg relative to fTHg decreased to ~5–10% for the remainder of the season (Fig. 4). This decline suggests that the dominant Hg source in the late season has lower MMHg concentrations than early-season, proglacial-dominated sources. However, the % MMHg of ~5–10% remains high compared to other Arctic environments^34,35^, and late-season discharge (>100 m^3^ s^−1^) does not merely dilute a highly methylated Hg source. While early-season MMHg production appears elevated and correlated to organic-rich runoff, persistently high % MMHg in the late season indicates that there is still a MMHg supply during high glacial discharge, elevated SSC, and comparatively low DOC concentrations. These late-season conditions, together with the rapid transit of the Qinnguata Kuussua, make extensive in-river methylation unlikely.

If the riverine MMHg was only supplied by seasonally persistent proglacial inputs (e.g., permafrost), we would expect a consistent MMHg instantaneous flux across the season. However, simple scaling of the average early-season MMHg instantaneous flux (18 nmol s^−1^) to peak meltwater instantaneous flux (360 nmol s^−1^), indicates that proglacial sources would account for only 5% of the observed peak-melt MMHg load. Using the maximum MMHg concentration observed during P1 (2.2 pM, when we assume the highest proglacial inputs to the river), we estimate proglacial inputs would still only supply ~18% of the peak-melt MMHg load, implying an additional MMHg source to the river. The persistence of MMHg under high meltwater discharge conditions suggests that MMHg production likely occurs in anoxic and previously isolated subglacial environments^41,42^ (driven by microbial methylation of Hg(II)^43^), and is subsequently flushed to the ice margin by supraglacial meltwater^44,45^. In short, high MMHg during peak discharge supports a subglacial source of MMHg to the Qinnguata Kuussua.

Unlike fTHg and filtered MMHg concentrations, pTHg concentrations steadily increased from early season (6.6 ± 2.4 pM) to late season (9.4 ± 2.6 pM). pTHg concentrations across the season mimic SSC and discharge, with days 163, 174, 188, and 193 showing corresponding peaks in pTHg and SSC (Fig. 2b–d). As SSC increased, pTHg per gram of sediment (pM g^−1^) declined (Fig. 2d), suggesting that early-season particulates with higher organic carbon concentrations are more Hg-enriched, and/or that increasing subglacial sediment loads reduced Hg sorption per unit mass^46^. In the late season, including after and during P2, peaks in both SSC and pTHg corresponded with rapid discharge increases (Fig. 2c), suggesting that glacier hydrology and increasing glacial sediment flux enhance pTHg export (even as sediment-normalized concentrations decline), and that exported pTHg is sourced primarily from subglacial physical weathering processes. A qualitative assessment of pTHg lability utilizing sequential particulate Hg extractions demonstrated that most pTHg resides in a labile KOH-extractable phase (87.3–96.7% total extracted Hg; Fig. 2d), consistent with adsorption to reactive mineral surfaces and secondary weathering products generated at the ice-bed interface^47,48^ (Supplementary Text 5).

The Qinnguata Kuussua has two upstream tributaries, including the Akuliarusiarsuup Kuua (upstream tributary fed by Russell and Leverett glacier) and an additional tributary that converges with the Akuliarusiarsuup Kuua (67.019°N, 50.5656°W; named Qinnguata Kuussua). Synoptic samples from the Akuliarusiarsuup Kuua tributary, including Leverett and Russell subglacial outflows (Supplementary Fig. 1), reinforce the interpretation that pTHg in the Qinnguata Kuussua is of subglacial origin. Both glaciers had high pTHg concentrations (up to 22.2 pM), comparable to those observed in the late-season time series. The converging tributary (upstream Qinnguata Kuussua; Supplementary Fig. 1) also had similar Hg concentrations, averaging 9.2 ± 2.0 pM (*n* = 4) pTHg and 2.6 ± 0.3 pM (*n* = 4) fTHg, suggesting a consistent Hg source between neighboring glacial catchments and the downstream time series sampling site.

To distinguish between ice surface (supraglacial) and bed (subglacial) sources of Hg, supraglacial samples collected from the GrIS ice margin surface were compared to subglacial samples. Supraglacial waters had universally low Hg concentrations, (e.g., pTHg of 0.6 ± 0.1 pM, *n* = 3, fTHg of 2.2 ± 0.5 pM, *n* = 3; Supplementary Fig. 1). This aligns with previous studies, which show that supraglacial streams carry low metal loads compared to subglacial outflows, as erosion and bedrock weathering release metals^11^.

### Glacier melt and hydrology control the Hg flux

**Fig. 5:**
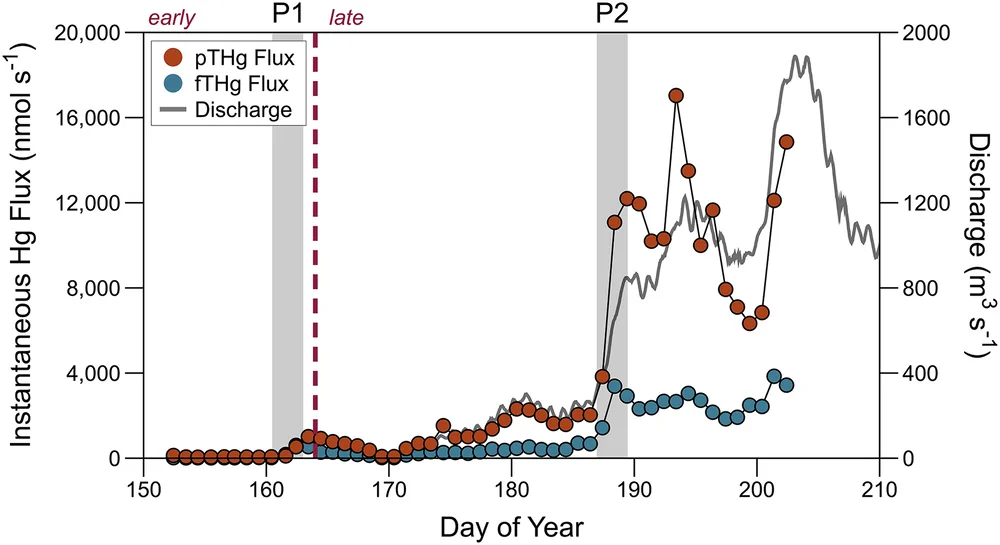
Instantaneous pTHg (red) and fTHg (blue) flux in the Qinnguata Kuussua. Instantaneous Hg flux (nmol s^−1^) was calculated by multiplying the discharge by the pTHg and fTHg concentrations. Discharge is shown as a gray line. A red dashed line separates early-season conditions and late-season conditions, when glacial meltwater substantially increased.

Subglacial drainage evolution is intricately connected to supraglacial meltwater production^44,45,49^. Subglacial channels develop into efficient conduits that rapidly transport meltwater and eroded subglacial bedrock (including overridden paleosols) to the ice margin in response to increased supraglacial meltwater input^16,17,50^. Previous research has demonstrated that evolving subglacial hydrology and subsequent glacial suspended sediment export appear to mobilize increasingly large amounts of Hg compared to non-glacierized watersheds from the same region^51^. Suspended sediments produced by glacial abrasion may be an important geogenic source of Hg to downstream environments^19^. Here, evolving subglacial hydrological systems and corresponding water sources impacted Hg mobilization in the river, as observed by the rising instantaneous Hg flux (Fig. 5). For example, the instantaneous pTHg flux closely tracked discharge and increased rapidly with meltwater production and the expansion of subglacial drainage on day 188 (P2; ~2000 on day 185 to ~14,000 nmol s^−1^ on day 188). This suggests that melt-driven hydrological transitions are likely a key control on Hg export in glacierized catchments.

Beyond physical erosion, extended meltwater residence times in large ice sheet catchments may also enhance subglacial biogeochemical weathering^52,53^, and increase filterable Hg loads observed at peak ablation. In high-melt years, the snowline tends to recede to higher elevations, which may increase meltwater transport capacity to the glacial bed further inland^16^. This process can flush out long-residence-time subglacial waters (stored for a year or longer), enriched in solutes from prolonged rock-water interaction^25,54^. These remote regions are proposed to be regions of hypoxia or anoxia due to limited atmospheric exchange and oxygen consumption by microbial activity (e.g., respiration)^55–57^. Under such conditions, dissolved Hg, particularly MMHg, may accumulate due to anaerobic methylation of Hg(II), which occurs across a variety of microbial groups^41,42^. This is evidenced by late-season fTHg and MMHg peaks around day 190 (P2; a tripling of fTHg and MMHg concentrations; Fig. 2c), and an instantaneous fTHg flux of ~3384 nmol s^−1^ (Fig. 5), which coincide with subglacial hydrological expansion and a rapid spike in SSC and SPC (Fig. 2b). These observations likely reflect the flushing of dissolved Hg from previously isolated and potentially anoxic subglacial regions. A subglacial source of dissolved Hg is further substantiated by low supraglacial fTHg (~2.2 ± 0.5 pM fTHg; Supplementary Fig. 1), and undetectable supraglacial MMHg (<0.1 pM). Changing glacial hydrology across the melt season therefore appears to play a role in subglacial Hg fluxes, speciation, and subsequent downstream export, including fluxes of the dissolved Hg fraction.

### Examining Hg provenance using stable isotopes

**Fig. 6:**
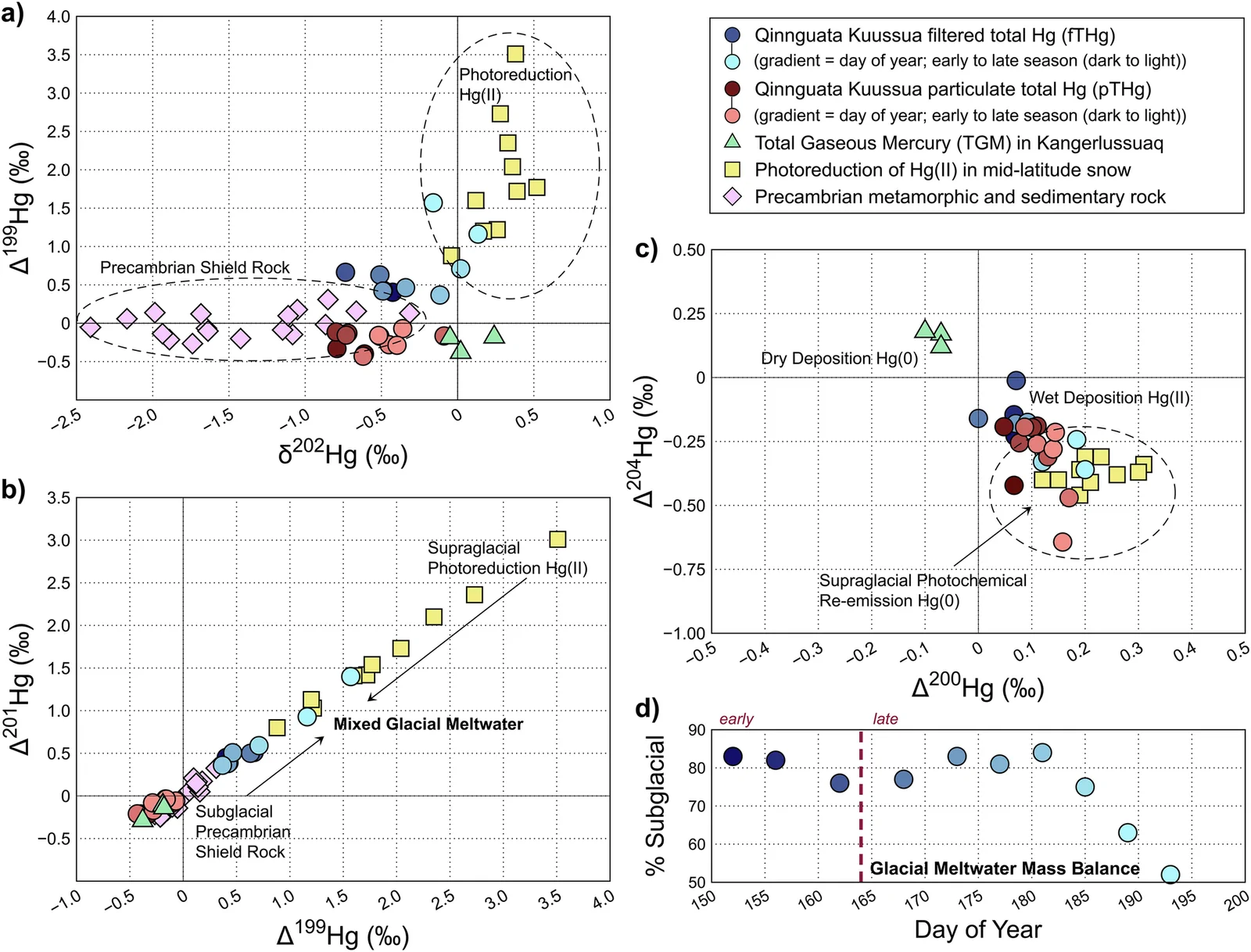
Hg isotope ratios. **a** δ^202^Hg (‰), mass-dependent fractionation (MDF) vs. Δ^199^Hg (‰), mass-independent fractionation (MIF). **b** Δ^199^Hg (‰) vs Δ^201^Hg (‰), MIF for odd isotopes. **c** Δ^200^Hg (‰) vs Δ^204^Hg (‰), MIF for even isotopes. **d** Δ^199^Hg isotope mass balance used to show % Subglacial contribution of Hg across Day of Year. Color gradients for Qinnguata Kuussua samples (fTHg and pTHg) correspond with day of year (dark = early season, light = late season). Legend includes all sample types, including endmembers plotted from previous literature: Precambrian rock, pink^66^, and Hg(II) in mid-latitude snow, yellow^73^. Standard errors for isotope measurements are smaller than the size of each point.

Hg stable isotopes were used to distinguish filtered and particulate Hg sources in the Qinnguata Kuussua. Hg isotopes undergo mass-dependent fractionation (MDF) and mass-independent fractionation (MIF)^58,59^, offering additional insights into Hg sources, riverine transport^60,61^, and transformations (i.e., (de)methylation and sorption)^62,63^, which are useful for distinguishing sources between early and late season. Large changes in δ^202^Hg of suspended particulate Hg and filtered Hg were observed across the sampling period (Fig. 6a), which we attribute to evolving glacial hydrology and associated mineral weathering, as previously seen in samples of geogenic and ice melt origin^64,65^.

The filtered δ^202^Hg increased over time, with δ^202^Hg in early and mid-June ranging from −0.74‰ to −0.43‰ and increasing to 0.13‰ by the end of July (Fig. 6a; the shift in δ^202^Hg exceeded the analytical precision of ± 0.08‰). This change can be attributed to increased input of photochemically reduced meltwater as observed in the late-season samples (Fig. 6a). Additionally, the consistent δ^202^Hg increase observed in the early season suggests a distinct change of δ^202^Hg source, independent of changes in Δ^199^Hg. We attribute the increase in δ^202^Hg across sampling days 150–180 to geogenic rock weathering (Fig. 6a) because these values fall within the δ^202^Hg range of Precambrian rock^66^, and prolonged water-rock interactions during geogenic weathering leads to mineral dissolution and secondary weathering product formation, a process which has been shown to result in isotopically heavy δ^202^Hg in the dissolved phase^67^. The increasing filtered δ^202^Hg with rising glacial discharge likely indicates that mineral weathering liberates subglacial Hg into the dissolved phase, particularly in the late July samples (Fig. 6a). Higher δ^202^Hg in late July (0.02‰ on day 185 and 0.13‰ on day 189) indicates a shift in the Hg source that is more influenced by photochemical reactions, as discussed below.

Values of δ^202^Hg for particulates collected in the Qinnguata Kuussua overlap with the previously published range associated with Precambrian metamorphic and sedimentary bedrock^66^. Across the season, particulate isotopic values also showed a general positive δ^202^Hg shift from early (−0.80‰) to late season (−0.09‰) (Fig. 6a). Hg stable isotopic measurements of particulate matter from glacial sources are limited, with results to-date primarily assessed in non-glaciated watersheds. Previously published particulate values from the Mackenzie River Basin (a large Arctic river, Canada) and Snake River Basin (a temperate, but geologically similar basin, USA) exhibited a broad range of particulate δ^202^Hg (−2.12‰ to −1.55‰ and −1.76‰ to −0.75‰, respectively), attributed to terrestrial and geogenic sources^60,61^. Based on these two previous studies, it is possible that early-season particulate δ^202^Hg is more associated with organic matter (e.g., permafrost or soil), resulting in slightly more negative δ^202^Hg values, similar to the terrestrial values observed in Campeau et al., (2022) and forested basins in Baldwin et al., (2024). Later in the season, particulate δ^202^Hg values become more positive and resemble particulate δ^202^Hg previously measured in geologically influenced river basins^60^. This modest shift may relate to increased particle input from subglacial geogenic rock weathering, supported by previous research that highlights higher δ^202^Hg values to often be associated with low organic matter content matrices (e.g., geogenic materials)^68,69^.

### Photochemical transformations in supraglacial meltwater

MIF of odd isotopes (Δ^199^Hg and Δ^201^Hg) are most often attributed to photochemical transformations^70^. fTHg showed increasingly positive Δ^199^Hg (Fig. 6a, b), from 0.40‰ (day 152) to 1.57‰ (day 202) over the sampling period, with the most prominent shift occurring from day 185-195. These values are similar to those observed in Campeau et al. (0.09‰ to 1.27‰) and Baldwin et al. (0.29‰ to 1.13‰) indicating photochemical reduction within the dissolved phase. Typically, positive odd-MIF is attributed to aqueous in-situ photochemical reduction within surface waters, but has also been linked to wet deposition of Hg(II)^71^. In a glacial system, both processes, including enhanced photochemical degradation and atmospheric inputs, may be contributing to increasing odd-MIF values across the season. The GrIS surface is exposed to extremely high levels of solar radiation in the summer, under almost 24-hour daylight. Recently deposited atmospheric Hg(II) in the supraglacial environment can therefore readily undergo photochemical reduction (Hg(II) → Hg(0)) and re-emission back into the atmosphere^72^. This process produces large, positive Δ^199^Hg fractionations, with previous literature reporting up to 3.50‰ in mid-latitude snow collected in Michigan, USA^73^ and up to 4.06‰ in glacial surface ice samples collected in the glacierized Tibetan Plateau^65^. As surface meltwater production intensifies over the season, a larger fraction of meltwater is sourced from the illuminated ice surface rather than basal melt or stored waters^74,75^. This is likely to lead to increasingly more positive Δ^199^Hg values (Fig. 6b), as a large proportion of deposited Hg could be rapidly re-emitted to the atmosphere^65^. Photochemical reactions have been found to simultaneously affect both MIF and MDF signatures^76,77^, indicating the late-season photochemical reduction of Hg(II) is likely responsible for the positive shift in filtered δ^202^Hg values across the ablation season. This relationship is less evident in pTHg, as the particulates have relatively low Δ^199^Hg values and showed more limited seasonal change in Δ^199^Hg (−0.43‰ to −0.07‰) and Δ^201^Hg (−0.22‰ to 0.04‰). Previous research has found pTHg to have small differences in odd-MIF^60,61^, and rock typically shows little to no MIF and slightly negative MDF signatures^78^. This supports the interpretation that pTHg originates from bedrock weathering.

Given the intense ultraviolet (UV) irradiation experienced in supraglacial environments, we would anticipate supraglacial GrIS meltwater to exhibit pronounced positive odd-MIF signatures (i.e., >3.50‰ Δ^199^Hg)^73^, although never measured before. Despite this, our filtered Δ^199^Hg samples showed lower odd-MIF than those reported from glacial surface ice samples in the Tibetan plateau^65^ as well as mid-latitude snow^73^ (Fig. 6b). A lower than expected odd-MIF suggests that the source of fTHg in the Qinnguata Kuussua is a mix of surface and subsurface glacially-derived Hg similar to those observed in the Urumqi Glacier in China and ice cores from the Canadian Arctic^79,80^. We propose that at peak melt, supraglacial meltwaters with predicted positive Δ^199^Hg and Δ^201^Hg will first drain to the subglacial bed, where they mix with dark, no-MIF waters before exiting at the glacial terminus, thereby reducing the odd-MIF signal (e.g., average Δ^199^Hg for days 150–182 was 0.05‰). Positive odd-MIF therefore likely serves as an effective marker to quantify surface melt relative to subglacial sources. Using a Δ^199^Hg isotope mass balance (Eq. 1), we estimated the percentage of subglacial meltwater Hg contribution across the season using two endmembers (% Subglacial fTHg, Fig. 6d):

1 $$\%\,\, {Subglacial}\,{fTHg}=\left(\frac{\left({{\rm\Delta }}^{199}{{\mathrm{Hg}}}-{{\mathrm{fTHg}}}\right)-\left({{\rm\Delta }}^{199}{{\mathrm{Hg}}}-{{\mathrm{snow}}}\right)}{\left({{\rm\Delta }}^{199}{{\mathrm{Hg}}}-{{\mathrm{sub}}}\right)-\left({{\rm\Delta }}^{199}{{\mathrm{Hg}}}-{{\mathrm{snow}}}\right)}\right)\times100$$

where ${\Delta }^{199}{{{\rm{Hg}}}}-{{{\rm{fTHg}}}}$ is the measured value of each filtered water sample (this study; Δ^199^Hg-fTHg), ${\Delta }^{199}{{{\rm{Hg}}}}-{{{\rm{snow}}}}$ is an endmember from mid-latitude snow, used as a proxy for supraglacial meltwaters (3.50‰ ${\Delta }^{199}{{{\rm{Hg}}}}$)^73^, and ${\Delta }^{199}{{{\rm{Hg}}}}-{{{\rm{sub}}}}$ is the average ${\Delta }^{199}{{{\rm{Hg}}}}$ observed across the season for particulate matter (0.23‰; this study), used as a proxy for bedrock weathering in subglacial environments (a more detailed description of endmember selection can be found in the methods). A higher percentage indicates little to no photochemically altered ${\Delta }^{199}{{{\rm{Hg}}}}$ waters are present in the mixture, and a higher contribution of subglacial derived Hg, whereas a lower percentage indicates the reverse.

We observed a seasonal change in subglacial contributions (from 83% to 52% of fTHg), driven by increasing supraglacial meltwater contributions after day 180 (e.g., beginning of discharge rising limb; Fig. 6d). In the early season, we estimate subglacial and proglacial (e.g., permafrost) Hg contributions to the river comprised ~80% of the fTHg pool, suggesting that the supraglacial or snowmelt contribution was <20%. At peak discharge, supraglacial sources contribute as much as 48% of the fTHg pool, showing that roughly half of the fTHg likely originates from subglacial sources. Thus, we conclude that subglacial Hg is a dominant fTHg source to the river, surpassing seasonal inputs from atmospheric deposition and supraglacial meltwater. Notably, this mixing model provides a conservative estimate of the subglacial Hg fraction because the supraglacial endmember for photochemical reduction (3.50‰)^73^ is lower than what we would predict for the GrIS surface. To better constrain subglacial and supraglacial sources from the GrIS, future studies should consider the collection of additional endmembers including snow, ice, permafrost, and geogenic material.

### Atmospheric sources of Hg to Qinnguata Kuussua

The ${\Delta }^{200}{{{\rm{Hg}}}}$ and ${\Delta }^{204}{{{\rm{Hg}}}}$ values are understood to arise from photochemical reactions in the upper troposphere and stratosphere^81,82^, providing insight into atmospheric Hg transport and deposition^83^. Total gaseous mercury (TGM) samples collected from an active air sampler in the Kangerlussuaq catchment show negative ${\Delta }^{200}{{{\rm{Hg}}}}$ and positive ${\Delta }^{204}{{{\rm{Hg}}}}$ for all samples collected throughout the season (Fig. 6c), attributed to atmospheric dry deposition processes and Hg(0) surface exchange^69^. In contrast, both fTHg and pTHg samples showed progressively higher ${\Delta }^{200}{{{\rm{Hg}}}}$ and lower ${\Delta }^{204}{{{\rm{Hg}}}}$ throughout the sampling period (Fig. 6c). Studies within the Arctic have demonstrated that Hg sources to snowpack and snowmelt are dominated by gaseous elemental Hg, which is further photo-oxidized during atmospheric mercury depletion events^84^. However, we do not see any overlap in Δ^200^Hg and Δ^204^Hg between our fTHg and pTHg samples and gaseous Hg collections. As Δ^200^Hg and Δ^204^Hg are relatively conservative tracers that cannot be modified by subglacial “dark” reactions, we conclude that the major atmospheric Hg source is snowfall or rainfall rather than gaseous elemental Hg.

High precipitation during the 2023 field season^24^ is likely responsible for the early-season even-MIF patterns, characterized by positive ${\Delta }^{200}{{{\rm{Hg}}}}$ and negative ${\Delta }^{204}{{{\rm{Hg}}}}$, and attributed to atmospheric processes that are consistent with Hg(0) oxidation followed by wet deposition^69,81^. However, we observe a shift in even-MIF in the late-season samples which may relate to the large proportion of supraglacial meltwaters in July (~50% of the total Hg pool; Fig. 6d). Here we hypothesize that during supraglacial ice melt, Hg(0) re-emission on the GrIS surface results in elevated even-MIF^82^ in our late-season samples (up to 0.20‰ ${\Delta }^{200}{{{\rm{Hg}}}}$ and −0.36‰ ${\Delta }^{204}{{{\rm{Hg}}}}$). While wet deposition is a source of Hg to the river, small-scale changes in even-MIF values may point to photochemical re-emission of Hg(0) in response to supraglacial UV irradiance, and subsequent enhanced glacial melt to the Qinnguata Kuussua, but further examination is required to elucidate processes and sources related to changes in even-MIF values.

### Assessing the influence of ice sheet Hg on fjord surface waters

**Fig. 7:**
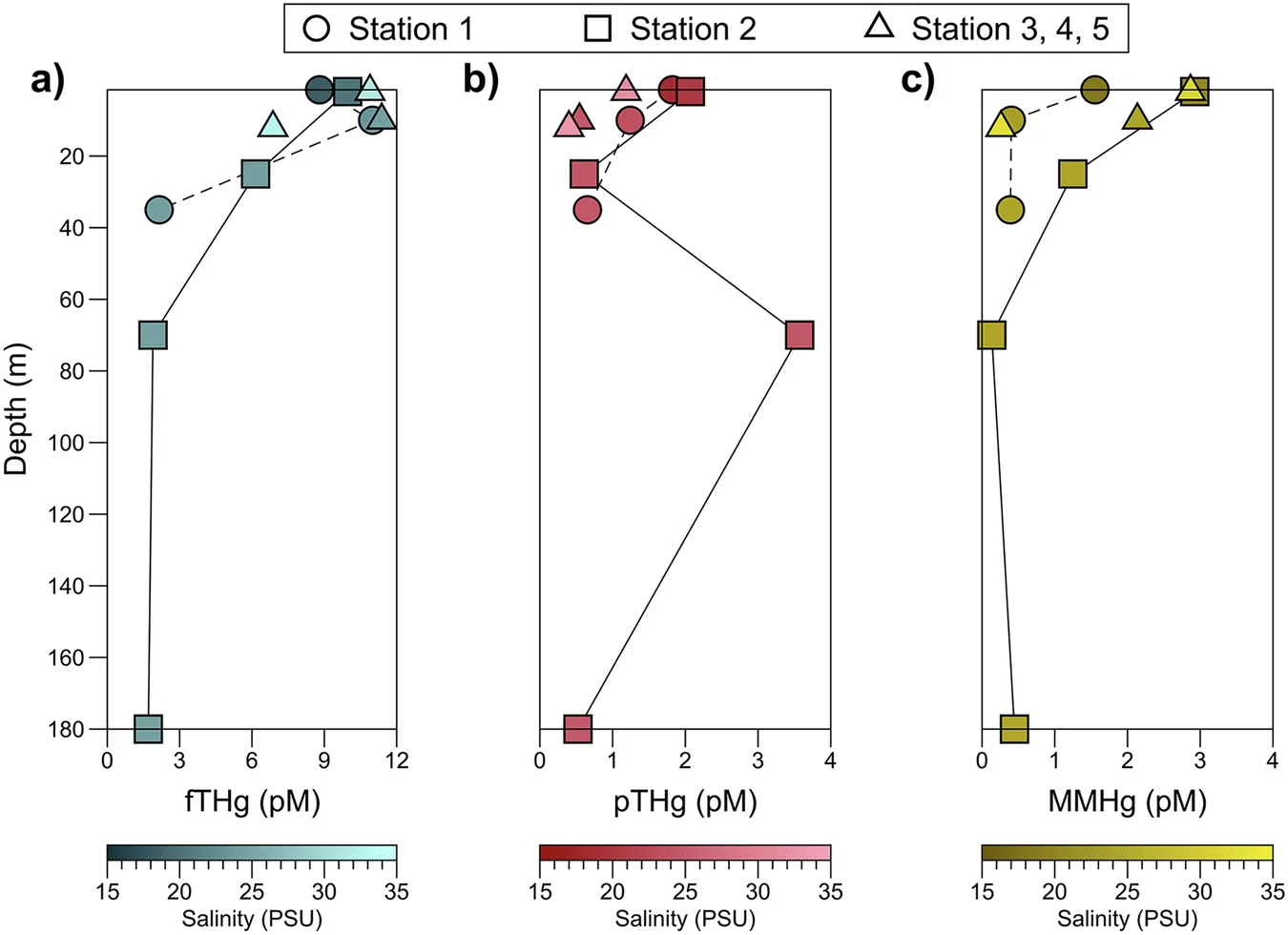
Hg concentrations versus depth (meters, m) for samples collected in Kangerlussuaq Fjord. **a** Filtered total Hg (fTHg). **b** Particulate total Hg (pTHg). **c** Monomethylmercury (MMHg). Shapes on all plots denote sample location, including station 1 (circles; partial depth profile, dashed line), station 2 (squares; complete depth profile, solid line), and station 3, 4, and 5 (triangles; surface samples only). Color gradients represent salinity, from dark (relatively lower salinity, <25 PSU) to light (>25 to 35 PSU).

In coastal waters, rivers are thought to be the largest source of Hg(II), accounting for ~45% of total Hg inputs and exceeding fluxes predicted for atmospheric deposition^85^. Hg concentrations in Kangerlussuaq Fjord indicate that glacial meltwater from the Qinnguata Kuussua may be a regionally relevant Hg source to coastal waters (Fig. 7). fTHg concentrations ranged from ~1.7 to 11.3 pM (Fig. 7a), with the highest values observed in surface waters (0–10 m), and lowest concentrations observed at depth (35–180 m). Fjord surface fTHg concentrations were on average three times larger than the seasonal average from the Qinnguata Kuussua (~3.1 ± 2.7 pM, *n* = 51; Fig. 2c). fTHg concentrations remained consistent between the fjord head and fjord mouth, indicating multiple sources and controls likely contribute to the dissolved Hg pool along the length of the fjord. Sources may include the desorption of pTHg delivered by glacial meltwater, as suspended particulates settle or are chemically transformed in the fjord mixing zone^86^. In addition to desorption, other inputs such as atmospheric exchange of Hg(0), proximal surface and subsurface runoff (e.g., from permafrost melt and flushed active layer)^87^, or groundwater inputs^88^, may contribute to fTHg along the length of the fjord. Elevated fTHg concentrations at the fjord surface relative to the Qinnguata Kuussua may therefore not be solely controlled by glacial meltwater contributions and necessitate further research.

pTHg concentrations in fjord surface waters were much lower (0.5–2.1 pM in range; Fig. 7b) than in the Qinnguata Kuussua (seasonal avg = 9.1 ± 2.8 pM; Fig. 2c). Fjord waters have lower SSC than glacial meltwater in general, with SSC averaging 0.68 ± 0.4 g L^−1^ in the Qinnguata Kuussua relative to ~0.01 ± 0.01 g L^−1^ in the fjord, indicating rapid sinking, dilution, or sorting of particulates upon entering fjord waters. This rapid change at the fjord head likely drives Hg transformations, and lower pTHg concentrations may point to Hg desorption from the particulate to the dissolved pool. One proposed mechanism for the release of pTHg is an increase in salinity (~31 PSU at station 1). Glacial meltwater appears to transport Hg-bound to negatively charged particles (organic matter or clay minerals, as indicated by the extractions; Fig. 2d), but once this material enters saline waters the rising concentration of chloride ions (Cl^-^), likely competes for pTHg adsorption sites^89^. Cl^-^ can form stable, soluble Hg(II) complexes and may be responsible for the higher fTHg observed in the fjord waters^86^. Additionally, Hg is expected to complex predominantly with strong thiol-containing DOC ligands in the marine environment^29,90^, and this may provide a complementary pathway for fTHg retention in fjord surface waters alongside chloride complexation.

MMHg concentrations observed in Kangerlussuaq Fjord followed fTHg trends, where the highest concentrations were observed in surface waters (0.5–2.1 pM in range) and declined with depth (Fig. 7c). Average fjord MMHg surface water concentrations (<10 m; ~1.9 pM avg) were more than double that of river concentrations (~0.5 ± 0.5 pM, *n* = 26) and were comparable to the highest MMHg values observed during P1 (~2.2 pM). These observations support the potential for methylation of inorganic Hg(II) at the fjord surface. Terrestrial inputs of Hg(II) (e.g., glacial rivers and permafrost melt) are likely an important source of Hg(II) to the fjord, as Hg(II) may desorb from particles and filtered Hg(II) can undergo microbial methylation in the water column^91,92^. Research in the Canadian Arctic estimates that the conversion of inorganic Hg(II) to MMHg accounts for ~47% of MMHg present in polar marine waters^93^. Outside the fjord (station 5; Fig. 1), Hg concentrations were lower, with fTHg and MMHg concentrations at 6.8 pM and 0.25 pM, respectively, further pointing to the importance of terrestrial Hg sources to fjord waters. The depth-dependent pattern in fjord observations here (Fig. 7) contrast that from the marine-terminating glacial Sermilik Fjord on the Eastern coast of Greenland, where Hg enrichment at depth is linked to glacial suspended sediments scavenging particle-reactive Hg(II)^94^. These differences may reflect varying fjord hydrography and imply that Hg dynamics in fjords may not be straightforward to scale^22,95^. For example, land-terminating glaciers deliver meltwater at the fjord surface, enhancing surface stratification and potential Hg phase transitions (e.g., pTHg desorption, photochemical redox transformations), whereas marine-terminating glaciers release subglacial meltwater at depth, promoting deep Hg-sediment interactions (e.g., trapping Hg below the surface and enhancing pTHg settling)^94^. Distinctions in fjord dynamics may influence Hg distribution, underscoring the need to consider geology and coastal inputs^96^, glacier type, and fjord hydrography when assessing Hg in fjord systems.

### The importance of the GrIS in the Arctic Hg cycle

We calculated the total Hg yield from the southwestern margin of the GrIS and compared it with reported values from glacierized catchments and non-glacial riverine systems. We estimate a southwestern GrIS total Hg yield of 23 mmol km^−2^ yr^−1^ (Table 1). This yield is more than an order of magnitude lower than that reported by Hawkings et al., (2021), but comparable to data published from six major Arctic rivers (Table 1; 4–20 mmol km^−2^ yr^−1^)^97^, suggesting that glaciers are important Hg sources to neighboring Arctic coastal waters. Discrepancies between studies underscore the challenges of accurately assessing Hg fluxes from dynamic glacierized catchments. The large concentration differences found between this study and previous work^12^, highlight the need to substantially revise previous Hg flux estimates going forward (Table 1), and indicate that these initial Hg flux estimates are likely overestimated, representing an outlier of Arctic observations. Notwithstanding, this study provides the best constraint thus far on particulate and filtered THg and MMHg concentrations from the GrIS across the melt season (1.6–17.5 pM fTHg, 0.–2.3 pM MMHg, and 4.8–17 pM pTHg) which are within the range of other Arctic waters^98^ and will help benchmark future Hg concentration measurements on the GrIS and other glacierized catchments. Changing hydrological and biogeochemical processes in glacierized catchments due to a warming climate could increase Hg fluxes to the Arctic Ocean by mobilizing Hg stored in ice melt, by accelerating subglacial weathering^99^, or by proglacial sources, such as permafrost melt^96^.

**Table 1:** Measured discharge weighted mean Hg concentrations, estimated fluxes, and estimated yields of filtered total Hg (fTHg) and unfiltered total Hg (uTHg)

Study Area	Catchment Size (km^2^)	Discharge (km^3^ yr^−1^)	Concentration (pM)		Estimated Flux (mol yr^−1^)		Estimated Yield (mmol km^−2^ yr^−1^)	
			fTHg	uTHg	fTHg	uTHg	fTHg	uTHg
Kangerlussuaq 2023 (this study)^a^	2800	5.1	2.5 (1.6–17.5)	12.5 (7.1–28.5)	13.2 (8.1–89.9)	64.3 (36.5–146.1)	4.7 (2.9–32.1)	23 (13.0–52.2)
GrIS Southwest Margin^93,94^^b^	230,000	122 (183–813)	2.5 (1.6–17.5)	12.5 (7.1–28.5)	305 (195–2135)	1,525 (866–3477)	1.3 (0.8–9.3)	6.6 (3.8–15.1)
Kangerlussuaq 2022^13^^c^	230,000	122	3.0 (1.1–6.2)	6.7 (5.1–9.9)	366 (134–756)	817 (622–1208)	1.6 (0.6–3.3)	3.6 (2.7–5.2)
Arctic Rivers^97^	ND	3,500	ND	29–82	ND	185,000	ND	4.0–20

^a^Mean concentrations for this study (2023) were calculated from the discharge weighted mean value, with the range of concentration (pM) reported in parentheses. Yield calculations were estimated using the estimated hydraulically active catchment area (2800 km^2^)^100^.

^b^Flux and yield estimates for the GrIS southwest margin were calculated using the average discharge (122 km^3^ yr^−1^)^124^ and the range of estimates reported in parentheses was calculated using total GrIS discharge^125^.

^c^Mean concentrations from Kangerlussuaq 2022^13^ were calculated using average fTHg and uTHg concentrations reported in the paper, rather than calculated relative to discharge^13^

Flux and yield estimates were calculated for this study using discharge measurements from GEUS in 2023^126^. Flux and yield estimates for Kangerlussuaq catchment in 2023 were then upscaled to the southwest region of the GrIS and compared to previously published data. Data from Jørgensen et al., (2024) was upscaled to the southwest GrIS margin comparatively. To compare our concentrations to six major Arctic Rivers, concentrations, fluxes, and yields are reported from Zolkos et al., (2020).

Upscaled to the GrIS southwest margin, our estimated flux of 1525 mol yr^−1^ Hg is approximately ~1% of the current riverine Hg flux estimate to the Arctic Ocean (estimates include 2 × 10^5^ mol yr^−1^ or ~1 × 10^5^ mol yr^−1^)^7,97^. Therefore, the GrIS exhibits Hg yields similar to those of Arctic rivers, but is a minor contributor to total Hg flux, owing mostly to differences in discharge between Arctic rivers and the southwestern GrIS. Yet, the GrIS appears to be an important Hg source to receiving coastal waters, but more extensive spatiotemporal datasets are needed to accurately include the GrIS in Arctic Hg budgets, particularly as meltwater production and export are predicted to increase over time in response to climate warming.

Proglacial landscapes and glacial meltwaters in southwest Greenland are Hg sources to coastal fjord waters. Using an ice-to-ocean transect, our study documents high temporal variability in Hg concentrations and sources linked to the evolving GrIS hydrological system and contribution of supraglacial and subglacial waters. Early-season Hg export was largely sourced from proglacial permafrost and subglacial inputs from distributed drainage systems, mobilized by episodic high precipitation, and was strongly correlated with dissolved organic carbon. As meltwater discharge increases, glacial Hg sources dominate the Hg contribution to the Qinnguata Kuussua. Hg stable isotopes reveal that subglacial weathering is a primary driver of Hg export. A Δ^199^Hg -based isotope mass balance linked seasonal glacial hydrology to Hg export, demonstrating that at peak melt, approximately half of the exported Hg is derived from photo-reduced surface waters, with the remaining Hg input from dark subglacial sources characterized by minimal mass-independent fractionation. Stable isotopes further indicate that total gaseous mercury deposition is not the major atmospheric source of Hg to GrIS glacial meltwater. In the fjord, Hg associated with glacially derived particulates appears to desorb and may be an important source of Hg(II) for methylation, with implications for bioaccumulation in surface waters. Concentrations of Hg in fjord surface waters remain elevated compared to river waters from the fjord head to the fjord mouth, suggesting Hg concentrations may not be solely controlled by glacial contributions. Our results indicate that the GrIS and the adjacent proglacial terrain are dynamic Hg sources to the Arctic coastal ocean, and that future climate-driven increases in GrIS melt will likely increase the contribution of ice sheet-derived Hg to the Arctic Hg budget.

## Methods

### Site description

Sampling took place in Kangerlussuaq, Greenland during June and July of 2023. Samples were predominantly collected from the Qinnguata Kuussua, which drains a ~ 2800 km^2^ catchment of the GrIS^100^ into Kangerlussuaq Fjord (Fig. 1). The Kangerlussuaq catchment from the ice margin to the fjord consists predominately of glacial till terrain and low Arctic tundra, underlain by thin permafrost-affected soils developed on glacial sediments. Fjord Hg concentration samples were collected from Kangerlussuaq Fjord, with an additional sample collected outside the fjord (station 5, triangle; Fig. 1), influenced by open ocean waters. All sampling sites overlay the Rae and North Atlantic Cratons, two geological provinces that encompass >50% of the proposed GrIS subglacial lithology^101^.

### Hydrological monitoring

Discharge data for Qinnguata Kuussua was collected by the Geological Survey of Denmark and Greenland (GEUS) as part of the Programme for Monitoring of the Greenland Ice Sheet (PROMICE) and the Greenland Climate Network (GC-Net)^102^. Precipitation data are provided by the Kangerlussuaq-DMI (Danish Meteorological Institute) atmospheric monitoring station, located at Kangerlussuaq Airport^103^.

### Riverine sample collection, processing, and storage

Daily water sampling (black square, Fig. 1) occurred between June 1 and July 21, 2023, at ~10:00 AM local time in the Qinnguata Kuussua (67.005°N, 50.684°W), approximately 25 km from the ice sheet margin. Samples were collected using a peristaltic pump with acid-cleaned platinum-cured silicone tubing placed into an active, flowing section of the meltwater river. Peristaltic pump tubing was rinsed for one minute with unfiltered water, and sample bottles were rinsed three times with sample before final collection. Hg water samples were collected into acid-cleaned 125 mL amber glass bottles, following clean-hands, dirty-hands methods^104^. Unfiltered total Hg (uTHg) samples were collected directly into sample bottles. Samples for filtered total Hg (fTHg) and filtered monomethylmercury (MMHg) were collected using a 0.45 μm capsule filter (Geotech dispos-a-filter™) with a PES (polyethersulfone) membrane. The filter was rinsed with ~1.5 L of sample prior to collection and a new filter was used for each sample collection. All Hg water samples were acidified with lab-distilled ultra-trace metal grade hydrochloric acid (HCl) to 0.4% v/v concentration in a laminar flow bench immediately after collection.

A 1 L high-density polyethylene (HDPE) bottle was used to collect unfiltered water for sequential particulate Hg extractions (Supplementary Text 5 and Table S10). Water was passed over a 47 mm 0.45 μm PES membrane (Millipore Express® Plus) until blocked (~250 mL). Filters were stored in petri dishes and kept frozen (−18 °C) until analysis.

Synoptic samples for total Hg (uTHg and fTHg) were also taken around the Qinnguata Kuussua catchment to assess the Hg composition of endmembers. Synoptic samples include glacial meltwater, proglacial lakes, and a permafrost melt stream (Table S2). Glacial meltwater samples were collected within 2 km of each glacial margin. All sample collection and processing were identical to the daily samples (Supplementary Text 2).

An AquaTroll 600 multiparameter sonde was used to measure water quality parameters, including specific conductivity (SPC). Details on these parameters and measurements for suspended sediment concentration (SSC) can be found in Supplementary Text 1 and Tables S6 and S7.

### Mercury stable isotope collection, processing, and storage

Hg stable isotope samples were collected every ~5 days or during periods of distinct hydrological change at the primary river sampling site. For each sample, 30 L of water was filtered through 0.45 μm PES capsule filters (~5 L per filter) into pre-cleaned (24 h, 2% v/v HCl) 5 L polyethylene terephthalate glycol bottles^105^. The capsule filters used for sample collection were pumped dry (i.e., all remaining water was evacuated from inside the capsule) and stored frozen to be digested for suspended particulate Hg isotope measurements. Immediately after collection, the filtered water samples were preserved and oxidized with 50% trace metal grade nitric acid (HNO_3_) and concentrated bromine monochloride (BrCl; prepared as in EPA 1631), both to 1% v/v concentration. Samples were stored in the dark and digested for 6 days prior to passing through an ion-exchange (AG1-X4) resin^106^. The sample was pumped through a fluorinated ethylene propylene (FEP) column containing 3 mL of pre-treated AG1-X4 resin^106^. Following sample loading, the resin was rinsed with ultrapure water and 0.1 M trace metal grade HCl before Hg was eluted using 30 mL of a 0.05% L-cysteine and 1% sodium citrate solution^107^. The elution solution was preserved with 3 mL concentrated BrCl and stored in the dark. All sample pre-treatment and column chemistry was performed in the field laboratory in Kangerlussuaq.

### Supporting geochemical analytes and field process blanks

For supporting information, a suite of biogeochemical analytes, including dissolved organic carbon (DOC), total nitrogen (TN), major ions (Ca^2+^, Mg^2+^, K^+^, Na^+^, Cl^-^, F^-^, SO_4_^2-^), macronutrients (nitrite+nitrate (NO_2_^-^ + NO_3_^-^), ammonia (NH_3_), soluble reactive phosphorus (SRP)), dissolved silicon (DSi), and particulate organic carbon (POC), were collected at the daily terrestrial sampling site and at all synoptic sample sites. Collection and analytical methods for these analytes are provided in Supplementary Text 2 and 3. Data for these analytes are provided in Table S1. Information on field blanks can be found in Supplementary Text 2 and Table S9.

### Atmospheric mercury

MerPAS passive air samplers^108^ and an active air sampler^109^ were deployed in the field for the assessment of total gaseous mercury (TGM) concentrations and atmospheric Hg stable isotopes. Additional information on atmospheric sample collection, analysis, and results is provided in Supplementary Text 4, Supplementary Fig. 2, and Table S4.

### Fjord sample collection, processing, and storage

Fjord samples for Hg concentration were collected in early July from the Greenlandic vessel M/V Tulu. Water samples were collected using 8 L GO-Flo (General Oceanics) bottles hung from a non-metallic line and triggered by messenger. Conductivity-Temperature-Depth (CTD; RBR Maestro) measurements were obtained at the same stations (Table S8). After collection, water was drawn from the GO-Flo bottles in a shipboard laboratory within a clean bubble (MAC10 HEPA filtered air) and filtered through an acid-washed 0.4 μm Nuclepore filter for fTHg and MMHg. Samples for THg were stored in acid-washed glass bottles (iChem) and for MMHg in acid-washed PET bottles (Nalgene). All samples for fTHg and MMHg were acidified with HCl to a 0.5% v/v concentration. The filter for particulate Hg (pTHg) was preserved by freezing. Separate sample aliquots were tested for dimethylmercury, which were below detection.

### Total mercury sample analysis

Riverine samples were analyzed at the University of Pennsylvania. Briefly, 125 mL filtered and unfiltered water samples for total Hg were oxidized with 1% BrCl and analyzed using EPA method 1631 on a Tekran 2600^110^. Analytical blanks were below the detection limit (assessed as 3 s.d. of the reagent blank; <0.20 ng L^−1^ or <1.0 pM). All concentrations of fTHg and uTHg exceeded the methodological detection limit. Filtered and unfiltered field procedural blanks were all <0.30 ng L^−1^ (<1.50 pM, Table S9). An ongoing precision and recovery (OPR) spike of 1.0 ng L^−1^ Hg (~5 pM) was tested every 10 samples, with a long-term precision of ±13.6% (RSD). Duplicate measurements and sample spikes of 1.0 ng L^−1^ were run every 10 samples, with spike recoveries ranging from 83% to 124% and duplicate precision between ±0.3% and ±6.7% (RSD). Particulate total Hg (pTHg) was calculated in each water sample by subtracting the filtered fraction from the unfiltered, whole water fraction (uTHg – fTHg = pTHg). For data analysis, pTHg concentrations are compared to fTHg concentrations, in which the addition of the two equal the total Hg concentration (THg).

Fjord samples were analyzed for filtered and particulate THg at the University of California, Santa Cruz. Fjord fTHg samples were digested at room temperature at a final concentration of 0.5% BrCl for >8 h. Particulate sample filters were first digested in 2 M HNO_3_ for 4 h in a 60 °C water bath and then treated as the fTHg samples. Both sample types were analyzed on a Tekran 2600, calibrated by both saturated vapor injections (Tekran) and with an aqueous standard (Spex). Detection limit, assessed as 3 s.d. of the reagent blank, was 0.20 pM for a 50 mL filtered sample volume and 0.01 pM for a 1 L particulate sample volume. Sample recovery was assessed through repeated determination of a standard reference material (DORM-4) digested as the particle samples, which showed a typical range of 92–108% of the certified value.

### Monomethylmercury determination by solvent extraction

River and fjord MMHg concentrations were determined using direct ethylation and isotope dilution^111–114^. In brief, waters were digested with 1% sulfuric acid for >24 h following spiking with MM^198^Hg (concentration 0.967 fmol µL^−1^; 967 pM). The volume of spike added to each sample was set to ~10% of the measured fTHg and the mass of the spiked sample was recorded to determine the exact sample volume. After equilibration, 5% ascorbic acid^115^ and 2 M acetate buffer were added and the samples were derivatized using sodium tetraethylborate at ~pH 4 (pH adjustment with 12 M potassium hydroxide, KOH). The derivative was extracted into about 500 µL of hexane after shaking for ~ 20 min^113,116^. The hexane solvent layer was removed using a 1000 μL pipette, transferred to a pre-cleaned (24 h HCl) 2 mL gas chromatography (GC) vial, and stored at 4 °C until analysis via gas chromatography inductively coupled tandem plasma mass spectrometer (GC-ICP-MS/MS; Agilent 8890-8900). Analysis on the GC-ICP-MS/MS followed standard operating procedures, including the use of a bulk standard and certified reference material for calibration and quality control. The detection limit was ~10 fM for a 200 mL sample.

### Hg stable isotope measurements

Hg stable isotope analyses were performed at the USGS Mercury, Metals, and Microplastic Research Lab (M^3^RL), Madison, WI. Capsule filters for pTHg were removed from the plastic housing and digested in 30% aqua regia (3:1, HCl:HNO_3_) for 5 days at 55 °C. Both pTHg and resin elution solutions for filtered water samples were pre-concentrated for isotope analysis using stannous chloride reduction and gold amalgamation, coupled to rapid thermal desorption into a chemical oxidant trap (40% 3:1 HNO_3_: BrCl)^117^. Average percentage recovery for water samples was 100 ± 5.3% (*n * =  10) and 98 ± 7.6% (*n* = 11) for particulate filter digests. A reference Hg isotope ratio standard (National Institute of Standards and Technology [NIST] 3133) was purged and trapped during each analytical batch (100 ± 3.4%, *n* = 16) to ensure isotope ratio accuracy.

Hg stable isotope ratios were determined using a multi-collector inductively coupled plasma mass spectrometer (MC-ICP-MS, Thermo Scientific Neptune Plus). Solutions were introduced using stannous chloride reduction coupled to a gas-liquid separator^118^. Thallium (40 ng mL^−1^) was used for mass bias correction and was simultaneously introduced into the gas-liquid separator as an aerosol using an Apex-Q desolvating nebulizer. Hg isotopes were analyzed following standard-sample bracketing protocol^119^ using NIST 3133. A secondary standard NIST RM8610 was analyzed every five samples to ensure data accuracy and precision. All quality control and assurance data can be found in Table S5, in the Source data file. Data were calculated in delta notation according to convention^119^, in which δ^xxx^Hg represents mass-dependent fractionation (MDF) and Δ^xxx^Hg represents mass-independent fractionation (MIF). See the Source data file for analytical precision and recovery of processing standards (NIST 3133) and secondary analysis standards (NIST RM8610).

### Hg sediment extractions

Sequential extractions were used to assess chemical lability of Hg within particulate matter. Suspended sediment samples collected on PES membrane filters were subjected to a four-stage sequential extraction to characterize the lability of the pTHg fraction following an established method^120^. Analytical methods and results for sequential extractions are provided in Supplementary Text 5.

### Statistical analysis

Principal Component Analysis (PCA) was performed using R Studio (version 2024.12.1 + 563)^121^. Methods for the PCA and PCA data visualization are provided in Supplementary Text 6 and Fig. 3. A simple linear regression was performed in Excel to examine the relationship between two variables, and statistical significance was evaluated at a *p* < 0.05.

### Endmember selection for Hg stable isotope mixing model

To estimate the contribution of Hg from subglacial sources, a binary mixing model using Δ^199^Hg was employed, similar to the approach used in other glaciated systems^80^. Endmembers of weathered rock and supraglacial meltwaters were not available due to sampling constraints and lack of previous measurements, so proxy values were used. In the case of subglacial Δ^199^Hg values, we used average Precambrian bedrock values from the literature (average Δ^199^Hg = −0.01 ± 0.16‰, Table S3), which were the same age and rock type as our sampling location in Greenland. Data on supraglacial sources of Hg were highly limited in the literature. The geographically closest candidate for endmember samples were one snow measurement from Greenland (Δ^199^Hg = 0.32‰)^81^ as well as firn and ice core samples from the Canadian Arctic from post-industrial (Δ^199^Hg = 0.08 ± 0.28‰) and pre-industrial (Δ^199^Hg = 0.28 ± 0.83‰) periods^79^. However, measurements of Δ^199^Hg in fTHg from Qinnguata Kuussua greatly exceed these values, particularly in late season, thus these published values cannot be used as endmembers. Instead, we opted for Δ^199^Hg values in snow as a proxy, but were limited in selection of published values because we saw no isotopic evidence of atmospheric Hg depletion events in our measurements, unlike the highly negative MIF values previously reported in Arctic snow^84^. Due to these limitations in endmember designation, we selected a mid-latitude snow Δ^199^Hg value (3.50‰)^73^. These values were similar, albeit lower, when compared to glacial surface ice samples measured in the Tibetan plateau^65^, but represented a more conservative value for mixing model calculations.

## Supplementary information

Transparent Peer Review file

Supplementary Information for Mercury mobilization and export from the Greenland Ice Sheet using an ice-to-ocean approach

Description of Additional Supplementary files

## Source data

Data Source File

## Data Availability

Discharge data in this manuscript was collected by the Geological Survey of Denmark and Greenland (GEUS) as part of the Programme for Monitoring of the Greenland Ice Sheet (PROMICE) and the Greenland Climate Network (GC-Net)^102^. These data are openly available from GEUS Dataverse under the PROMICE/Watson River Discharge collection (https://doi.org/10.22008/FK2/XEHYCM)^122^. Precipitation data in this manuscript was provided by the Kangerlussuaq-DMI (Danish Meteorological Institute) atmospheric monitoring station^103^ and were downloaded from the DMI “Download Data” Climate Data portal (https://www.dmi.dk/friedata/dokumentation/download-data). All data used in this manuscript, including processed discharge and precipitation data, hydrological measurements, mercury concentration and speciation data, mercury stable isotope data, atmospheric mercury measurements, fjord hydrographic data, and the numerical results underlying the graphs and charts in the main figures and Supplementary Information, have been deposited in the National Science Foundation (NSF) Arctic Data Center (https://doi.org/10.18739/A2JW86Q20)^123^ and are available publicly without restriction. Source data for all main-text figures and Supplementary Figs. are also provided in the accompanying Source Data file and are identical to the files archived in the repository. If any additional information or clarification on the datasets is required, data queries should be directed to the corresponding author (Amina Youssef, aminay@sas.upenn.edu).
