# Supplementary material for: Mercury mobilization and export from the Greenland Ice Sheet using an ice-to-ocean approach
Source: Commun Earth Environ. 2026 Jul 16;7(1):800. doi: 10.1038/s43247-026-03818-z (PMC13626945; doi:10.1038/s43247-026-03818-z)
Supplement: Supplementary file 1 [file 43247_2026_3818_MOESM1_ESM.pdf]

Peer Review File

Mercury mobilization and export from the Greenland Ice Sheet using an ice-to-ocean approach

Corresponding Author: Ms Amina Youssef

This file contains all reviewer reports in order by version, followed by all author rebuttals in order by version.

Attachments originally included by the reviewers as part of their assessment can be found at the end of this file.

Version 0:

Reviewer comments:

Reviewer #1

(Remarks to the Author)
Please see the attached PDF file.

Reviewer #2

(Remarks to the Author)
The topic and conclusions of this article are interesting, and this study aim to address a key issue worthy of investigation in the Arctic Hg cycling. However, the majority of the manuscript focuses on discussing the biogeochemical processes of mercury during glacial meltwater transport, while the sample collection and discussion of the to-ocean process are very limited.I understand this is hard to work in the Arctic regions. I still suggest to the authors should better align the main conclusion in more soft tune. The discussion of mercury concentrations is entirely isolated and disconnected from the discussion of mercury isotopes. Several conclusions drawn from the mercury concentration data may be not supported by the mercury isotope data. Therefore, the authors should reconcile the features from both mercury concentrations and mercury isotope data to derive robust and comprehensive conclusions.
The selection of the two end-members for the mixing model calculation is highly controversial. I do not agree with the authors' assumptions regarding these end-members. In fact, the best approach would be for the authors to measure the mercury isotopic signatures of supraglacial meltwater and of subglacial meltwater immediately upon exiting the glacier. The authors need to verify these issues for the signatures of endmemeber. The transition from glacier to fjord lacks sufficient samples, particularly for mercury isotopes. I understand it is very hard to work at these stations. But I still worried about this issue that the revier spans a considerable distance (>100 km), meaning that about 90% of the purported "ice-to-ocean" pathway is missing from the analysis.
Line 51: How can glacial processes further mobilize Hg from atmospherically deposited Hg? Further explanation of the mechanism is needed.
Line 75: I need to know the distance between the spot samples on the glacier surface and plot 4, which appears to be roughly 150 km, as well as the types of soil and vegetation along this transect (e.g., wetland, tundra, grassland). This information is crucial.
Line 106-117: The caption for Figure 2 is overly complex. It should be simplified, retaining only the essential information.
Line 114-117: Is Filtered total Hg equivalent to Dissolved Hg (≤ 0.45 μm)? For subplot (d), which location in Figure 1 do the mercury concentrations correspond to? The time series location?
Line 121-123: Which sampling site is being referred to?
Line 157-159: The explanation for the higher %MMHg is unconvincing and requires further elaboration. Also, explain why the %MMHg is significantly lower during the early period.
Line 159-164: If, as the authors state, "MMHg showed a strong correlation to DOC in the early season," then why is the %MMHg extremely low during period P1 when DOC levels are high? The authors are advised to explain these processes from the perspective of mercury cycling mechanisms, rather than providing a mechanistic interpretation of correlations based solely on numbers.
Line 187-189: I do not fully agree with this statement. In my view, MMHg concentrations remain at relatively low levels throughout (with few exceptions). The authors should enhance the discussion from a geochemical perspective, rather than merely describing the data.

Line 192-199: The authors' inference regarding the source of MMHg in the river water, attributing it to the glacier, is overly simplistic and unconvincing. Further evidence, such as from isotopes, is recommended. Processes like other surface runoff or subsurface seepage within the Qinnguata Kuussua catchment are more likely sources of MMHg.
Line 210-211:that exported pTHg is sourced primarily from subglacial physical weathering processes." Why? Provide more evidence.
Line 257: In high-melt years, supraglacial meltwater will also increase, potentially at a rate exceeding that of subglacial meltwater. The authors should conduct a comprehensive discussion considering multiple factors, rather than focusing solely on the effects of increased subglacial meltwater. Therefore, Lines 259-266 should be reconsidered and rewritten to account for these various influences.
Line 275-278: This paragraph does not convey any original viewpoint from the authors in this context. It is recommended for deletion or relocation to the Introduction.
Line 289: This statement is overly absolute.
Line 290-298: As the authors themselves state, MDF could occur due to redox transformations, volatilization, kinetic, and biological reactions. Therefore, they should not use MDF signatures for qualitative source apportionment, as this is inaccurate and one-sided.
Line 300-301: This statement is inconsistent with the results shown in Figure 6A.
Line 303-304: This statement contains a clear error. There have been some reports on mercury isotope measurements in glaciers.
Line 319: I do not understand why the authors cite the mercury isotopic signatures of pre-Cambrian rock and Hg(II) in mid-latitude snow. In my opinion, these are clearly unrelated to mercury sources in glacial-river water. The selection of end-members is critical, and the authors should be more careful.
Line 332-334: Additional references should be included.
Line 352: A range of -0.43 to -0.07 is very large. The authors' explanation does not support their conclusion that "This supports the interpretation that pTHg originates from bedrock weathering."
Line 358: This assumption by the authors is far-fetched. I do not fully agree with the assumption that supraglacial GrIS meltwater can reach a $\delta^{202}Hg$ value of 3.5‰. I recommend that the authors add actual measured data for this. I note that in Figure S1, the mercury concentration in the supraglacial sample is not very low, especially for fTHg, which suggests the proportion of photochemical reduction might not be high. The authors should consider this comprehensively.
Line 371: I do not agree with the two end-member characteristics assumed by the authors. It is recommended to select appropriate end-members and rewrite the entire mercury isotope discussion accordingly. Also, there is a clear error in this formula; the denominator should not be pTHg, but rather the signature of the subglacial meltwater end-member.
Line 403-420: This conclusion is absurd. I find it completely incomprehensible.

Reviewer #3

(Remarks to the Author)
This manuscript is a large study on the mobility and transformations of Hg in the southern region of the Greenland Ice sheet. The study shows that Hg is released both from weathering under the glacier and from the glacial meltwater and that Hg is released into the marine fjord over the course of the northern summer season. This is a novel study using both traditional biogeochemical (measurement of Hg concentrations and discharge allowing a mass balance analysis) and newer, advanced stable isotope approaches. This will be of great interest to those in the field and a wider audience, especially given the recent focus on the Arctic under a warming climate. The conclusions are original. The methods are appropriate and convincing with no further evidence required to support the authors' conclusions. My main suggestions are for increased clarity around the definition of melt timing and details on speculated processes on how MMHg is produced in the fjord. Please see below for detailed comments.

Line 61 and 64. It appears that the river's name is presented as the Indigenous name, with the colonizers name in brackets in line 61. In line 64 (and throughout the manuscript), it appears the colonizer's name for the Fjord is listed first. If this is the case, I suggest that Greenlandic names are used as a priority over colonizer's names.

Fig 1. Defines blue areas as Ocean. However, it appears that blue areas also include lakes and rivers. Change the legend to reflect all water-bodies.

Fig 2 panels are out of order. Fig 2d (line 174) is presented prior to 2c. (line 204)

More clarity is needed on what is happening early vs. late with respect to melt prior to mention in the Fig. 5 caption. The discharge data suggest that melt water started to be measured around day 175, approx. 10 days after the definition of late at about day 163. Why is "late" defined as day 163?

Fig. 6. Gorgeous figure and this is really minor, but it seems more intuitive to order the colours from light to dark over the season.

Line 455. I am not clear on the process by which fHgII and pHgII undergo desorption and are converted to MMHg. More clarity on this would be helpful. Are authors suggesting abiotic methylation? How?

Table 1. Please define uTHg in the table. It is not defined until line 553 in the text.

Line 566. Better define and clarify "synoptic samples". I do not know what this means.

Line 578. Clarify "… filters used for sample collection were pumped dry…" I do not know what this means.

Line 584. Define FEP

Line 604 – remove "from" or "using"

Version 1:

Reviewer comments:

Reviewer #1

(Remarks to the Author)
Adequate revisions have been made to the manuscript to resolved the most important issues identified in the initial review.

Reviewer #2

(Remarks to the Author)
The atuhors have well addresses my concerns, and I suggest to accept the manuscript.

Reviewer #3

(Remarks to the Author)
The authors have addressed all of my concerns and comments appropriately.

Open Access This Peer Review File is licensed under a Creative Commons Attribution 4.0 International License, which permits use, sharing, adaptation, distribution and reproduction in any medium or format, as long as you give appropriate credit to the original author(s) and the source, provide a link to the Creative Commons license, and indicate if changes were made.
In cases where reviewers are anonymous, credit should be given to 'Anonymous Referee' and the source.
The images or other third party material in this Peer Review File are included in the article's Creative Commons license, unless indicated otherwise in a credit line to the material. If material is not included in the article's Creative Commons license and your intended use is not permitted by statutory regulation or exceeds the permitted use, you will need to obtain permission directly from the copyright holder.
To view a copy of this license, visit https://creativecommons.org/licenses/by/4.0/

Reviewer #1 Comments:

1 - Brief summary

* What are the major claims of the paper?

The study set out to characterize Hg sources, transport and export in meltwater from the Greenland Ice Sheet (GrIS) using a combination of repeated daily sampling of meltwater samples at a sampling station in Kangerlussaq, West Greenland, spot samples at a number of meltwater rivers, spot samples of glacier surface, spots samples of atmospheric deposition and marine samples in a transect along Søndre Strømfjord.

The main claim of the manuscript (MS) is that high temporal variability in Hg concentrations in glacial meltwater over the period 1 June 2023 to 21 July 2023 can be linked to the evolution of the Greenland Ice Sheet (GrIS) hydrological system and relative inputs of supraglacial and subglacial meltwater (ll 509-511), indicating a seasonal shift in the source of Hg from early melt season inputs of Hg from the terrestrial environment to a more pure subglacial sources at a more mature stage of the melt season.

Data and results of the MS share similar characteristics with two other recent studies conducted in the same study area in Western Greenland (Hawkings *et al.*, 2021 [ref.11 in the MS]; Jørgensen *et al.*, 2024 [ref.12 in the MS]) although the reported types of samples are expanded with both stable Hg isotopes and air samples.

Ref.11: Hawkings, J. R. et al. Large subglacial source of mercury from the southwestern margin of the Greenland Ice Sheet. Nat. Geosci. 14, 496–502 (2021).

Ref.12: Jørgensen, C. J. et al. Large mercury release from the Greenland Ice Sheet invalidated. Sci. Adv. 10, eadi7760 (2024).

Based on mean concentrations from the main sampling station in Kangerlussuaq (see picture from Google Earth below), a total Hg yield estimate for the southwestern margin of the GrIS is calculated based on simple multiplicational upscaling.

The relative contribution of geogenic and atmospheric sources contributions are investigated using analysis of Hg stable isotopes showing indications that subglacial weathering of bedrock could be a potential source of Hg. The MS concludes that the GrIS is a considerable Hg source and that climate-warming will likely increase the contribution of ice sheet derived Hg to the Arctic Hg budget.

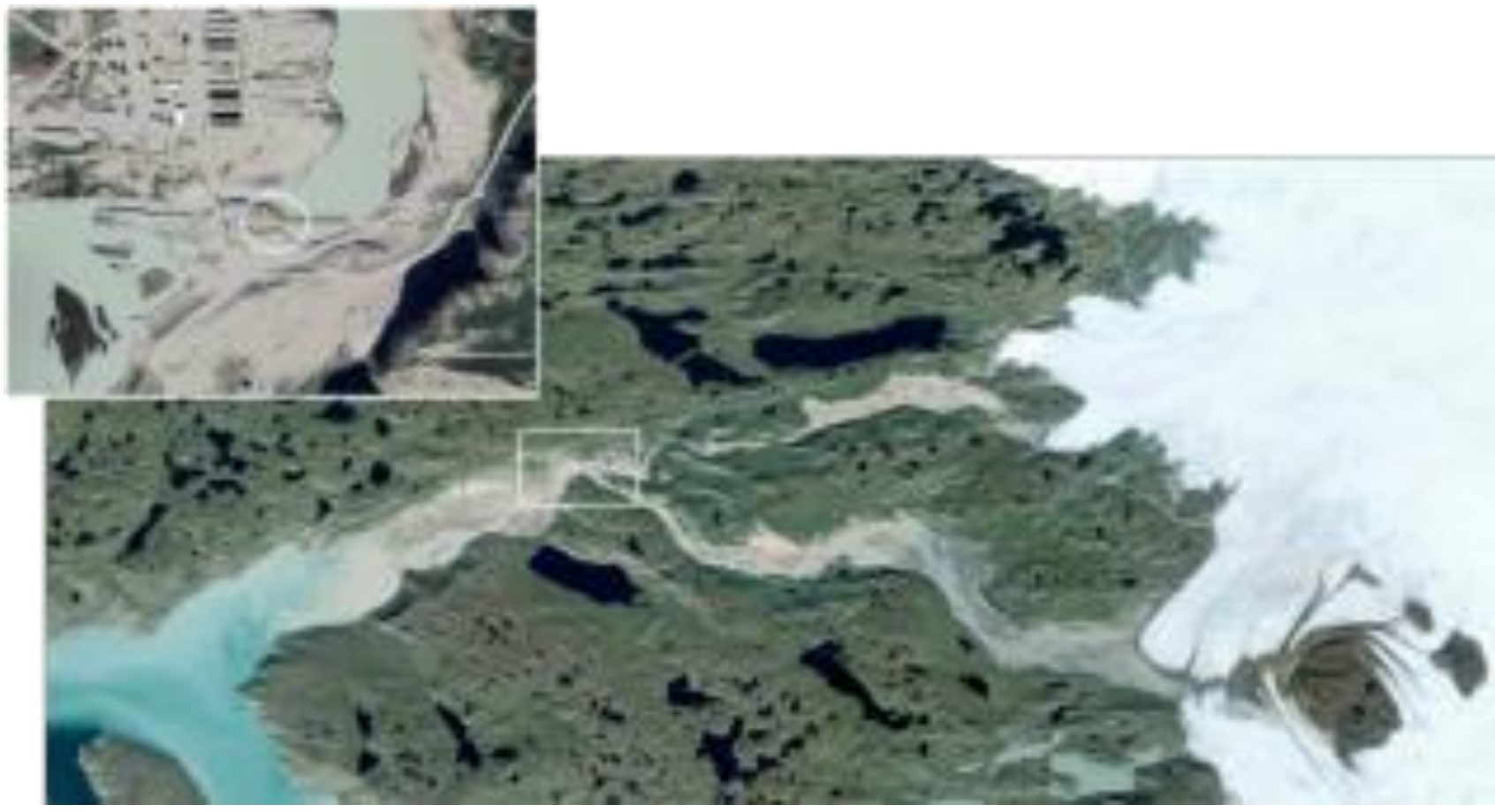

Picture from Google Earth showing location of primary sampling station, which is identical to the sampling station "Ctrl 4" of [ref.12].

At its core, the MS can be regarded as a data report providing additional results of "filtered dissolved mercury (fTHg)" and "monomethylmercury (MMHg)" at sampling stations more or less identical to [ref.11 and 12] supplemented by measurements of Hg stable isotopes, a few atmospheric Hg concentrations samples and water samples from 4 stations along Søndre Strømfjord. Based on this, the MS then seeks to explain the seasonal trend in Hg export to the ocean by invoking various sources of Hg with an assumed sensitivity to climate warming (i.e. terrestrial sources/ runoff from active layer permafrost, geogenic weathering below the GrIS, supraglacial sources/ atmospheric deposition).

The reviewer suggests the manuscript is a data report on filtered total Hg (fTHg) and monomethylmercury (MMHg) samples from similar locations to previously published work. While the discrepancies between the two previous studies provided by the reviewer here are accurate, our manuscript is a rigorous analysis of new data that aims to reconcile previous discrepancies by employing new measurements (most notably Hg stable isotopes). We strongly disagree with Reviewer #1's characterization of the study, which is corroborated by assessments by Reviewer #3 that highlight the novelty and importance of the work. We present a time series of total Hg concentrations, including filtered and <u>particulate</u> total Hg concentrations, as well as MMHg and critical biogeochemical constituents (e.g., DOC), across the span of two months, where there are multiple hydrological shifts so we can better understand the processes controlling Hg concentrations and speciation. In addition to this time series, spot samples are collected throughout the catchment area across a spatial and temporal scale, including at all accessible glacier outlet portals, proglacial lakes, a permafrost melt stream, and supraglacial surface meltwater samples. We also, to our knowledge, provide the first riverine time series of Hg stable isotope ratios in the Arctic. Hg isotope collection involved 30 L sample volumes and a 10-day processing period in the field, including 5 days of dark Hg oxidation followed by 5 days of pre-concentration onto an activated resin and 1 day sample elution. Given the extensive protocol for Hg isotope collection, select samples were collected that target distinct periods in the hydrograph, and equate to approximately one isotope sample every 5 days that supplement our Hg concentration time series. In addition, we provide an extensive geochemical and hydrological dataset on which to contextualize our findings.

Beyond river water sample collection, this study also provides new atmospheric Hg concentration data across spatial scales. We also provide sequential sediment extractions, a fjord transect comprised of 5 surface water stations (with Hg concentration samples collected at each station), 1 complete fjord depth Hg concentration profile, and 1 partial depth Hg concentration profile from the fjord head to the fjord mouth, with the addition of one sample outside of the fjord. At its core, this manuscript contains a complementary set of novel data, collected across a relatively remote and inaccessible region of the Arctic, that are rigorously interpreted. In fact, 65% of the manuscript is dedicated to the discussion of data, which is counter to Reviewer #1's characterization of this being a data report. We have considered all comments below from Reviewer #1 and revised the manuscript to address these comments.

2 - General assessment and comments

* Are these claims novel, and will they be of interest to others in the community? If the conclusions are not original, it would be helpful if you could provide relevant references.

The main claims of the MS are not particularly novel as time series of dissolved and particulate Hg in glacial meltwater from the same specific watershed have previously been presented and subsequently debated in scientific literature [ref.11,12]. Highly conflicting Hg concentrations were reported from the area in these two studies, where [ref.11] claims that Hg concentrations in meltwater from the GrIS is extremely elevated, which is disputed in [ref.12] in which low Hg concentrations in a similar range to the current study was reported.

The reviewer claims the manuscript is not novel because time-series analysis has been conducted in this region previously. A time series is a collection of data points recorded and measured at successive and uniform intervals of time. It is used to track changes over time, identify trends, and forecast future behavior. Although limited sampling campaigns have been conducted in this part of Greenland, we attain that these do not amount to significant time series. No mercury time series spanning multiple weeks, over distinct hydrological transitions has been conducted in this area of the Greenland Ice Sheet. Ref 11. (Hawkings et al.) sampling took place mid-summer, and Ref. 12 (Jørgensen et al.) occurred late August/beginning of September, bar some limited spot sampling. Neither reference captured hydrological changes in the melt season. Hawkings et al. occurred during subglacial drainage onset, missing the beginning of the melt season and hydrological transitions. Jørgensen et al. sampling was mostly at the very end of the ablation season, capturing the transition from peak ablation towards hydrological shutdown.

Here we capture distinct hydrological changes in the Greenland Ice Sheet over 2 months, as documented in Figure 2 and discussed in depth in the discussion section (e.g. early to late season developments / dynamics in meltwater production and accumulation). We observed large changes in Hg concentration driven by proglacial inputs in the early season to subglacial-dominated inputs in the late season. Additionally, we used Hg stable isotopes to assess the sources of Hg to the catchment area. Hg stable isotopes have never been published in Greenland glacial meltwater, making this study a novel use of an informative method for Hg source tracing.

As previously discussed, [ref.11, ref.12], an extremely elevated Hg yield from the GrIS could have far reaching consequences for both indigenous communities and the environment in

and around Greenland. The primary scientific knowledge gap needing resolution would appear to be the independent scientific verification of Hg levels in glacial meltwater from the GrIS to either support or reject the anomalously high Hg yields of [ref.11], in which several of the co-authors of the current MS where directly involved in.

In case the new data should support a rejection of the conclusions of the first controversial study (as turned out to be the case based on the 2023 data), the appropriate focus of a follow up paper from the author group of [ref.11] would be an in-depth discussion of these significant discrepancies, with a clear declaration on the validity of the initial predictions [ref.11] which were based on a Hg sampling strategy different to the current.

The reviewer suggests supporting a rejection of the conclusions of Ref 11. (Hawkings et al.). We strongly disagree that the focus of the study should be on rejecting conclusions of a previous study. Scientific discourse does not require focused rebuttals of previous publications. The goal of this independent study was to provide our own process-based information on the sources and fate of Hg in Greenland, not to discuss or refute previous studies. We provide comparative information and discuss differences in our discussion section (now starting on LN 512, section title "The importance of the GrIS in the Arctic Hg cycle"), along with a revised flux and yield estimate based on this new data. Although concentrations were much lower than those in Hawkings et al., the concentrations reported in here are higher and analyzed to a far greater extent (see responses above) to that reported by Jørgensen et al. The concentrations reported here are from important hydrological changes and therefore also help indicate that enhanced ice sheet melt may provide additional Hg to the catchment in future years. This study was not conducted to add to the controversy but instead to assess the presence and the sources of Hg in southwest Greenland, given the current literature gaps. Our data indicate Hg concentrations are dynamic and change rapidly in glacierized systems. A range of concentrations may be observed depending on time of year, hydrological conditions, precipitation inputs, and meltwater volumes. Thus, we do not agree with equating low concentrations in Greenland streams with low importance, as noted by Jørgensen et al., but instead put the river data into a larger context that allows for a critical examination of significance. For clarification on this and per the reviewer's feedback, more information regarding Hawkings et al. concentrations and our new estimates have now been provided in the manuscript. See LN 520-527 for this additional information.

The importance and prevalence of this important scientific knowledge gap is illustrated by a recent study (note: non-peer reviewed preprint) testing the previously asserted high Hg yields from the GrIS in the Nuuk fjord (Thyrring et al, 2026). The main conclusion of this study is as follows:

"*Our results, together with other studies on Hg in Greenland's waters, sediments, and planktonic species, cannot reproduce the previously reported mercury contamination from glaciers, and* ***there is now substantial evidence against the claim that Greenland ice sheet has implications for Hg budgets and coastal ecosystems***."

Ref: Thyrring, J., Krause-Jensen, D., Olesen, B. & Bjerregaard, P. (2026) No mercury from Greenland glacial runoff in sessile coastal biota | Research Square https://doi.org/10.21203/rs.3.rs-8804831/v1.

The reviewer suggests the conclusions made by Thyrring et al., 2026 support the absence of Hg in Greenland and suggests our manuscript should better address the current scientific discourse about the prevenance of Hg in southwest Greenland. Unfortunately, the work shared by the reviewer (Thyrring et al., 2026) has not undergone peer review. Given the nature of the work, and importance of scrutiny of all work cited related to this topic, it would be premature to cite work that has not undergone peer review. If the study is published as a peer-reviewed article prior to the publication of this manuscript, we will integrate a comparison in our discussion.

However, the MS only very superficially discusses this knowledge gap (see elaboration below). While the sampling strategy and analytical methods of the MS appear to be appropriate and credible for the 2023 campaign, the MS fails to communicate and discuss the implications of its recent findings speaking clearly against the extreme concentration claims which parts of the author group have previously asserted to a broader audience in the scientific community. For this reason, I cannot recommend the publication of the results in its current format and recommend a significant revision prior to potential resubmission (see suggestions below).

* On a more subjective note, do you feel that the paper will influence thinking in the field in terms of either conceptual understanding or technological capability? Please feel free to raise any further questions and concerns about the paper.

To me it seems unthinkable that the author group of the current MS did not plan the sampling strategy of the 2023 field work, with the above-mentioned specific knowledge gap in mind. And due to the controversy following the publication of [ref.11], it would be scientifically appropriate for the author team to examine whether the new data corroborate or challenge the conclusions of that earlier study, which has been substantially disputed.

The reviewer suggests we examine whether the new data corroborate or challenge the conclusions of the earlier studies, which have been disputed. This section has now been revised per the reviewer's feedback. See LN 515-527: "This yield is more than an order of magnitude lower than that reported by Hawkings et al., (2021), but comparable to data published from six major Arctic rivers (Table 1; 4 – 20 mmol km^{-2} yr^{-1})[100], suggesting that glaciers are important Hg sources to neighboring Arctic coastal waters. Discrepancies between studies underscore the challenges of accurately assessing Hg fluxes from dynamic glacierized catchments. The large concentration differences found between this study and previous work[12], highlight the need to substantially revise previous Hg flux estimates going forward (Table 1), and indicate that these initial Hg flux estimates are likely overestimated, representing an outlier of Arctic observations. Notwithstanding, this study provides the best constraint thus far on particulate and filtered THg and MMHg concentrations from the GrIS across the melt season (1.6 – 17.5 pM fTHg, 0.1 – 2.3 pM MMHg, and 4.8 – 17 pM pTHg) which are within the range of other Arctic waters[101] and will help benchmark future Hg concentration measurements on the GrIS and other glacierized catchments."

We have now attempted to corroborate the findings of the earlier studies in this final section of the manuscript. We acknowledged the discrepancies with the earlier study (Hawkings et al.) by noting the higher concentrations, as well as discuss the conclusions made by Jørgensen et al.,

which we believed lacked nuance and detail. However, we strongly believe it is better to keep the tone measured and professional by emphasizing the uncertainty/complexity, and communicate the findings as such.

When used appropriately, the data series of the MS could significantly contribute to the consolidation of the most likely concentration range in dissolved Hg in glacial meltwater from the GrIS, provide additional indices on potential sources of Hg at and around the GrIS as well as provide an example of scientific adjustment and retraction of previous conclusions and assertions as new indisputable evidence is made available.

We agree with the Reviewer that the study does provide consolidation of concentrations in Greenland, and provides extensive detail into potential sources, fluxes, and sinks of Hg in the region. We have added content to the final section of the paper that clearly highlights the range of Hg concentration data reported across the summer melt season, which serves to inform future studies on the Greenland Ice Sheet and other glacierized catchments, now on LN 520-530.

It is clear from the MS, that the concentration levels of dissolved Hg during part of the melting season of 2023 (at the same sampling location as CTRL 4 in [ref.12]) indisputably confirms the concentration ranges reported in Jørgensen et al. 2024 [ref.12], and thereby clearly contradicts the results, conclusions and associated implications as reported in Hawkings et al, 2021 [ref.11]. A logical consequence of the current confirmation of the conclusion of [ref.12] would appear to be an in-depth discussion on the potential causes for the differences between the results of [ref.11] and the current study, including self-critical reflection on applied sampling approaches, methods etc.

The main conclusion of Ref. 12 (Jørgensen et al.) is: "meltwater poses only a very limited risk for both the local population and natural environment of Greenland." We agree with the statement from Jørgensen et al. to the extent that meltwater itself is not a direct threat (i.e. Hg concentrations are far below national drinking water limits, as they are in all published studies of Hg in meltwaters). However, we disagree that there is a limited risk for populations and the environment, as this perspective does not account for the role of glacial meltwater as a vector of Hg to local oceans, and it is there that bioaccumulation and eventual exposure to humans and wildlife occurs.

While a portion of our dissolved Hg samples fall within the range of Jørgensen et al., similarity in concentration level alone isn't sufficient to support their broader risk assessment and requires context. As shown in our time series, dissolved Hg concentrations can range substantially with time of year, precipitation inputs, and meltwater volume. Monomethylmercury comprises a large majority of the dissolved Hg pool in the Kangerlussuaq catchment (e.g. up to 23 % MMHg), and particulate Hg is an additional, labile, and important Hg pool that is mobilized and transported from beneath the ice sheet to the downstream fjord system. The dataset presented by Jørgensen et al. is limited in temporal coverage and does not resolve Hg speciation (e.g. the quantification of methylmercury) or downstream Hg transformations from the meltwater river to coastal waters. Without these constraints, it is not possible to assess whether Hg is labile, bio-accessible, or present in its toxic and bioaccumulative form, nor is it possible to assess its risk as a toxic element to communities or ecosystems.

Our manuscript explicitly emphasizes temporal variability, dissolved and particulate Hg fluxes, and processes that can mobilize Hg from terrestrial systems to downstream fjords. We discuss Hg from the source to the sinks and provide additional dimensions that highlight pathways by which Hg can become bioavailable and ecologically relevant, even when dissolved Hg concentrations may appear low. Our focus is therefore on providing a process-based interpretation of Hg sources, grounded in our results, rather than endorsing or refuting previous statements.

In contrast, the MS includes an undebated use of [ref.11] to both frame the overall justification of the study (i.e. large differences in estimates of Hg yields from the GrIS) and implications of the study (discrepancies are a product of *difficulties in making accurate assessment*; please see above). Consequently, the current format of the MS makes the paper stand out as an indirect and non-convincing attempt to inform the scientific community that evidence supporting the conclusions of [ref.11] can no longer be produced without having to go into further details or discussions on why this could be the case and what the implications are.

In the current MS, the attempt to upscale Hg yields to the entire western margin of the GrIS (similar to [ref.11]) appears unconvincing for several reasons. One is that the upscaling exercise is based on data over a limited time period within a single calendar year (1 June to 21 July 2023) in which anomalous precipitation events were recorded. Another is that the data series going into the upscaling is based of a single sampling station at the very farthest location from the ice sheet itself (as compared to Ref. 11 and Ref.12) thereby having the largest potential input of non-GrIS derived Hg. The resulting Hg yield estimate stands out as a pure mathematical exercise with no auxiliary data supporting its validity, and thus very limited real-world relevance with an completely unconstrained uncertainty.

The reviewer suggests the approach to upscale Hg yields requires revisitation. Upscaling flux estimates from beyond a single catchment is commonly applied in biogeochemical assessments and literature. We agree with the Reviewer that it would be ideal to have measurements in several catchments of the GrIS to facilitate the analysis. The limitations of our upscaling, including the period limited to one year, is acknowledged in the manuscript and a conservative estimate on the flux is scaled for reference. However, the requested data is not feasible to collect or available from other sources. Therefore, our study provides the most accurate quantitation of Hg flux in a single catchment to date and scales the data using estimated discharge across catchments.

"Anomalous" precipitation events that the reviewer is referencing are becoming the new normal as the Arctic warms at a rate four times faster than the global average (Rantanen et al., 2022 and Saros et al., 2025 – see full citations below). To address the confusion here, the word "anomalous" was removed from the manuscript. See LN 92 for changes. The sampling station that the reviewer labels as "farthest from the ice sheet" is where the time series, daily sampling was conducted, approximately 25 km from the ice sheet margin. This is now clarified on LN 590-592. The reviewer suggests that by sampling here we have the largest potential input of non-GrIS derived Hg. In our manuscript, we show that early season Hg, particularly dissolved (filtered total Hg, fTHg) and monomethylmercury (MMHg) are likely to be sourced from proglacial sources in response to large rain events, and only when meltwater dominates do we suggest that Hg is of subglacial origin.

In addition to our daily sampling, spot samples collected spatially and temporally from each accessible subglacial-meltwater outlet in the Kangerlussuaq catchment were collected across the season, within 2 km of where meltwater rivers emerge from the ice sheet. This distance is now clarified on LN 610-614. As shown in the supplemental materials under the section titled "Synoptic Sampling", Hg concentrations remained consistent between meltwater glacial outlets at the margin of the glaciers (including Russell Glacier and Leverett Glacier) and the daily sampling site and were sometimes elevated at the closest-to-ice sheet site, indicating proglacial inputs were a minor source to the catchment during peak ablation season.

The sources of Hg to the catchment are discussed extensively throughout the manuscript. Our instantaneous flux estimates and our Hg stable isotope mass balance calculation show that a subglacial source of Hg is the predominate Hg source to the catchment. The upscaling calculation then provides a very conservative flux estimate of Hg in the region (~1 % of the current riverine Hg flux estimate to the Arctic Ocean), as a way for the reader to conceptualize Hg cycling in a broader context.

References:

(1) Saros, J. E. *et al.* Abrupt transformation of West Greenland lakes following compound climate extremes associated with atmospheric rivers. *Proc. Natl. Acad. Sci.* 122, e2413855122 (2025).
(2) Rantanen, M. *et al.* The Arctic has warmed nearly four times faster than the globe since 1979. *Commun. Earth Environ.* **3**, (2022).

* Is the work convincing, and if not, what further evidence would be required to strengthen the conclusions?

Overall, the sampling procedures and chemical analysis appear OK. Presented results on dissolved Hg for 2023 is of a similar magnitude as for 2021 and 2022 (see [ref.12]) and more than one order of magnitude lower than [ref.11].
As mentioned above, an important issue for other researchers in the community relates to obtaining an independent verification of the likely order of magnitude in Hg concentrations from the GrIS as the levels from [ref.11] (i.e.100-1000 times higher than what is known for freshwater systems in the arctic) and [ref.12] (i.e. similar range of dissolved Hg as presented in the current MS) cannot be easily reconciled. In the MS, this non-reconcilable issue is only vaguely touched upon, with no further discussion on the potential implications for the validity of the assertions and conclusions of [ref.11]:

Lines 46-48: "*However, the contribution of the GrIS to the Arctic Hg cycle remains highly uncertain due to large differences in estimated Hg yields (1,440–2,310 mmol km^{-2} year^{-1} versus 2.7-5.2 mmol km^{-2} year^{-1})11,12.*"

Lines 485-490: "*We estimate a southwestern GrIS total Hg yield of 23 mmol km-2 yr-1 (Table 1).* ***This yield is more than an order of magnitude lower than that reported by Hawkings et al.,*** *(2021), but comparable to data published from six major Arctic rivers (Table 1; 4 – 20 mmol km-2 yr-1)90, suggesting glaciers are a* ***non-trivial Hg source*** *to Arctic coastal waters. Discrepancies between studies underscore the challenges of accurately assessing Hg fluxes from dynamic glacierized catchments.*"

The fact that the concentration levels of the current MS most clearly supports the conclusions of [ref.12], in which the methodological deficiencies of [ref.11] was addressed, is conveyed only indirectly, with vague statements such as "…*cycle remains highly uncertain due to large differences*…" and "*Discrepancies between studies underscore the challenges of accurately assessing*…".

In my opinion, this is an unfortunate way of communicating the implications of these new results, which primary purpose should be to inform the scientific community that any signs of the previous assertions on extremely elevated Hg yields from the GrIS can no longer be detected in any of the sampling matrices investigated in the 2023 study when using appropriate and targeted sampling strategies and analysis.

We have added new text to the final section of the paper to clearly compare results of this study to previous studies. New text per the reviewer's feedback can now be found on LN 513-530.

3 – Other comments, recommendations and suggestions for improvement

1. I would encourage the authors to revise the MS to significantly enhance the discussion and implications of the above-mentioned main scientific knowledge gap related to the final settling of the claims of extremely elevated Hg yields from the GrIS [ref.11].

Per the reviewer's suggestions, new information regarding the discussion and implications of the previous work (Ref 11, Hawkings et al.) can now be found both in the introduction and in the discussion of the manuscript, see LN 46-51 and LN 520-527.

2. Aside the resolution of 1), the importance and relevance of the new data could be to provide additional site and process specific characterization of the chemical fingerprint of Hg in the Arctic environment at and around the GrIS to improve the understanding of how Hg is partitioned, potentially remobilized and/or transformed in the landscape. As presented in the 2021 AMAP Mercury Assessment, the dominating inputs of Hg to the Arctic is by long-range transport from regions outside of the Arctic. To investigate relative impact on Hg sourced from the subglacial domain, additional information on the distribution of Hg in the landscape and how this can potentially be remobilized. To achieve this, I would strongly recommend that the MS is revised with a focus on the best possible characterization and improved distinction of individual sources of Hg, for instance
   a. Hg originating in current long-range transport
   b. Hg originating in historical long-range transport (e.g. remobilization of historical long-range transport from soils, sediment, ice-melt others from the terrestrial and lacustrine parts of the landscape)
   c. Hg originating from current local/regional sources (e.g. subglacial geogenic Hg samples at the current location of the margin of the GrIS)
   d. Hg originating from historical local/regional sources (e.g. remobilization of geogenic Hg in the sedimentary archives in proglacial outwash plains and similar)

The reviewer asks that we distinguish among potential Hg sources (e.g. long-range transport vs local/subglacial inputs, historical vs contemporary pools). We agree that distinguishing Hg in

this catchment is critical to understanding how the Greenland Ice Sheet may contribute to broader Hg cycling, which is why we made this a central focus of our study. We address atmospheric inputs by directly sampling atmospheric Hg both through passive and active air samplers (see Supplemental Information). We then assess atmospheric inputs relative to terrestrial inputs using Hg stable isotope ratios. We demonstrate that our atmospheric Hg samples have a distinct isotopic composition from glacial meltwater. We show through an isotope mass balance mixing model that the glacial meltwater Hg pool has a large contribution from subglacial weathering rather than atmospheric transport, and only at peak ablation when supraglacial meltwater production is at its highest will Hg from atmospheric deposition comprise a significant proportion of the total Hg pool mobilized in the meltwater river (see Fig. 6 for isotope mixing approach). We further show that the isotopic signatures of meltwater are consistent with subglacial and geogenic sources, supporting a substantial contribution from local/regional Hg mobilized by subglacial weathering rather than atmospheric deposition alone. These results clearly provide the process-specific characterization and source distinction requested by the reviewer, and this was the aim of our research.

With respect to historical Hg, we agree that remobilization of Hg from permafrost and proglacial reserves is an important question. We did not obtain Hg stable isotope measurements from permafrost melt streams and a rigorous quantitative separation of these historical pools is beyond the scope of the present dataset. Nonetheless, our correlation analyses, statistical tests, Hg concentration time series, and instantaneous flux estimate collectively indicate that proglacial sources, such as permafrost, exert a measurable influence that is only detectable in the early season, before glacial meltwater discharge increases. At peak ablation, contributions from proglacial inputs appear to be low and are largely indistinguishable relative to the subglacial source signatures in the meltwater river, suggesting they currently represent a minor Hg source to the meltwater river at a seasonal scale.

These detailed characteristics of discrete sources could then related to and discussed in context of the mixed sampling environment in Kangerlussuaq:

e. Processes description of transformations processes, how these can be tested in the different reservoirs investigated (i.e supraglacial, subglacial, terrestrial, lacustrine and marine environments in addition to the atmosphere; see SI Fig 1 and 2)
f. What's the source specific signatures of Hg in the discrete reservoirs investigated (SI Fig 1 and 2)?
g. How do results from the above match with what you find in the mixed environment – both at different locations in the freshwater environment
    (different distances to the ice sheet with gradually increasing inputs from soils, sediment, groundwater and lakes) as well a along Søndre Strømfjord (four or five stations ? (Fig. 1A))
h. What's the likely relative importance of subglacial geogenic Hg to other known sources of Hg to the Arctic (including long-range transported Hg).

The reviewer suggests that the characteristics of discrete Hg sources be more explicitly related to the mixed sampling environment in Kangerlussuaq. Many of the requested elements are

already incorporated in the current manuscript. Specifically, we describe transformations and partitioning processes in the supraglacial, subglacial, terrestrial, marine, and atmospheric reservoirs using a combination of Hg concentrations, supporting geochemical analytes, and stable isotope compositions. We then use this data to interpret the composition of the mixed freshwater and fjord environments. We address the spatial evolution in the mixed system by comparing Hg concentrations, at multiple river sites located at increasing distances from the ice margin (LN 240-249), as well as along Kangerlussuaq Fjord (section title "Assessing the influence of ice sheet Hg on fjord surface waters"). This framework allowed us to evaluate if inputs from proglacial sources modify the glacial signal downstream, and to assess where and when non-glacial sources exert a detectable influence. We show that Hg concentrations from the glacial margin to the downstream river sampling site remain consistent, indicating proglacial sources have a minor contribution to the river catchment at peak ablation.

It is important to note that the river discharge reaches 1800 $m^3 s^{-1}$ at peak melt (see Fig 2), with turbid water moving rapidly from ice towards the fjord. This rapid movement of frigid, large volumes of water dilutes the minor proglacial sources and leaves little time for Hg transformations before discharging into fjord coastal zones. While we agree that Hg measurements from all individual end-members (e.g., permafrost and lakes) would further refine this picture, Hg isotope samples are difficult to collect, as each comprise a 30 L sample volume and a 10-day processing period in the field (as detailed in the manuscript and above). In short, these are non-trivial measurements. We chose to keep the sampling protocol for isotopes consistent to the meltwater river, as that was the focus of our study. The advantage of the Hg isotope approach is that it allows us to infer the relative contributions of different source pools without exhaustive sampling of every potential reservoir, by pulling on endmembers from literature, and the existing understanding of Hg isotope systems. Using the isotopic signatures of atmospheric, subglacial/geogenic, and downstream mixed waters, we can already constrain the likely relative importance of subglacial / geogenic Hg compared to other Arctic Hg sources and discuss these contributions in a process-based manner, which we executed extensively in the current manuscript (see section titled "Examining Hg provenance using stable isotopes").

3. The MS includes very extensive use of suggestive language (for example: *appeared to, is likely attributed, are best explained by, may relate to, etc.*) often unlinked to concrete observations. This can of course be an acceptable practice in certain cases and to some extent a matter of semantics, but excessive use of this type of argumentation structure in order to make things fit or to exaggerate the implications of results should be avoided. I recommend that the MS is revised for accuracy in this regard, particularly when describing the main claims of the paper, such as:
    a. L485: "..suggesting glaciers are a ***non-trivial Hg source*** to Arctic coastal waters"
    b. L496: "The GrIS is a ***considerable*** Hg source…"

    => For "non-trivial", "considerable" and similar wordings this to be truly meaningful, additional clarification/quantification/discussion need to be included with respect to the other known inputs of Hg to the Arctic ocean, especially the relative importance of 'naturally sourced Hg' (which is the main claim of the paper) ⇔ inputs of anthropogenic Hg (which is the dominant input based on AMAP and many other studies)

We have revised the presentation (see LN 532-539) to provide clarity on the differences between yields vs fluxes. Our findings show that, on an area basis, the Hg delivery from the GrIS is comparable to Arctic Rivers. But regarding the global flux, the GrIS is a small contributor (~1%), which is due to differences in discharge. We have clarified this indicates Hg from the GrIS is important locally to receiving coastal waters.

4. Since a main claim of the paper is that the Hg export from the GrIS and surrounding area could potentially increase with climate warming, concrete definitions of causal effects and process specific implications by "climate warming" need to be included in the MS. It is not sufficient to simply state the generic (and here unreferenced) statement that "the Arctic is warming nearly four times the global average" without explaining how and why this could have implications on the Hg cycle. Consider the following non-exhaustive list of examples could be the following:

a. L.23 "Early season permafrost melt" => this would likely relate to the seasonal freeze/thaw cycle of the active layer, unless the gradual expansion of the mazimum thaw depth is the target.

b. L. 31 "Climate-driven mass loss" => what is exactly meant by this and how does it relate to process specific Hg mobilization processes tested in the MS

c. L. 40 "Climatically sensitive Hg reservoirs" => unclear and needs further explanation

d. L. 125 " A permafrost melt stream…" => unclear and needs further explanation

e. L. 151 "Flushing of active layer and permafrost thaw…" => unclear and needs further explanation

f. L. 159 "active layer saturation in permafrost" => unclear and needs further explanation

The reviewer requests more information on causal effects of climate warming, particularly when discussing permafrost thaw. For clarity on this mechanism in regards to climate warming, more information has been added to the beginning of the discussion, now found on LN 131-135: "Previous research has shown that rising air temperatures increases soil temperatures, leading to deeper migration of the active layer depth and associated permafrost degradation[29]. Changes in the depth of the active layer perturbs soil hydrology, including soil water-retention capacity and hydrological connectivity, which can lead to surface ponding and runoff to downstream aquatic systems[30]."

In summary, I recommend that the MS is revised to very clearly communicate the implications of the current results in relation to what was previously proposed by parts of the co-authors group. Also, I recommend that the MS is revised to clearly define which hypothesis and scientific research questions can be tested by the data set and leave out all the suggestive explanations and vague interpretations which cannot be supported directly by the data itself. In its current form, the MS appears over-insistent on making observations from the main sampling location in Kangerlussuaq fit nicely together with the general hypothesis, i.e. that the GrIS is a significant source of Hg and thereby semi-confirming the claims of [ref.11] even though current concentrations are several orders of magnitude below.

We addressed this comment above in response to specific comments.

Instead, a focused process study clearly discussing the strengths and limitations of the study design (taking into account the limited time series and extreme weather conditions under which the study was conducted) could potentially improve our scientific understanding on the short and long-term sensitivity of various Hg stocks in the Arctic landscape. To achieve this, the MS would not need to include highly uncertain upscaling or claims of large scale acute global importance of its conclusions to be suitable for publication, but rather focus on what can actually be learned from the new data.

We addressed this comment above in response to specific comments.

In this revision process, I recommend that improved focus is made to clearly show and discuss also the data and results from the spot sampling of lakes, sediment and glacial streams. This would include also showing co-ordinates, sampling dates and other relevant information for these samples, including analytical results of field blanks. Of interest would also be a more clear discussion on the differences in the Hg signatures between the meltwater and particles of the supraglacial domain, meltwater and particles of the subglacial domain, freshwater bodies n the proglacial landscape, run-off from the terrestrial landscape as well as in the sedimentary archive in relevant landforms in the proglacial landscape between the GrIS and the ocean.

The reviewer requests information that was included in the original submission. Coordinates, sampling dates, and all geochemical concentration data including that for supplementary geochemical analytes are provided in the supplementary materials for all lakes, sediments, and glacial streams (Table S2). Analytical results of all field blanks are discussed in the methods sections and provided in the supporting data files (Supplementary Text 2, Table S9, and LN 640-646 of main manuscript).

In the revision, I recommend that the authors discuss the results and observed concentration levels of Hg in the MS against the findings of the recently published preprint by Olson et al (2026) containing a new database on mercury observations for permafrost regions. Here, published Hg data from lake sediment, vegetation, soil and water (lakes, rivers, creeks, oceans, wetlands, ponds and soil porewater) across the Arctic is summarized and presented – see example in summary table below. In comparison, the range of reported fHg/THg values of the MS (1.6-28.5 pM corresponding to 0,3-5,7 ng/L) show similarity in magnitude of concentrations of the studied proglacial watershed and meltwater runoff from the GrIS to the database's range for the 'water category' covering a wider range of environments and locations in permafrost regions in the Arctic and subarctic.

Table 1. Summary statistics of Hg observations in permafrost-dominated regions including lake sediment (ng g^{-1}), soil (ng g^{-1}), vegetation (ng g^{-1}), and water (ng L^{-1}). The minimum, 25th percentile, median, 75th percentile, maximum, geometric mean, standard deviation, and count are included for each medium type.

	Lake Sediment (ng g^{-1})	Soil (ng g^{-1})	Vegetation (ng g^{-1})	Water (ng L^{-1})
Minimum	1	1	1	0.0
25th Percentile	45	32	9	1.5
Median	70	50	15	2.5
75th Percentile	116	90	33	5.9
Maximum	9820	503	400	32.6
Geometric Mean	71	48	17	2.0
Standard Deviation	118	48	51	5.0
Count	113613	2923	590	676

Christine Olson, Kevin Schaefer, Alyssa Azaroff, HÃ©lÃ¨ne Angot, Tom Douglas, Maria Florencia Fahnestock, Charlotte Haugk, Gustaf Hugelius, Erfan Jahangir, Sofi Jonsson, Adam Kirkwood, Jennifer Korosi, Mina Nasr, David Olefeldt, Connor Olson, Laura Sereni, Sarah Shakil, Kyra St. Pierre, Lauren Thompson, and Scott Zolkos (2026) A Consolidated Database of Mercury Observations for Permafrost Regions. Earth System Science Data. https://doi.org/10.5194/essd-2025-640

We appreciate the reviewer bringing this to our attention. We have added a citation and comparison between our Hg concentration ranges that those reported of other water samples (see LN 523-530: "Notwithstanding, this study provides the best constraint thus far on particulate and filtered THg and MMHg concentrations from the GrIS across the melt season (1.6 – 17.5 pM fTHg, 0.1 – 2.3 pM MMHg, and 4.8 – 17 pM pTHg) which are within the range of other Arctic waters[101] and will help benchmark future Hg concentration measurements on the GrIS and other glacierized catchments. Changing hydrological and biogeochemical processes in glacierized catchments due to a warming climate could increase Hg fluxes to the Arctic Ocean by mobilizing Hg stored in ice melt, by accelerating subglacial weathering[102], or by proglacial sources, such as permafrost melt[99]."

Reviewer #2 (Remarks to the Author):

The topic and conclusions of this article are interesting, and this study aim to address a key issue worthy of investigation in the Arctic Hg cycling. However, the majority of the manuscript focuses on discussing the biogeochemical processes of mercury during glacial meltwater transport, while the sample collection and discussion of the to-ocean process are very limited. I understand this is hard to work in the Arctic regions. I still suggest to the authors should better align the main conclusion in more soft tune. The discussion of mercury concentrations is entirely isolated and disconnected from the discussion of mercury isotopes. Several conclusions drawn from the mercury concentration data may be not supported by the mercury isotope data. Therefore, the authors should reconcile the features from both mercury concentrations and mercury isotope data to derive robust and comprehensive conclusions.
The selection of the two end-members for the mixing model calculation is highly controversial. I do not agree with the authors' assumptions regarding these end-members. In fact, the best approach would be for the authors to measure the mercury isotopic signatures of supraglacial

meltwater and of subglacial meltwater immediately upon exiting the glacier. The authors need to verify these issues for the signatures of endmemeber. The transition from glacier to fjord lacks sufficient samples, particularly for mercury isotopes. I understand it is very hard to work at these stations. But I still worried about this issue that the revier spans a considerable distance (>100 km), meaning that about 90% of the purported "ice-to-ocean" pathway is missing from the analysis.

We thank the reviewer for their useful comments and the time they have taken to review our manuscript. We have taken the time to address their concerns (see below). We provided more context to our Hg stable isotope section in hopes to make this portion of the manuscript less isolated from the Hg concentration data. We also provided clarity in the Hg isotope section to ensure the conclusions align across the paper. We provided context to our selection of endmembers in the main text of the manuscript, including why we used the selected endmembers. We now clearly describe that previous literature on Hg stable isotopes from similar Arctic environments is very limited, and we therefore have carefully selected endmembers that best enhance and support the data we have observed. Per the reviewer's concerns, we have now also provided the methodology for our endmember calculation in our methods section. We have addressed their concerns on sampling location by providing more clarity within the manuscript and the methods section on sampling distance from the ice. We also added more information to our site description to show that concentrations between the ice margin and the primary sampling location are consistent (using synoptic sampling). See below for specific line numbers and modifications.

Line 51: How can glacial processes further mobilize Hg from atmospherically deposited Hg? Further explanation of the mechanism is needed.

Further clarification was added, LN 54-56: "During peak ice melt, glacial hydrological systems efficiently transport surface and subglacial meltwater and eroded subglacial bedrock to the ice margin[16,17]. These glacial processes mobilize and transport Hg from both atmospherically deposited and geogenic sources[18,19]."

Line 75: I need to know the distance between the spot samples on the glacier surface and plot 4, which appears to be roughly 150 km, as well as the types of soil and vegetation along this transect (e.g., wetland, tundra, grassland). This information is crucial.

Per the reviewer's request, more information on sample distance is now found in the methods section. See LN 590-592: "Daily water sampling (black square, Fig. 1) occurred between June 1 and July 21, 2023, at ~10:00 AM local time in the Qinnguata Kuussua (67.005°N, 50.684°W), approximately 25 km from the ice sheet margin."

In addition to the daily sampling, information on spot sample locations can be found in the Supplementary Material. We have added additional information in the synoptic sampling methods section. LN 612-613: "Glacial meltwater samples were collected within 2 km of each glacial margin."

Per the reviewer's request for more terrain depiction, more information was added to the site description in the methods section. Now on LN 574-576: "The Kangerlussuaq catchment from the ice margin to the fjord consists predominantly of glacial till terrain and low Arctic tundra, underlain by thin permafrost-affected soils developed on glacial sediments."

Line 106-117: The caption for Figure 2 is overly complex. It should be simplified, retaining only the essential information.

Figure 2 caption has been revised for clarity and flow. See LN 114-123.

Line 114-117: Is Filtered total Hg equivalent to Dissolved Hg (≤ 0.45 µm)? For subplot (d), which location in Figure 1 do the mercury concentrations correspond to? The time series location?

More information on filtered total Hg and how it is defined is provided in the methods section under "Riverine sample collection, processing, and storage." We explicitly use the term "filtered" due to recognition of the formation and prevalence of nano-particulate metal species (e.g., Aiken et al., 2011; DOI: 10.1021/es103992s). Filtered total Hg is defined as being filtered through a capsule 0.45 µm filter. We have revised LN 141-142 to clarify this: "…which supports that filter-passing Hg fractions are dissolved species." To address the confusion of sampling location, Fig 2 caption (LN 114) is revised to be: "Time series of hydrological and geochemical data in the Qinnguata Kuussua in 2023…"

Line 121-123: Which sampling site is being referred to?

To alleviate confusion, "catchment" was removed from this introductory sentence. LN 126-127 now reads: "High rainfall during the early sampling season (P1; Fig. 2a), led to saturated proglacial zones and appeared to enhance Hg mobilization into the Qinnguata Kuussua." The Qinnguata Kuussua sampling location is provided on the map in Figure 1, before this sentence.

Line 157-159: The explanation for the higher %MMHg is unconvincing and requires further elaboration. Also, explain why the %MMHg is significantly lower during the early period.

The reviewer claims percent methylmercury (% MMHg) is significantly lower during the early period. This is a misunderstanding, as % MMHg in the Qinnguata Kuussua averaged 23.3 % of the total filtered Hg (fTHg) pool in the early season. In comparison, late season % MMHg conditions comprise ~10 % of the fTHg pool. This indicates % MMHg is significantly higher during the early period, as discussed in depth in the manuscript and in this selected section (LN 163-181). Our assessment is the reviewer may have confused the differences between % MMHg and MMHg concentrations. To clarify this observation, we added additional text in LN 173-181 highlighting the importance of early season hydrology on MMHg production: "Early-season conditions of high precipitation coupled to low meltwater river discharge (~ 30 – 60 $m^3 s^{-1}$; Fig. 4) appear to have enhanced MMHg production and/or mobilization from permafrost into the Qinnguata Kuussua. However, we see a decline in % MMHg as discharge increases and at the onset of P1. While ambient concentrations of MMHg were elevated during P1 (2.2 pM) these

also corresponded to higher levels of fTHg (17.5 pM), causing a decline in the % MMHg ratio. Overall, we observed a distinctly higher release of MMHg in the early season when compared to the late season, demonstrating the importance of seasonality for MMHg production within this system."

Line 159-164: If, as the authors state, "MMHg showed a strong correlation to DOC in the early season," then why is the %MMHg extremely low during period P1 when DOC levels are high? The authors are advised to explain these processes from the perspective of mercury cycling mechanisms, rather than providing a mechanistic interpretation of correlations based solely on numbers.

As noted above, we believe there is confusion regarding the % MMHg interpretation. To properly assess % MMHg, the reviewer must consider the proportion of MMHg in the sample relative to the total fTHg fraction. During P1, there is a large spike in river DOC concentrations as well as a large spike in fTHg concentrations. Due to the rapid and sharp rise in fTHg concentrations during P1, the % MMHg slightly decreases (from 14% to 11%), as the total amount of fTHg in the river is much greater than before P1 (from 2.9 to 17.5 pM fTHg). During P1, MMHg concentrations rose to 2.2 pM, the highest concentrations observed throughout the time series. fTHg concentrations also rose to 17.5 pM at this time. Given these high concentrations of the dissolved Hg fraction, the lower % MMHg observed during P1 is a product of the high total fTHg concentrations during P1. We have now clarified this on LN 176-181.

Line 187-189: I do not fully agree with this statement. In my view, MMHg concentrations remain at relatively low levels throughout (with few exceptions). The authors should enhance the discussion from a geochemical perspective, rather than merely describing the data.

Previously reported Arctic environments have observed a % MMHg between 1 – 10 % in Arctic freshwaters. See citations provided below this response. Given the previously published data on % MMHg, our values in the early season (up to 23%) more than double that provided in previous literature of similar environment (Arctic rivers) and therefore indicate % MMHg is relatively high as a proportion of fTHg, particularly in the early season. We do not state that MMHg concentrations are elevated in the river water compared to other Arctic environments. The reviewer here suggests explaining these processes better, but we believe the reviewer is confused between the differences between % MMHg and MMHg concentrations. Extensive detail is provided on % MMHg in this section (LN 163-181) and correlations (LN 139-143) are used to support our conclusions. As mentioned in the above responses, LN 176-181 have now been added for clarification.

% MMHg in other Arctic freshwaters:

(1) Emmerton, C. A. *et al.* Mercury Export to the Arctic Ocean from the Mackenzie River, Canada. *Environ. Sci. Technol.* **47**, 7644–7654 (2013).
(2) Fitzgerald, W. F. & Lamborg, C. H. Geochemistry of Mercury in the Environment. in *Treatise on Geochemistry* 91–129 (Elsevier, 2014). doi:10.1016/B978-0-08-095975-7.00904-9.

Line 192-199: The authors' inference regarding the source of MMHg in the river water, attributing it to the glacier, is overly simplistic and unconvincing. Further evidence, such as from isotopes, is recommended. Processes like other surface runoff or subsurface seepage within the Qinnguata Kuussua catchment are more likely sources of MMHg.

The reviewer suggests our explanation for MMHg source is overly simplistic. However, we do not suggest that the source of MMHg in the meltwater river is primarily sourced from glaciers. We suggest that early season MMHg is likely to be coming from proglacial sources (i.e., permafrost; LN 128-131), and only in late season, when meltwater dominates, do we suggest that MMHg is coming from the subglacial environment. See LN 203-206: "This decline suggests that the dominant Hg source in the late season has lower MMHg concentrations than early-season, proglacial-dominated sources. However, the % MMHg of ~5 –10 % remains high compared to other Arctic environments[37,38], and late-season discharge (>100 $m^3 s^{-1}$) does not merely dilute a highly methylated Hg source." The % MMHg observed across the season is high, even in late season samples, compared to those observed in other Arctic environments. There is now evidence suggesting there are hypoxic or anoxic regions of subglacial environments where MMHg can be produced, which is what we suggest in our late-season samples (Lamarche-Gagnon et al., 2019 and Dieser et al., 2014 – see full citations below this response). MMHg accumulation in hypoxic subglacial environments occurs by anaerobic methylation of Hg(II). Information regarding subglacial Hg(II) methylation is now added to in this section of the manuscript on LN 219-222 for clarity.

Per the reviewer's concerns, we can use a simplistic calculation to assess seasonal proglacial vs. subglacial MMHg inputs. If the riverine MMHg was only supplied by seasonally persistent proglacial inputs (e.g. permafrost), we would expect a consistent MMHg instantaneous flux across the season. However, simple scaling of the average early-season MMHg instantaneous flux (18 nmol s^{-1}) to peak meltwater instantaneous flux (360 nmol s^{-1}), indicates that proglacial sources would account for only 5 % of the observed peak-melt MMHg load. Using the maximum MMHg concentration observed during P1 (2.2 pM, when we assume the highest proglacial inputs to the river), we estimate proglacial inputs would still only supply ~18 % of the peak-melt MMHg load, implying an additional MMHg source to the river is needed. This suggests the concentrations observed in the late-season samples are derived from an additional source, and we suggest that source is subglacial input. For clarification purposes, and to provide additional context to the reader, this scaling exercise is now added to the main manuscript text on LN 212-223.

Citations:

1) Lamarche-Gagnon, G. *et al.* Greenland melt drives continuous export of methane from the ice-sheet bed. *Nature* 565, 73–77 (2019).
2) Dieser, M. *et al.* Molecular and biogeochemical evidence for methane cycling beneath the western margin of the Greenland Ice Sheet. *The ISME Journal, 8* (11), 2305–2316 (2014).

Line 210-211: that exported pTHg is sourced primarily from subglacial physical weathering processes." Why? Provide more evidence.

The reviewer asks why pTHg is sourced from physical weathering processes. Glaciers are strong agents of physical erosion and glacial meltwater contains very high concentrations of suspended particulate material sourced from the ice bed (Overeem et al., 2017, Andresen et al., 2024 – see full citations below). pTHg has been found to be produced from subglacial erosion in previous literature (Nagorski et al., 2021 – full citation below), and extensive detail on pTHg production is provided throughout the manuscript discussion. Examples include:

1) The scaling of pTHg and SSC are linear, as described on LN 226-228: "pTHg concentrations across the season mimic SSC and discharge, with days 163, 174, 188, and 193 showing corresponding peaks in pTHg and SSC (Fig. 2b-d)."

2) Both SSC and pTHg concentrations increase with rising discharge, and the pTHg instantaneous flux drastically increases in the late season in response to rising subglacial discharge. Described on LN 231-235: "In the late season, including after and during P2, peaks in both SSC and pTHg corresponded with rapid discharge increases (Fig. 2c), suggesting that glacier hydrology and increasing glacial sediment flux enhance pTHg export (even as sediment-normalized concentrations decline), and that exported pTHg is sourced primarily from subglacial physical weathering processes."

3) Sequential sediment extractions point to reactive mineral surfaces and secondary weathering product sources, as discussed on LN 235-238: "A qualitative assessment of pTHg lability utilizing sequential particulate Hg extractions demonstrated that most pTHg resides in a labile KOH-extractable phase (87.3-96.7 % total extracted Hg; Fig. 2d), consistent with adsorption to reactive mineral surfaces and secondary weathering products generated at the ice-bed interface[50,51] (Supplementary Text 5)."

4) Synoptic samples collected at subglacial outlet portals have high pTHg concentrations that are similar to pTHg concentrations observed in late season samples, described on LN 243-246: "Synoptic samples from the Akuliarusiarsuup Kuua tributary, including Leverett and Russell subglacial outflows (Supplementary Fig. 1), reinforce the interpretation that pTHg in the Qinnguata Kuussua is of subglacial origin. Both glaciers had high pTHg concentrations (up to 22.2 pM), comparable to those observed in the late-season time series."

Citations:

1) Overeem, I., Hudson, B., Syvitski, J. *et al.* Substantial export of suspended sediment to the global oceans from glacial erosion in Greenland. *Nature Geosci* **10**, 859–863 (2017). https://doi.org/10.1038/ngeo3046
2) Andresen, C.S., Karlsson, N.B., Straneo, F. *et al.* Sediment discharge from Greenland's marine-terminating glaciers is linked with surface melt. *Nat Commun* **15**, 1332 (2024). https://doi.org/10.1038/s41467-024-45694-1
3) Nagorski, S. A., Vermilyea, A. W. & Lamborg, C. H. Mercury export from glacierized Alaskan watersheds as influenced by bedrock geology, watershed processes, and atmospheric deposition. *Geochim. Cosmochim. Acta* **304**, 32–49 (2021).

Line 257: In high-melt years, supraglacial meltwater will also increase, potentially at a rate exceeding that of subglacial meltwater. The authors should conduct a comprehensive discussion considering multiple factors, rather than focusing solely on the effects of increased subglacial meltwater. Therefore, Lines 259-266 should be reconsidered and rewritten to account for these various influences.

The reviewer suggests supraglacial meltwater will have an impact on MMHg and fTHg concentrations and encourages assessment of this source for the small late season flux denoted as P2, rather than only quantifying this source as subglacial input. Almost all supraglacial meltwater becomes subglacial as it passes through the subglacial drainage system before exiting the glacier (Chu, 2014 – see full citation below). Supraglacial meltwaters first drain to the subglacial bed, where they will inevitably interact with subglacial sediments and help evacuate subglacial meltwater at the glacier terminus. At peak melt, most of the water is supraglacial in origin. However, the supraglacial concentration of Hg will remain relatively consistent throughout the season because of its constant source (i.e. atmospheric deposition). Supraglacial samples were collected periodically throughout the season and showed the lowest fTHg concentrations than any other sample type (1.6 – 2.5 pM fTHg). The sample collected in late July (July 20th) had the lowest supraglacial fTHg concentration observed across the season (1.6 pM). The subglacial environment responds to increased surface melt, as discussed in detail through LN 259-262: "Subglacial drainage evolution is intricately connected to supraglacial meltwater production[47,48,52]. Subglacial channels develop into efficient conduits that rapidly transport meltwater and eroded subglacial bedrock (including overridden paleosols) to the ice margin in response to increased supraglacial meltwater input[16,17,53]." As well as on LN 281-282: "In high-melt years, the snowline tends to recede to higher elevations, which may increase meltwater transport capacity to the glacial bed further inland[16]." This process flushes out subglacial waters with residence times that could comprise up to several years or more – the supraglacial meltwater is therefore critical in driving hydrology and could become even more important as snowline recede deeper into the ice sheet interior. Although supraglacial meltwater volumes are very high in the late season, the concentrations of Hg in supraglacial meltwater remain low, and Hg fluxes therefore appear to be driven by glacier hydrology and the mixing of subglacial waters.

The reviewer suggests we conduct a comprehensive discussion on supraglacial vs subglacial meltwater impacts for Hg. We do so in our isotope mass balance equation, found under the section titled "Photochemical transformations in supraglacial meltwater" (LN 351). In this mass balance, we show that ~50% of the Hg could be coming from supraglacial sources at peak melt, but the remaining 50% is still comprised of subglacial and/or proglacial Hg sources.

Citations:

1) Chu, V. W. (2014). Greenland ice sheet hydrology: A review: A review. *Progress in Physical Geography: Earth and Environment*, *38*(1), 19-54.

Line 275-278: This paragraph does not convey any original viewpoint from the authors in this context. It is recommended for deletion or relocation to the Introduction.

Given the size of the dataset and information covered in the manuscript, we opted to keep the statement pertaining to mass dependent and mass independent fractionation in this section for the ease of the reader. The discussion of previously measured isotope values is best suited for a discussion alongside the data, but we have streamlined the text so that it directly related to the system and does not read as an introductory paragraph. Please see LN 299-306 for the changes.

Line 289: This statement is overly absolute.

We have clarified by moving the potential of MIF earlier in the discussion. We note that MIF processes contribute to changes in fTHg isotopes (as observed in days 185-190, LN 318-321), but photochemical fractionation cannot explain the δ^{202}Hg increase observed in days 150-180. The language of this paragraph was changed to "We attribute the increase in δ^{202}Hg across sampling days 150-180 to geogenic rock weathering…" (LN 313-314) to emphasize that this is our best hypothesis to explain the independent shift MDF. We further acknowledge that processes like methylation and de-methylation can alter Hg isotope values but given that MMHg is a small portion of the overall total Hg budget we do not expect it to greatly modify MDF values observed in fTHg.

Line 290-298: As the authors themselves state, MDF could occur due to redox transformations, volatilization, kinetic, and biological reactions. Therefore, they should not use MDF signatures for qualitative source apportionment, as this is inaccurate and one-sided.

As noted by the reviewer, it is correct that MDF can occur due to redox transformations, volatilization, kinetic, and biological reactions. Therefore, it is correct that used on its own, MDF may not uniquely tell you about source apportionment. However, it can provide useful qualitative details such as observed source or process changes as discussed in LN 299-339 of the manuscript. The data description and inferences made from such align with other Hg isotope literature which describes different isotope distributions in similar manners (see references 63-70 for examples). Lastly the reviewer's assertion that source apportionment is inaccurate using MDF is not correct, no source appointment was performed using MDF. Rather, we used MIF (Δ^{199}Hg) as a conservative tracer to estimate potential source contributions from weathering versus geogenic origin. To ensure no confusion on the part of the reader, we have changed the section header to "Examining Hg provenance using stable isotopes".

Line 300-301: This statement is inconsistent with the results shown in Figure 6A.

This statement was rephrased to the following for clarification, LN 323-324: "Values of δ^{202}Hg for particulates collected in the Qinnguata Kuussua overlap with the previously published range associated with Precambrian metamorphic and sedimentary bedrock[69]."

Line 303-304: This statement contains a clear error. There have been some reports on mercury isotope measurements in glaciers.

We thank the reviewer for pointing this out. The reviewer is correct; there are limited isotope studies in glacial watersheds, but there have been some reports. We have added references to two additional papers that examine glacial meltwater and ice cores. These references are attached

below this response and included in the main text of the manuscript. We now also refer to these in the text on LN 383-386: "A lower than expected odd-MIF suggests that the source of fTHg in the Qinnguata Kuussua is a mix of surface and subsurface glacially-derived Hg similar to those observed in the Urumqi Glacier in China and ice cores from the Canadian Arctic[82,83]."

Citations:

1) Zdanowicz, C. M. *et al.* Historical variations of mercury stable isotope ratios in Arctic glacier firn and ice cores. *Glob. Biogeochem. Cycles* **30**, 1324–1347 (2016).
2) Liu, N. *et al.* Mercury Transport, Transformation and Accumulation Recorded by Stable Isotopes during Retreated Glacier Chronosequence of 250 Years. *Environ. Sci. Technol.* **59**, 6085–6096 (2025).

Line 319: I do not understand why the authors cite the mercury isotopic signatures of pre-Cambrian rock and Hg(II) in mid-latitude snow. In my opinion, these are clearly unrelated to mercury sources in glacial-river water. The selection of end-members is critical, and the authors should be more careful.

These end members were chosen due to the limited data available for Hg isotopes measurements. First, our pTHg samples are compared to previously published Precambrian rock (Deng et al., 2022) because there are no Hg stable isotope ratios for Greenlandic bedrock published, and this rock type is the same geology that underlies our sampling location (Fig 1). More detail on this rock type for clarity is now added in the manuscript on LN 323-324.

Per the reviewer's concern on endmembers, more information can now be found in an additional methods section titled "Endmember selection for stable isotope mixing model". In this section we now denote (LN 752-764): "Data on supraglacial sources of Hg were highly limited in the literature. The geographically closest candidate for endmember samples were one snow measurement from Greenland (Δ^{199}Hg = 0.32 ‰)[84] as well as firn and ice core samples from the Canadian Arctic from post-industrial (Δ^{199}Hg = 0.08 ± 0.28 ‰) and pre-industrial (Δ^{199}Hg = 0.28 ± 0.83 ‰) periods[82]. However, measurements of Δ^{199}Hg in fTHg from Qinnguata Kuussua greatly exceed these values, particularly in late season, thus these published values cannot be used as endmembers. Instead, we opted for Δ^{199}Hg values in snow as a proxy, but were limited in selection of published values because we saw no isotopic evidence of atmospheric Hg depletion events in our measurements, unlike the highly negative MIF values previously reported in Arctic snow[87]. Due to these limitations in endmember designation, we selected a mid-latitude snow Δ^{199}Hg value (3.5 ‰)[73]. These values were similar, albeit lower, when compared to glacial surface ice samples measured in the Tibetan plateau[68], but represented a more conservative value for mixing model calculations."

We also have added additional information on the Tibetan Plateau samples (He et al. 2025) and their Δ^{199}Hg values, now on LN 364-370: "This process produces large, positive Δ^{199}Hg fractionations, with previous literature reporting up to 3.50 ‰ in mid-latitude snow collected in Michigan, USA[73] and up to 4.06 ‰ in glacial surface ice samples collected in the glacierized Tibetan Plateau[68]. As surface meltwater production intensifies over the season, a larger fraction of meltwater is sourced from the illuminated ice surface rather than basal melt or stored

waters[77,78]. This is likely to lead to increasingly more positive Δ^{199}Hg values (Fig. 6b), as a large proportion of deposited Hg could be rapidly re-emitted to the atmosphere[68]."

Line 332-334: Additional references should be included.

It is unclear what additional references should be included pertaining to pTHg measurements. There are other studies that measure suspended particulate matter in the water column, but these often focus on contaminated sites (Baptista-Salazar et al. 2018, Washburn et al. 2017, Washburn et al. 2018, Janssen et al. 2021, and Eckley et al. 2024) and are not applicable to this study. There was also recent work on suspended particulate matter and its role in sedimentation in coastal regions (Liu et al. 2025), but this was predominantly inferred through sediment core records. The Baldwin and Campeau papers are the only ones, to our knowledge, that conduct Hg isotope values on pTHg across a watershed-scale in generally undeveloped or geogenic diverse regions. If there are additional references that we have missed please let us know.

Line 352: A range of -0.43 to -0.07 is very large. The authors' explanation does not support their conclusion that "This supports the interpretation that pTHg originates from bedrock weathering."

The reviewer suggests the range in Δ^{199}Hg (-0.43 ‰ to -0.07 ‰) is very large. This portion of the manuscript has now been revised per the reviewer's feedback. See LN 373-377. Importantly, the total observed range in the Hg isotopic composition of natural materials is >10 ‰ for Δ^{199}Hg and >8 ‰ for δ^{202}Hg (Blum et al., 2014 – see full citation below). Given the large range in isotopic composition of natural materials and noting our analytical precision ± 0.06 ‰ Δ^{199}Hg (see table S5 for more information on precision and CRMS), our range of pTHg values are not unusual.

Citations:

1) Blum, J. D., L. S. Sherman, and M. W. Johnson. 2014. Mercury isotopes in Earth and Environmental Sciences. *Annual Review of Earth and Planetary Sciences* 42: 249–269. https://doi.org/10.1146/annurev-earth-050212-124107.

Line 358: This assumption by the authors is far-fetched. I do not fully agree with the assumption that supraglacial GrIS meltwater can reach a δ202Hg value of 3.5‰. I recommend that the authors add actual measured data for this. I note that in Figure S1, the mercury concentration in the supraglacial sample is not very low, especially for fTHg, which suggests the proportion of photochemical reduction might not be high. The authors should consider this comprehensively.

The reviewer claims that supraglacial GrIS meltwater cannot reach a δ^{202}Hg value of 3.50 ‰. We think the reviewer is confused here and may have misunderstood the data. This is incorrect information that we do not include in our manuscript. Our manuscript states on LN 379-381: "Given the intense ultraviolet (UV) irradiation experienced in supraglacial environments, we would anticipate supraglacial GrIS meltwater to exhibit pronounced positive odd-MIF signatures (i.e., >3.50 ‰ Δ^{199}Hg)[73], although never measured before." We do not include mass-dependent fractionation (i.e. MDF, δ^{202}Hg per the reviewer comments) for assessment of supraglacial characteristics in our watershed. We instead use mass-independent fractionation of Δ^{199}Hg, which is used in previous literature as a photochemical tracer for Hg (examples: Kurz et al., 2021

and He et al., 2025 – see full citations below). Positive $\Delta^{199}Hg$ has been observed both in mid-latitude snow and in glacial ice core samples and attributed to high photochemical reduction in luminescent surface environments. Supraglacial fTHg samples collected in this study range from 1.6 – 2.5 pM and are the lowest fTHg concentrations found from all synoptic samples collected throughout the catchment (LN 253-256). Given this, combined with previous literature on photochemical reduction in surface environments, we believe using $\Delta^{199}Hg$ (not $\delta^{202}Hg$) is a suitable way to trace Hg exposed to high photochemical reduction.

Citations:

1) Kurz, A. Y., Blum, J. D., Johnson, M. W., Nadelhoffer, K. & Zak, D. R. Isotopic composition of mercury deposited via snow into mid-latitude ecosystems. *Sci. Total Environ.* **784**, 147252 (2021).
2) He, S. *et al.* Stable Isotopes Reveal the Contribution of Glacier Melting to Mercury Budget in Tibetan Rivers. *Environ. Sci. Technol. Lett.* **12**, 85–91 (2025).

Line 371: I do not agree with the two end-member characteristics assumed by the authors. It is recommended to select appropriate end-members and rewrite the entire mercury isotope discussion accordingly. Also, there is a clear error in this formula; the denominator should not be pTHg, but rather the signature of the subglacial meltwater end-member.

First, to clarify the confusion regarding the calculation we have simplified the abbreviations from "pTHg" to "sub" and "mid-latitude" to "snow". The calculation and endmembers have stayed the same, but the nomenclature was changed to avoid unnecessary confusion. (LN 394).

Second, the reviewer is incorrect in their assertion that MDF is being used to estimate source attribution (see responses to comments above). Here we are using MIF, specifically $\Delta^{199}Hg$, which has been used in similar systems to quantify Hg sources (see Liu et al. 2025, https://doi.org/10.1021/acs.est.4c13057). The $\Delta^{199}Hg$ value is considered a conservative tracer because it is predominantly altered by photochemical reactions and not "dark reactions" (i.e., the absence of sunlight, see references 61, 68 and 73), in this case mineral weathering.

Third, we understand that the reviewer is frustrated with our selection of end members, but the collection of these samples during the field campaign was not possible due to logistical constraints. We agree that it would be best to get bedrock from Greenland and direct ice melt, but we do not have this data and need to use reference data in the literature for this estimate. We have added a sentence acknowledging that additional endmember characterization is needed in future studies of the GrIS on LN 415-417: "To better constrain subglacial and supraglacial sources from the GrIS, future studies should consider the collection of additional endmembers including snow, ice, permafrost, and geogenic material."

Fourth, we have added more information regarding end-member selection to the methods section. See LN 746-764: "To estimate the contribution of Hg from subglacial sources, a binary mixing model using $\Delta^{199}Hg$ was employed, similar to the approach used in other glaciated systems[83]. Endmembers of weathered rock and supraglacial meltwaters were not available due to sampling constraints and lack of previous measurements, so proxy values were used. In the case

of subglacial $\Delta^{199}Hg$ values, we used average Precambrian bedrock values from the literature (average $\Delta^{199}Hg$ = -0.01 ‰ ± 0.16, Table S3), which were the same age and rock type as our sampling location in Greenland. Data on supraglacial sources of Hg were highly limited in the literature. The geographically closest candidate for endmember samples were one snow measurement from Greenland ($\Delta^{199}Hg$ = 0.32 ‰)[84] as well as firn and ice core samples from the Canadian Arctic from post-industrial ($\Delta^{199}Hg$ = 0.08 ± 0.28 ‰) and pre-industrial ($\Delta^{199}Hg$ = 0.28 ± 0.83 ‰) periods[82]. However, measurements of $\Delta^{199}Hg$ in fTHg from Qinnguata Kuussua greatly exceed these values, particularly in late season, thus these published values cannot be used as endmembers. Instead, we opted for $\Delta^{199}Hg$ values in snow as a proxy, but were limited in selection of published values because we saw no isotopic evidence of atmospheric Hg depletion events in our measurements, unlike the highly negative MIF values previously reported in Arctic snow[87]. Due to these limitations in endmember designation, we selected a mid-latitude snow $\Delta^{199}Hg$ value (3.5 ‰)[73]. These values were similar, albeit lower, when compared to glacial surface ice samples measured in the Tibetan plateau[68], but represented a more conservative value for mixing model calculations."

Line 403-420: This conclusion is absurd. I find it completely incomprehensible.

We have revisited the conclusions and deemed the original presentation to be comprehensible. Unfortunately, the reviewer provided no context or insight regarding changes needed for this section. Minor clarifications were made to this section of text, but the literature cited supports the conclusions drawn from the data. We added a caveat regarding the need for additional research as well.

Reviewer #3 (Remarks to the Author):

This manuscript is a large study on the mobility and transformations of Hg in the southern region of the Greenland Ice sheet. The study shows that Hg is released both from weathering under the glacier and from the glacial meltwater and that Hg is released into the marine fjord over the course of the northern summer season. This is a novel study using both traditional biogeochemical (measurement of Hg concentrations and discharge allowing a mass balance analysis) and newer, advanced stable isotope approaches. This will be of great interest to those in the field and a wider audience, especially given the recent focus on the Arctic under a warming climate. The conclusions are original. The methods are appropriate and convincing with no further evidence required to support the authors' conclusions. My main suggestions are for increased clarity around the definition of melt timing and details on speculated processes on how MMHg is produced in the fjord. Please see below for detailed comments.

We thank the reviewer for taking the time to read and provide constructive comments on our manuscript. The revisions provided by this reviewer were helpful for enhancing the quality of our manuscript. Per the reviewers concerns we have now provided clarity on the definition of melt timing and details on how MMHg is produced in the fjord. See more details on these additions and specific line numbers below.

Line 61 and 64. It appears that the river's name is presented as the Indigenous name, with the colonizers name in brackets in line 61. In line 64 (and throughout the manuscript), it appears the

colonizer's name for the Fjord is listed first. If this is the case, I suggest that Greenlandic names are used as a priority over colonizer's names.

As per the reviewer's request, Greenlandic names are now used as the preferred names throughout the manuscript.

Fig 1. Defines blue areas as Ocean. However, it appears that blue areas also include lakes and rivers. Change the legend to reflect all water-bodies.

As per the reviewer's request, the legend in Figure 1 has been updated to reflect all water bodies.

Fig 2 panels are out of order. Fig 2d (line 174) is presented prior to 2c. (line 204)

Per the reviewer's suggestion, Figure 2 panels are now re-ordered. See Fig 2 on LN 114.

More clarity is needed on what is happening early vs. late with respect to melt prior to mention in the Fig. 5 caption. The discharge data suggest that melt water started to be measured around day 175, approx. 10 days after the definition of late at about day 163. Why is "late" defined as day 163?

To provide clarity to the reviewer's comments on early vs late season, an additional sentence was added to the manuscript. Now starting on LN 108-112 as follows: "For this study, we define "early" season (days 152 – 164) when discharge remains low (<100 $m^3 s^{-1}$), SPC is relatively high, and when the relative contribution of terrestrial runoff is higher. A subsequent "late" season (days 165 – 202) is defined by the onset of sustained discharge increase (>100 $m^3 s^{-1}$), and concurrent SPC dilution, indicating the progressive transition to rising supraglacial melt contributions."

Fig. 6. Gorgeous figure and this is really minor, but it seems more intuitive to order the colours from light to dark over the season.

We thank the reviewer for this kind comment. The reviewer suggests ordering the colors from light to dark instead of from dark to light in Figure 6. While we also agree it is more intuitive to order the colors from light to dark, the reason for not doing so is due to sample distinction. If the gradient was organized from light to dark per the reviewer's request, then it would become increasingly harder to distinguish between late-season fTHg and pTHg sample types. See the examples below:

Example 1: Shows the samples organized light to dark per the reviewer's request. Note that the fTHg (blue circles) and pTHg (red circles) become increasingly hard to distinguish in the late season (late season = darker colors in this example).

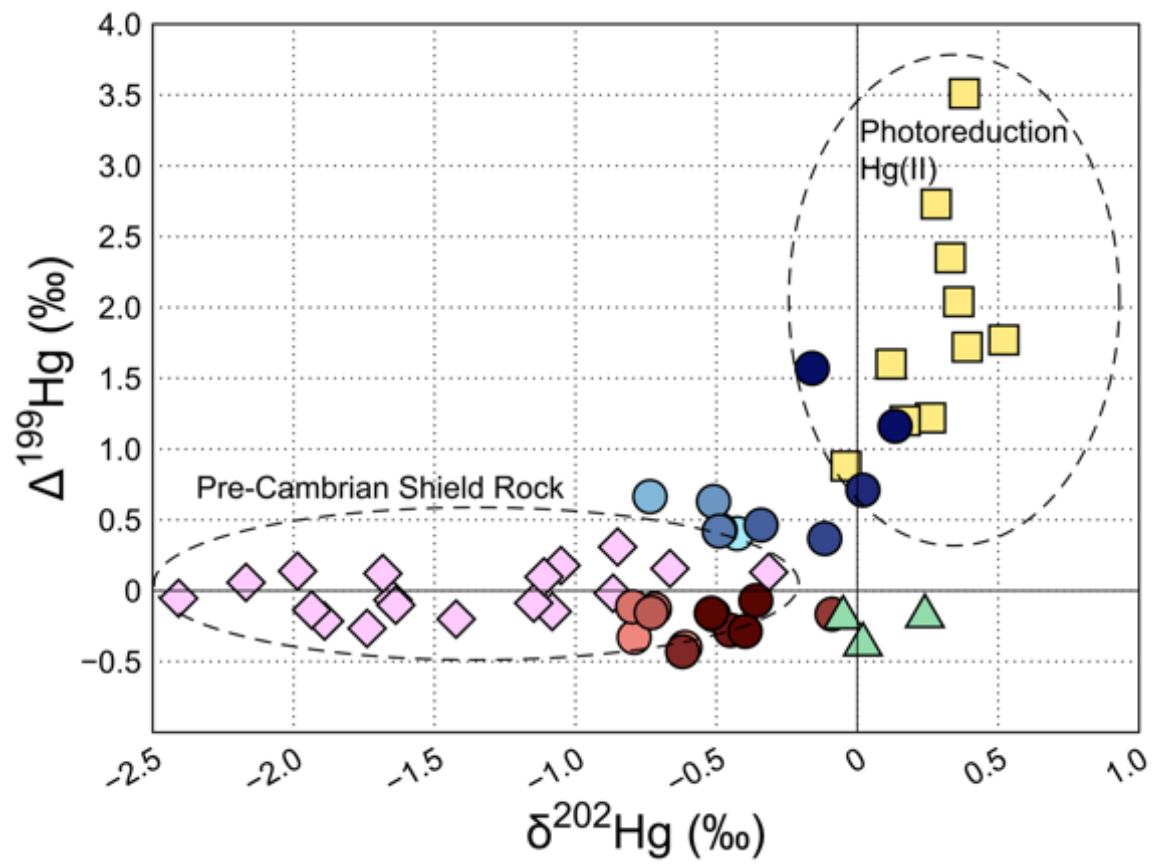

Example 2: Preferred presentation of the figure, with gradients dark to light. Late-season samples are more easily distinguishable.

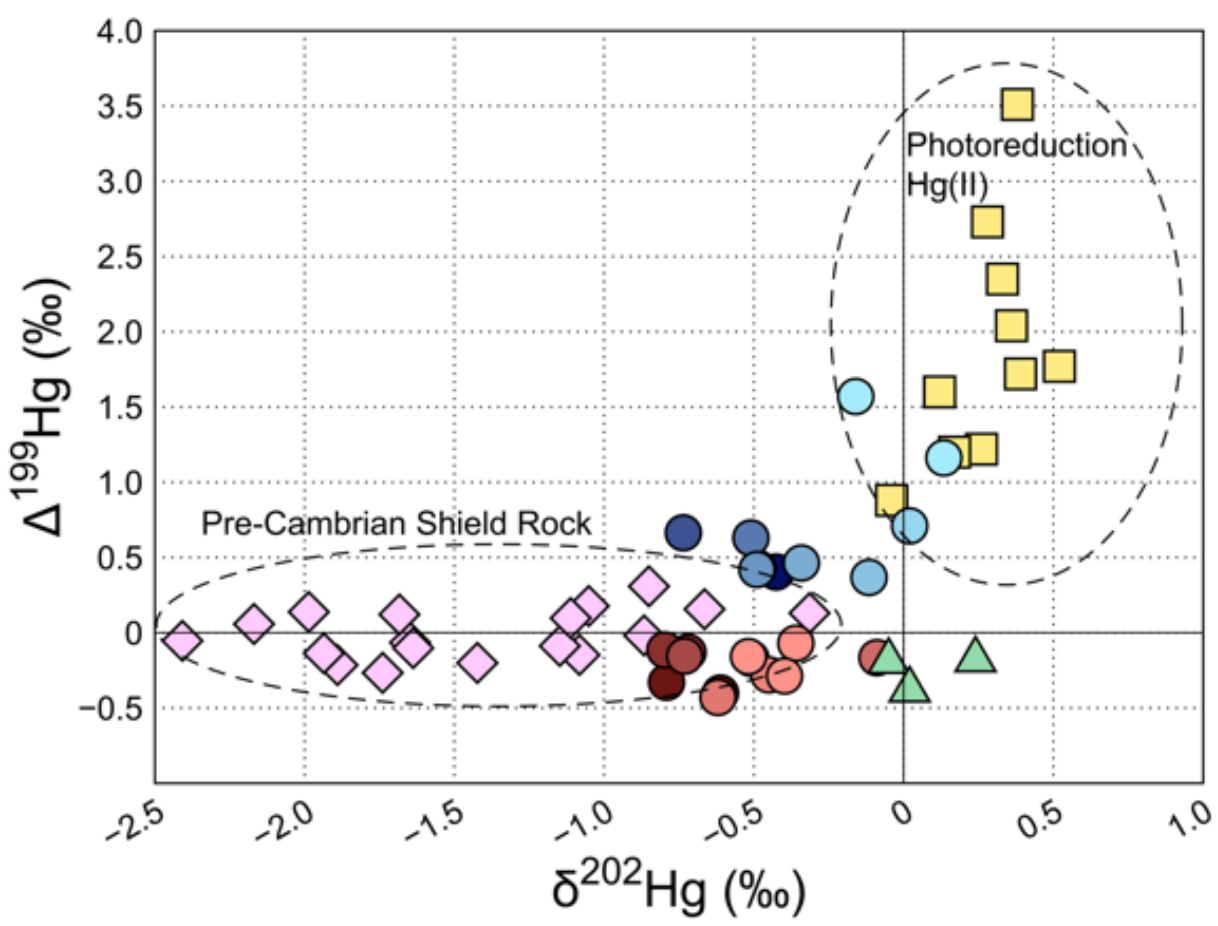

Line 455. I am not clear on the process by which fHgII and pHgII undergo desorption and are converted to MMHg. More clarity on this would be helpful. Are authors suggesting abiotic methylation? How?

We clarified the statement and added appropriate references of studies documenting the methylation of filtered Hg(II) within the water column of marine systems. See LN 485-488.

Table 1. Please define uTHg in the table. It is not defined until line 553 in the text.

Per the reviewer's request, uTHg is now defined in Table 1 caption as follows on LN 541-542: "Measured discharge weighted mean Hg concentrations, estimated fluxes, and estimated yields of filtered total Hg (fTHg) and unfiltered total Hg (uTHg)."

Line 566. Better define and clarify "synoptic samples". I do not know what this means.

To address reviewer's comment on synoptic samples, more explanation was added. Now on LN 610-612: "Synoptic samples for total Hg (uTHg and fTHg) were also taken around the Qinnguata

Kuussua catchment to assess the Hg composition of endmembers. Synoptic samples include glacial meltwater, proglacial lakes, and a permafrost melt stream (Table S2)."

Line 578. Clarify "… filters used for sample collection were pumped dry…" I do not know what this means.

To address the reviewer's comment, revisions were made as follows now on LN 625-627: "The capsule filters used for sample collection were pumped dry (i.e. all remaining water was evacuated from inside the capsule) and stored frozen to be digested for suspended particulate Hg isotope measurements."

Line 584. Define FEP

Per reviewers' suggestion FEP is now defined as follows on LN 632.

Line 604 – remove "from" or "using"

Per the reviewer's suggestion "using" was removed. Now on LN 655-656: "Fjord samples for Hg concentration were collected in early July from the Greenlandic vessel M/V Tulu."

[Journal assessment criteria marked in ‘***blue**. Review comments marked in **black.]**

***Criteria for publication**

*To be published in *Communications Earth & Environment* a paper needs to meet several general criteria:

- The conclusions are **novel** (novelty is not compromised by either abstracts or internet preprints)
- The paper provides **strong evidence** for the main conclusions
- The underlying data are generated and processed at a state-of-the-art level, including **tracking of uncertainties**
- The manuscript is **important to scientists in a specific sub-field** of the Earth, environmental or planetary sciences.

*In general, to be acceptable, a paper should meet these criteria and represent an advance in understanding that is expected to influence thinking in the field.

---

*When composing your report, the following questions might assist you in writing a helpful, well-justified review.

*We would here like to share a few tips on writing a report, and outline what we value most in the reports we receive.

1. *Start with a brief summary*: An outline of the gist of a paper you are reviewing can be very helpful with clarifying in your own mind the authors' line of argument. Your summary also gives authors a different perspective on their own work, which can help define their focus, as well as a chance to discover, and then counter, any misunderstandings that may have occurred.

2. *Back up your assessments*: Where you think the presented work is not novel, it helps editors and authors if you provide references where similar findings have been reported previously. If you have technical concerns, spell out where exactly you think the flaws lie, and what you think has been missed. The authors may be able to address these concerns—but only if they understand them.

3. *Keep the tone scholarly, encouraging, and positive*: Peer review is first and foremost intended to help improve the scientific literature. That is best done by keeping your comments dispassionate1. Confrontation, emotive language, or sarcasm are unhelpful; instead, choose a tone you would use with a friend (whether or not you know the authors).

4. *Consider and comment on each claim*: Most papers present more than one conclusion. If parts of the paper don't hold up, perhaps others do? As editors, we have the task to balance our editorial requirements for novelty and importance of a piece of work with the comments from a panel of reviewers with different perspectives. With your advice on which claims by the authors are compelling, we can decide whether these claims advance science sufficiently to fit the journal's aims.

5. *Be generous with ideas for improvement—but do not insist*: No paper is perfect, and there is always more that could be done. So if you have suggestions for how the authors could improve their paper, do share them—but do not expect or demand that every idea will be implemented. It is the authors' paper after all.

6. *Note where you cannot follow*: Most likely, as a reviewer you know more about the subject area than many other readers. So, if you don't understand, others will run into the same difficulty. Outline what you find confusing. Ask the authors to explain what they mean where the writing is unclear, in particular, where it's central to the conclusions.

7. *Be open about the limits of your expertise*: If only part of the manuscript falls within your specialty, let the authors and editors know. It is not always obvious to editors what exactly a prospective reviewer has direct knowledge of, but where we know, we will weight comments accordingly.

8. *Do not request citations to your own group's papers, unless essential*: Because you will be most familiar with your own work, it is easy to rely on it in your review beyond its fair share of the literature. Try to counterbalance that tendency by carefully considering what else has been published. Only ask for your own articles to be cited if they are obviously key to the story. In those cases, ideally explain in the notes to the editors why you think this is the case.

9. *Judge the science, not the scientist*: We strongly recommend that you disregard what you know about the authors. Where they chose double-blind peer review, we suggest that you do not dwell on guessing their identities, and certainly that you do not hold that against the authors. What matters should be the quality of the research, and not who wrote it or which research institution it came from.

---

1 - Brief summary

* What are the major claims of the paper?

The study set out to characterize Hg sources, transport and export in meltwater from the Greenland Ice Sheet (GrIS) using a combination of repeated daily sampling of meltwater samples at a sampling station in Kangerlussaq, West Greenland, spot samples at a number of meltwater rivers, spot samples of glacier surface, spots samples of atmospheric deposition and marine samples in a transect along Søndre Strømfjord.

The main claim of the manuscript (MS) is that high temporal variability in Hg concentrations in glacial meltwater over the period 1 June 2023 to 21 July 2023 can be linked to the evolution of the Greenland Ice Sheet (GrIS) hydrological system and relative inputs of supraglacial and subglacial meltwater (ll 509-511), indicating a seasonal shift in the source of Hg from early melt season inputs of Hg from the terrestrial environment to a more pure subglacial sources at a more mature stage of the melt season.

Data and results of the MS share similar characteristics with two other recent studies conducted in the same study area in Western Greenland (Hawkings *et al.*, 2021 [ref.11 in the MS]; Jørgensen *et al.*, 2024 [ref.12 in the MS]) although the reported types of samples are expanded with both stable Hg isotopes and air samples.

Ref.11: Hawkings, J. R. et al. Large subglacial source of mercury from the southwestern margin of the Greenland Ice Sheet. Nat. Geosci. 14, 496–502 (2021).

Ref.12: Jørgensen, C. J. et al. Large mercury release from the Greenland Ice Sheet invalidated. Sci. Adv. 10, eadi7760 (2024).

Based on mean concentrations from the main sampling station in Kangerlussuaq (see picture from Google Earth below), a total Hg yield estimate for the southwestern margin of the GrIS is calculated based on simple multiplicational upscaling.

The relative contribution of geogenic and atmospheric sources contributions are investigated using analysis of Hg stable isotopes showing indications that subglacial weathering of bedrock could be a potential source of Hg. The MS concludes that the GrIS is a considerable Hg source and that climate-warming will likely increase the contribution of ice sheet derived Hg to the Arctic Hg budget.

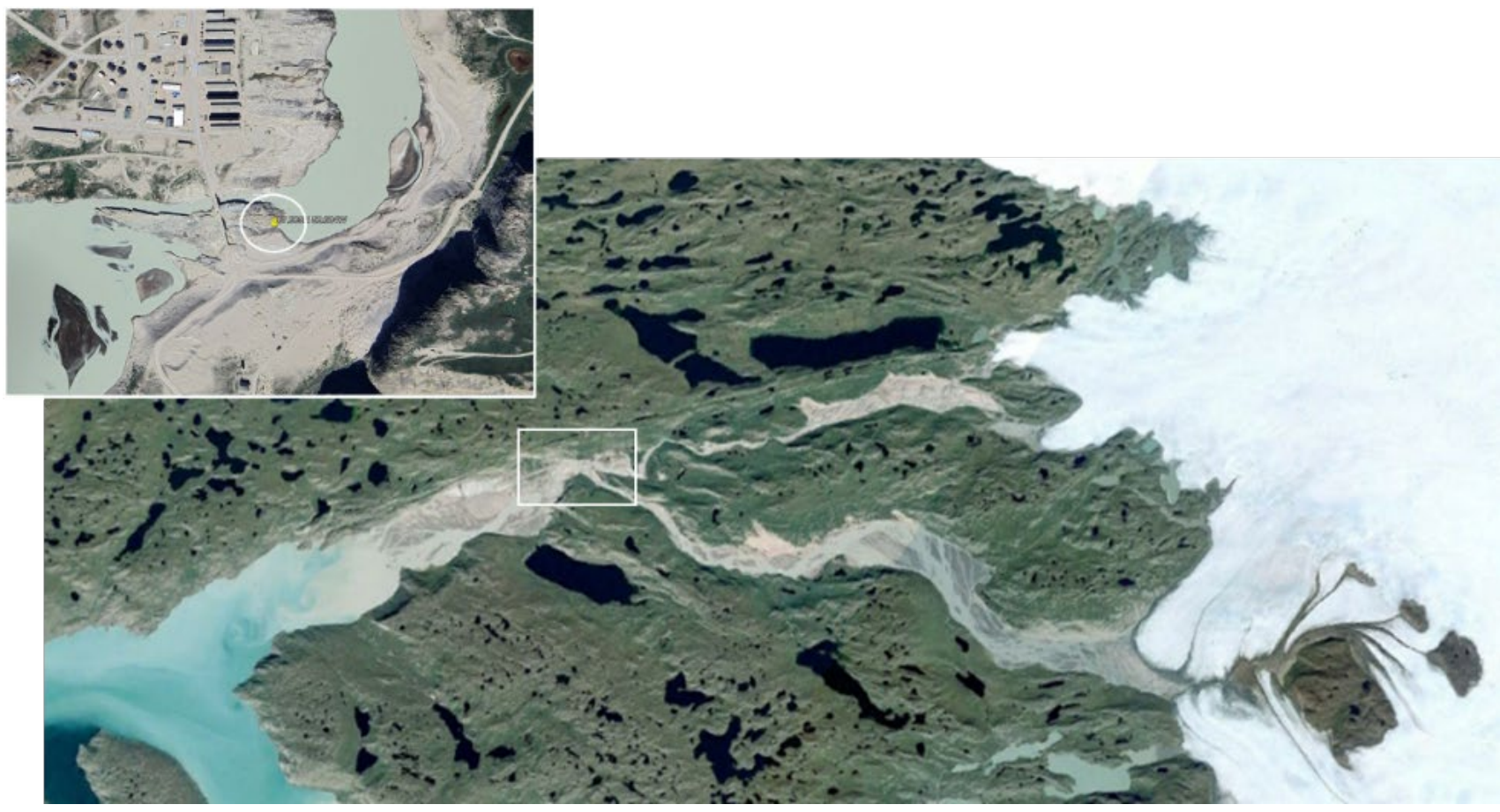

Picture from Google Earth showing location of primary sampling station, which is identical to the sampling station "Ctrl 4" of [ref.12].

At its core, the MS can be regarded as a data report providing additional results of "filtered dissolved mercury (fTHG)" and "monomethylmercury (MMHg)" at sampling stations more or less identical to [ref.11 and 12] supplemented by measurements of Hg stable isotopes, a few atmospheric Hg concentrations samples and water samples from 4 stations along Søndre Strømfjord. Based on this, the MS then seeks to explain the seasonal trend in Hg export to the ocean by invoking various sources of Hg with an assumed sensitivity to climate warming (i.e. terrestrial sources/ runoff from active layer permafrost, geogenic weathering below the GrIS, supraglacial sources/ atmospheric deposition).

2 - General assessment and comments

* Are these claims novel, and will they be of interest to others in the community? If the conclusions are not original, it would be helpful if you could provide relevant references.

The main claims of the MS are not particularly novel as time series of dissolved and particulate Hg in glacial meltwater from the same specific watershed have previously been presented and subsequently debated in scientific literature [ref.11,12]. Highly conflicting Hg concentrations were reported from the area in these two studies, where [ref.11] claims that Hg concentrations in meltwater from the GrIS is extremely elevated, which is disputed in [ref.12] in which low Hg concentrations in a similar range to the current study was reported.

As previously discussed [ref.11, ref.12], an extremely elevated Hg yield from the GrIS could have far reaching consequences for both indigenous communities and the environment in and around Greenland. The primary scientific knowledge gap needing resolution would appear to be the independent scientific verification of Hg levels in glacial meltwater from the GrIS to either support or reject the anomalously high Hg yields of [ref.11], in which several of the co-authors of the current MS where directly involved in.

In case the new data should support a rejection of the conclusions of the first controversial study (as turned out to be the case based on the 2023 data), the appropriate focus of a follow-up paper from the author group of [ref.11] would be an in-depth discussion of these significant discrepancies, with a clear declaration on the validity of the initial predictions [ref.11] which were based on a Hg sampling strategy different to the current.

The importance and prevalence of this important scientific knowledge gap is illustrated by a recent study (note: non-peer reviewed preprint) testing the previously asserted high Hg yields from the GrIS in the Nuuk fjord (Thyrring et al, 2026). The main conclusion of this study is as follows:

"*Our results, together with other studies on Hg in Greenland's waters, sediments, and planktonic species, cannot reproduce the previously reported mercury contamination from glaciers, and* ***there is now substantial evidence against the claim that Greenland ice sheet has implications for Hg budgets and coastal ecosystems***."

Ref: Thyrring, J., Krause-Jensen, D., Olesen, B. & Bjerregaard, P. (2026) No mercury from Greenland glacial runoff in sessile coastal biota | Research Square https://doi.org/10.21203/rs.3.rs-8804831/v1.

However, the MS only very superficially discusses this knowledge gap (see elaboration below). While the sampling strategy and analytical methods of the MS appear to be appropriate and credible for the 2023 campaign, the MS fails to communicate and discuss the implications of its

recent findings speaking clearly against the extreme concentration claims which parts of the author group have previously asserted to a broader audience in the scientific community.

For this reason, I cannot recommend the publication of the results in its current format and recommend a significant revision prior to potential resubmission (see suggestions below).

* On a more subjective note, do you feel that the paper will influence thinking in the field in terms of either conceptual understanding or technological capability? Please feel free to raise any further questions and concerns about the paper.

To me it seems unthinkable that the author group of the current MS did not plan the sampling strategy of the 2023 field work, with the above-mentioned specific knowledge gap in mind. And due to the controversy following the publication of [ref.11], it would be scientifically appropriate for the author team to examine whether the new data corroborate or challenge the conclusions of that earlier study, which has been substantially disputed.

When used appropriately, the data series of the MS could significantly contribute to the consolidation of the most likely concentration range in dissolved Hg in glacial meltwater from the GrIS, provide additional indices on potential sources of Hg at and around the GrIS as well as provide an example of scientific adjustment and retraction of previous conclusions and assertions as new indisputable evidence is made available.

It is clear from the MS, that the concentration levels of dissolved Hg during part of the melting season of 2023 (at the same sampling location as CTRL 4 in [ref.12]) indisputably confirms the concentration ranges reported in Jørgensen et al. 2024 [ref.12], and thereby clearly contradicts the results, conclusions and associated implications as reported in Hawkings et al, 2021 [ref.11]. A logical consequence of the current confirmation of the conclusion of [ref.12] would appear to be an in-depth discussion on the potential causes for the differences between the results of [ref.11] and the current study, including self-critical reflection on applied sampling approaches, methods etc.

In contrast, the MS includes an undebated use of [ref.11] to both frame the overall justification of the study (i.e. large differences in estimates of Hg yields from the GrIS) and implications of the study (discrepancies are a product of *difficulties in making accurate assessment*; please see above). Consequently, the current format of the MS makes the paper stand out as an indirect and non-convincing attempt to inform the scientific community that evidence supporting the conclusions of [ref.11] can no longer be produced without having to go into further details or discussions on why this could be the case and what the implications are.

In the current MS, the attempt to upscale Hg yields to the entire western margin of the GrIS (similar to [ref.11]) appears unconvincing for several reasons. One is that the upscaling exercise is based on data over a limited time period within a single calendar year (1 June to 21 July 2023) in which anomalous precipitation events were recorded. Another is that the data series going into the upscaling is based of a single sampling station at the very farthest location from the ice sheet itself (as compared to Ref. 11 and Ref.12) thereby having the largest potential input of non-GrIS derived Hg. The resulting Hg yield estimate stands out as a pure mathematical exercise with no auxiliary data supporting its validity, and thus very limited real-world relevance with an completely unconstrained uncertainty.

* Is the work convincing, and if not, what further evidence would be required to strengthen the conclusions?

Overall, the sampling procedures and chemical analysis appear OK. Presented results on dissolved Hg for 2023 is of a similar magnitude as for 2021 and 2022 (see [ref.12]) and more than one order of magnitude lower than [ref.11].

As mentioned above, an important issue for other researchers in the community relates to obtaining an independent verification of the likely order of magnitude in Hg concentrations from the GrIS as the levels from [ref.11] (i.e.100-1000 times higher than what is known for freshwater systems in the arctic) and [ref.12] (i.e. similar range of dissolved Hg as presented in the current MS) cannot be easily reconciled. In the MS, this non-reconcilable issue is only vaguely touched upon, with no further discussion on the potential implications for the validity of the assertions and conclusions of [ref.11]:

Lines 46-48: "*However, the contribution of the GrIS to the Arctic Hg cycle remains highly uncertain due to large differences in estimated Hg yields (1,440–2,310 mmol km $^{-2}$ year $^{-1}$ versus 2.7-5.2 mmol km $^{-2}$ year $^{-1}$)11,12*."

Lines 485-490: "*We estimate a southwestern GrIS total Hg yield of 23 mmol km-2 yr-1 (Table 1).* ***This yield is more than an order of magnitude lower than that reported by Hawkings et al.,*** *(2021), but comparable to data published from six major Arctic rivers (Table 1; 4 – 20 mmol km-2 yr-1)90, suggesting glaciers are a* ***non-trivial Hg source*** *to Arctic coastal waters. Discrepancies between studies underscore the challenges of accurately assessing Hg fluxes from dynamic glacierized catchments.*"

The fact that the concentration levels of the current MS most clearly supports the conclusions of [ref.12], in which the methodological deficiencies of [ref.11] was addressed, is conveyed only indirectly, with vague statements such as "...*cycle remains highly uncertain due to large differences*..." and "*Discrepancies between studies underscore the challenges of accurately assessing*...".

In my opinion, this is an unfortunate way of communicating the implications of these new results, which primary purpose should be to inform the scientific community that any signs of the previous assertions on extremely elevated Hg yields from the GrIS can no longer be detected in any of the sampling matrices investigated in the 2023 study when using appropriate and targeted sampling strategies and analysis.

3 – Other comments, recommendations and suggestions for improvement

1. I would encourage the authors to revise the MS to significantly enhance the discussion and implications of the above-mentioned main scientific knowledge gap related to the final settling of the claims of extremely elevated Hg yields from the GrIS [ref.11].
2. Aside the resolution of 1), the importance and relevance of the new data could be to provide additional site and process specific characterization of the chemical fingerprint of Hg in the Arctic environment at and around the GrIS to improve the understanding of how Hg is partitioned, potentially remobilized and/or transformed in the landscape. As presented in the 2021 AMAP Mercury Assessment, the dominating inputs of Hg to the Arctic is by long-range transport from regions outside of the Arctic. To investigate relative impact on Hg sourced from the subglacial domain, additional information on the distribution of Hg in the landscape and how this can potentially be remobilized. To achieve this, I would strongly recommend that the MS is revised with a focus on the best possible characterization and improved distinction of individual sources of Hg, for instance
    a. Hg originating in current long-range transport

b. Hg originating in historical long-range transport (e.g. remobilization of historical long-range transport from soils, sediment, ice-melt others from the terrestrial and lacustrine parts of the landscape)
c. Hg originating from current local/regional sources (e.g. subglacial geogenic Hg samples at the current location of the margin of the GrIS)
d. Hg originating from historical local/regional sources (e.g. remobilization of geogenic Hg in the sedimentary archives in proglacial outwash plains and similar)

These detailed characteristics of discrete sources could then related to and discussed in context of the mixed sampling environment in Kangerlussuq:

e. Processes description of transformations processes, how these can be tested in the different reservoirs investigated (i.e supraglacial, subglacial, terrestrial, lacustrine and marine environments in addition to the atmosphere; see SI Fig 1 and 2)
f. What's the source specific signatures of Hg in the discrete reservoirs investigated (SI Fig 1 and 2)?
g. How do results from the above match with what you find in the mixed environment – both at different locations in the freshwater environment (different distances to the ice sheet with gradually increasing inputs from soils, sediment, groundwater and lakes) as well a along Søndre Strømfjord (four or five stations ? (Fig. 1A))
h. What's the likely relative importance of subglacial geogenic Hg to other known sources of Hg to the Arctic (including long-range transported Hg).

3. The MS includes very extensive use of suggestive language (for example: *appeared to, is likely attributed, are best explained by, may relate to, etc.*) often unlinked to concrete observations. This can of course be an acceptable practice in certain cases and to some extent a matter of semantics, but excessive use of this type of argumentation structure in order to make things fit or to exaggerate the implications of results should be avoided. I recommend that the MS is revised for accuracy in this regard, particularly when describing the main claims of the paper, such as:
    a. L485: "..suggesting glaciers are a ***non-trivial Hg source*** to Arctic coastal waters"
    b. L496: "The GrIS is a ***considerable*** Hg source..."

    => For "non-trivial", "considerable" and similar wordings this to be truly meaningful, additional clarification/quantification/discussion need to be included with respect to the other known inputs of Hg to the Arctic ocean, especially the relative importance of 'naturally sourced Hg' (which is the main claim of the paper) ⇔inputs of anthropogenic Hg (which is the dominant input based on AMAP and many other studies)

4. Since a main claim of the paper is that the Hg export from the GrIS and surrounding area could potentially increase with climate warming, concrete definitions of causal effects and process specific implications by "climate warming" need to be included in the MS. It is not sufficient to simply state the generic (and here unreferenced) statement that "the Arctic is warming nearly four times the global average" without explaining how and why

this could have implications on the Hg cycle. Consider the following non-exhaustive list of examples could be the following:

a. L.23 “Early season permafrost melt” => this would likely relate to the seasonal freeze/thaw cycle of the active layer, unless the gradual expansion of the mazimum thaw depoth is the target.
b. L. 31 “Climate-driven mass loss” => what is exactly meant by this and how does it relates to process specific Hg mobilization processes tested in the MS
c. L. 40 “Climatically sensitive Hg reservoirs” => unclear and needs further explanation
d. L. 125 “ A permafrost melt stream…” => unclear and needs further explanation
e. L. 151 “Flushing of active layer and permafrost thaw…” => unclear and needs further explanation
f. L. 159 “active layer saturation in permafrost” => unclear and needs further explanation

In summary, I recommend that the MS is revised to very clearly communicate the implications of the current results in relation to what was previously proposed by parts of the co-authors group. Also, I recommend that the MS is revised to clearly define which hypothesis and scientific research questions can be tested by the data set and leave out all the suggestive explanations and vague interpretations which cannot be supported directly by the data itself. In its current form, the MS appears over-insistent on making observations from the main sampling location in Kangerlussuaq fit nicely together with the general hypothesis, i.e. that the GrIS is a significant source of Hg and thereby semi-confirming the claims of [ref.11] even though current the current concentrations are several orders of magnitude below.

Instead, a focused process study clearly discussing the strengths and limitations of the study design (taking into account the limited time series and extreme weather conditions under which the study was conducted) could potentially improve our scientific understanding on the short- and long-term sensitivity of various Hg stocks in the Arctic landscape. To achieve this, the MS would not need to include highly uncertain upscaling or claims of large scale acute global importance of its conclusions to be suitable for publication, but rather focus on what can actually be learned from the new data.

In this revision process, I recommend that improved focus is made to clearly show and discuss also the data and results from the spot sampling of lakes, sediment and glacial streams. This would include also showing co-ordinates, sampling dates and other relevant information for these samples, including analytical results of field blanks. Of interest would also be a more clear discussion on the differences in the Hg signatures between the meltwater and particles of the supraglacial domain, meltwater and particles of the subglacial domain, freshwater bodies in the proglacial landscape, run-off from the terrestrial landscape as well as in the sedimentary archive in relevant landforms in the proglacial landscape between the GrIS and the ocean.

In the revision, I recommend that the authors discuss the results and observed concentration levels of Hg in the MS against the findings of the recently published preprint by Olson et al (2026) containing a new database on mercury observations for permafrost regions. Here, published Hg data from lake sediment, vegetation, soil and water (lakes, rivers, creeks, oceans, wetlands, ponds and soil porewater) across the Arctic is summarized and presented – see example in summary table below. In comparison, the range of reported fHg/THg values of the

MS (1.6-28.5 pM corresponding to 0,3-5,7 ng/L) show similarity in magnitude of concentrations of the studied proglacial watershed and meltwater runoff from the GrIS to the database's range for the 'water category' covering a wider range of environments and locations in permafrost regions in the Arctic and subarctic.

Table 1. Summary statistics of Hg observations in permafrost-dominated regions including lake sediment ($ng\ g^{-1}$), soil ($ng\ g^{-1}$), vegetation ($ng\ g^{-1}$), and water ($ng\ L^{-1}$). The minimum, 25th percentile, median, 75th percentile, maximum, geometric mean, standard deviation, and count are included for each medium type.

	Lake Sediment ($ng\ g^{-1}$)	Soil ($ng\ g^{-1}$)	Vegetation ($ng\ g^{-1}$)	Water ($ng\ L^{-1}$)
Minimum	1	1	1	0.0
25th Percentile	45	32	9	1.5
Median	70	50	15	2.5
75th Percentile	116	90	33	5.9
Maximum	9820	503	400	32.6
Geometric Mean	71	48	17	2.0
Standard Deviation	118	48	51	5.0
Count	113613	2923	590	676

Christine Olson, Kevin Schaefer, Alyssa Azaroff, HÃ©lÃ¨ne Angot, Tom Douglas, Maria Florencia Fahnestock, Charlotte Haugk, Gustaf Hugelius, Erfan Jahangir, Sofi Jonsson, Adam Kirkwood, Jennifer Korosi, Mina Nasr, David Olefeldt, Connor Olson, Laura Sereni, Sarah Shakil, Kyra St. Pierre, Lauren Thompson, and Scott Zolkos (2026) A Consolidated Database of Mercury Observations for Permafrost Regions. Earth System Science Data. https://doi.org/10.5194/essd-2025-640
