# Supplementary material for: Mercury mobilization and export from the Greenland Ice Sheet using an ice-to-ocean approach
Source: Commun Earth Environ. 2026 Jul 16;7(1):800. doi: 10.1038/s43247-026-03818-z (PMC13626945; doi:10.1038/s43247-026-03818-z)
Supplement: Supplementary file 2 [file 43247_2026_3818_MOESM2_ESM.pdf]

Supplementary Information for

Mercury mobilization and export from the Greenland Ice Sheet using an ice-to-ocean approach

Authors

Amina N Youssef[1], Sarah E Janssen[2], Carl Lamborg[3], Michael T Tate[2], Marissa Despins[3], Eva L Doting[1,4], Brett A Poulin[5], David McCabe[1], Leah M Hopf[1], Emma M Kocik[1], Abe Doroshow[1], Guillaume Lamarche-Gagnon[6], Ian Delaney[7], Marjolein Gevers[7], Jade Hatton[8,9], Iris Kubler-Dudgeon[10], Jack G Murphy[1], Amina T Schartup[10], Marek Stibal[9], Johannes West[10], and Jon R Hawkings[1,6]

[1]University of Pennsylvania, [2]U.S. Geological Survey, [3]University of California, Santa Cruz, [4]Norwegian Institute for Nature Research (NINA), Tromsø, Norway, [5]University of California, Davis, [6]iC3, UiT – The Arctic University of Norway, [7]University of Lausanne, Switzerland, [8]UK Centre for Ecology & Hydrology, [9]Charles University, Prague, [10]Scripps Institution of Oceanography – University of California San Diego

This Supplementary Information contains:

Supplementary Texts 1–6 and Supplementary Figures 1–3.

Supplementary Text 1 details the hydrological monitoring that occurred across the field season, including discharge measurements, precipitation measurements, and water quality measurements (pH, dissolved oxygen concentration, specific conductivity, turbidity, and water temperature). Supplementary Text 2 details sample collection, processing, and storage for supporting geochemical analytes collected across the 2023 melt season. Supplementary Text 3 describes the laboratory sample analysis and subsequent processing on the supporting geochemical analytes. Supplementary Text 4 details sample collection, analysis, and results for total gaseous mercury (TGM) concentrations and atmospheric mercury (Hg) stable isotopes. Supplementary Text 5 details the analysis and results for suspended sediment four-stage sequential extractions to characterize the lability and phase distribution of particulate total Hg (pTHg) in the Qinnguata Kuussua. Supplementary Text 6 describes data analysis for the principal component analysis

(PCA). Supplementary Fig. 1 provides Hg synoptic sample locations and concentrations for filtered total Hg (fTHg) and pTHg. Supplementary Fig. 2 displays a map of atmospheric Hg concentrations from Tekran low-level Hg air samplers (*Mer*PAS) across the ice-to-ocean gradient. Supplementary Fig. 3 shows the PCA for the time series samples.

Supplementary Text 1 | Hydrological monitoring

An AquaTroll 600 multiparameter sonde was used to measure pH, dissolved oxygen concentration and saturation, specific conductivity (SPC), turbidity, and water temperature at each sampling location. The pH sensor was calibrated every three days and SPC and dissolved oxygen concentration were calibrated before use. An additional conductivity-depth-temperature sensor and turbidity sensor were temporarily installed near the daily sampling site for measurements every 10 to 15 minutes during the sampling period.

Suspended sediment concentration (SSC) was calculated by filtering a known volume of water (~300 mL) through a pre-weighed cellulose acetate 47 mm 0.45 µm membrane. The membranes were dried overnight at 50 °C and reweighed. High-resolution records of SSC were derived by calibrating turbidity measurements against individual SSC measurements (Table S6).

Supplementary Text 2 | Sample collection, processing and storage for supporting geochemical analytes

Hg synoptic samples

Synoptic samples were taken around the Kangerlussuaq catchment to assess the composition of potential Hg inputs into Qinnguata Kuussua. Sampling locations included meltwater rivers (Ørkendalen, Russell Glacier, and Leverett Glacier) upstream of the daily sampling location, lake samples, active layer permafrost melt streams, and supraglacial water samples close to the ice margin (Table S2). These samples were collected identically to the daily samples, with associated multiparameter sonde measurements and other chemical parameters measured. Concentrations for each sample are shown in Supplementary Fig. 1.

Supporting geochemical analytes

A suite of biogeochemical analytes were collected, including dissolved organic carbon (DOC), total nitrogen (TN), major ions (Ca^{2+}, Mg^{2+}, K^{+}, Na^{+}, Cl^{-}, F^{-}, SO_4^{2-}), macronutrients (nitrite+nitrate (NO_2^{-}+NO_3^{-}), ammonia (NH_3), soluble reactive phosphorus (SRP), dissolved silicon (DSi), and particulate organic carbon (POC) (Table S1). All analytes bar POC were filtered using the same techniques as described for Hg in the main text. Samples for DOC and TN were collected in muffled (550 °C, 5 hrs) 60 mL amber glass vials and were stored frozen

until analysis. Samples for macronutrients were collected in acid-washed (hydrochloric acid [HCl], 24 hrs) 50 mL centrifuge tubes and stored frozen until analysis. Major ion and DSi samples were collected in 15 mL centrifuge tubes and stored refrigerated until analysis.

Field blanks

A series of field blanks (filtered and unfiltered) were collected during the sampling period using ultrapure water (UPW; 18.2 MΩ.cm) and methodologies as described to determine any procedural contamination for each analyte (Table S9). Briefly, three filtered Hg blanks were collected using the peristaltic pump with acid-cleaned platinum-cured silicone tubing and a 0.45 µm capsule filter (Geotech dispos-a-filter™) with a PES (polyethylsulfone) membrane. Three unfiltered Hg blanks were collected using only the peristaltic pump and tubing per field sample collection.

Supplementary Text 3 | Sample analysis for supporting geochemical analytes

DOC, POC, and TN

Prior to analysis, samples were defrosted, acidified to pH 2, and measured on an EnviroTOC analyzer (Elementar, Germany). Samples were sparged with ultrapure air for 10 minutes before analysis by high-temperature (850 °C) catalytic oxidation with infrared (DOC as CO_2) and electrochemical detection (TN as N_2). For each sample, the mean of five replicate injections of 0.5 mL was reported. DOC concentrations were calculated from a calibration curve of standards ranging from 0.05–5 mg C L^{-1}, gravimetrically prepared from a certified potassium hydrogen phthalate standard (1000 ± 10 mg C L^{-1}, Spectrum Chemical). TN concentrations were calculated from a manual calibration curve based on standards ranging from 0.005 – 0.5 mg N L^{-1}, gravimetrically prepared from a certified nitrate nitrogen standard and an ammonium nitrogen standard (both 1000 ± 4 mg L^{-1}, Sigma Aldrich). The limit of quantification (LOQ) was 0.06 mg L^{-1} for DOC and 0.006 mg L^{-1} for TN (where LOQ = limit of blank + 5 x standard deviation of a low concentration sample, Armbruster et al 2008). A standard containing 0.25 mg L^{-1} DOC and 0.025 mg L^{-1} TN was injected every 10 samples throughout the run, yielding an analytical precision of ± 0.02 mg L^{-1} DOC and ± 0.002 mg L^{-1} TN.

POC was measured at the UC Davis Stable isotope facility by Elemental Analyzer-Isotope Ratio Mass Spectrometry (EA-IRMS). Sediments were combusted at 1080 °C with oxygen dosing to ensure complete combustion. Following combustion, residual oxygen and nitrogen oxides were removed by passing the combustion products over reduced copper at 650 °C. Water was trapped by magnesium perchlorate and phosphorous pentoxide. CO_2 was separated by a GC column in the Sercon EAs and by an adsorption trap in the Elementar EAs. After separation, an aliquot of the analyte gas was carried to the IRMS for measurement. Instruments were calibrated between 200-2000 μg C.

Major ions

Fluoride (F^-), chloride (Cl^-), and sulfate (SO_4^{2-}) were quantified using a Thermo Scientific Dionex Inuvion RFIC system. The system was equipped with an IonPac AS11-HC-4 μm 2 x 250mm analytical column and suppressed conductivity detection using 30 mM potassium hydroxide eluent at 0.38 mL min^{-1}. Data processing was performed with Thermo Scientific Chromeleon software.

Macronutrients

Nitrate (NO_3^-) + Nitrite (NO_2^-), ammonium (NH_4^+), soluble reactive phosphorus (SRP), and dissolved silicon (DSi) were quantified using a FIALab FLEX flow injection analyzer according to FIALab methods PO4-W-20-6, NH3-W-20-4, NO3-W-20-1, and SIO2-W-20-1. Data processing was performed in Microsoft Excel on raw data exported from FIAsoft software.

Supplementary Text 4 | Atmospheric Hg sample collection, analysis, and results

Atmospheric Hg sample collection

Four sets of Hg air samplers (*Mer*PAS Low-Level Outdoor Sampler)[1] were deployed 2 m above ground along an ice-to-ocean transect for the duration of the sampling period (~50 days). *Mer*PAS samplers were used to assess the potential deposition/emission of TGM across the season. Samplers were deployed in triplicate from the head of Kangerlussuaq Fjord to Leverett glacier (Supplementary Fig. 2 and Table S4).

An active air sampler designed to collect TGM was deployed at one of the *Mer*PAS sites (67.055°N, 50.430°W; Sanders Moraine, Supplementary Fig. 2) to collect atmospheric Hg stable isotope samples[2]. Air was pumped through a 0.45 µm polytetrafluorethylene (PTFE) filter and a soda lime trap to trap particles and water vapor, with TGM collected on two chlorinated activated carbon traps connected in series. The sample flow rate was 1 L min^{-1} and activated carbon traps were replaced every two weeks for a total of 3 active Hg air samples across the field season (Fig. 6, main text, and Table S4).

Atmospheric Hg analysis

TGM concentrations from the *MerPAS* air samplers were determined using thermal desorption, amalgamation, and atomic absorption spectroscopy on a Milestone DMA-80[1,3]. Hg air concentration (ng m^{-3}) was calculated by dividing the Hg concentration (ng) by the known air sampled volume for each sampler deployed in the field (sampler rate 0.111 m^3 day^{-1}).

TGM concentrations from the active air sampler were quantified by a dual combustion furnace and collection into a 3:1, nitric acid [HNO_3]:HCl trap. The trapping solution was analyzed for TGM concentration using EPA 1631 via cold vapor atomic fluorescence spectrometry (CVAFS)[4]. Hg stable isotopes were assessed by introducing the oxidant trap to the multi-collector inductively coupled plasma mass spectrometer (MC-ICP-MS) using stannous chloride reduction coupled to a custom-designed gas liquid separator. Thallium (40 ng mL^{-1}) was used for mass bias correction and was simultaneously introduced into the gas-liquid separator as an aerosol using an Apex-Q desolvating nebulizer. Hg isotopes were analyzed following standard-sample bracketing protocol[5] using National Institute of Standards and Technology (NIST) 3133.

Atmospheric Hg in the Kangerlussuaq catchment is a minor Hg source

Total gaseous mercury (TGM) concentrations ranged from 1.2–1.3 ng m^{-3} (Supplementary Fig. 2), consistent with previous values reported for Arctic and sub-Arctic environments and comparable to levels measured across the United States[6–9]. Concentrations are also consistent with long-term observations in Greenland[10] (~1.3 ± 0.4 ng m^{-3}). TGM change across the observation period is minimal. TGM may therefore be a consistent, but minor Hg source to the Qinnguata Kuussua, and dry deposition, attributed by Δ^{200}Hg and Δ^{204}Hg (Fig. 6c, main text) is

likely the main pathway for atmospheric-land cycling of Hg in this region. All TGM samples had minimal MDF for δ^{202}Hg and minimal odd-MIF fractionations for Δ^{199}Hg and Δ^{201}Hg and were therefore not similar in composition to fTHg or pTHg samples.

Supplementary Text 5 | Hg sediment extractions

Hg sequential sediment extraction analysis

Suspended sediment samples collected on PES membrane filters were subjected to a four-stage sequential extractions (Table S1) following an established method[11]. Sequential extractions began with a pH 2 soluble fraction (denoted as F2)[11], where filters were treated with 10 mL of pH 2 (0.1 M acetic acid [CH_3COOH] + 0.01 M HCl) and agitated on a shaker table at 30 rpm for 22 hours. The supernatant was then decanted into a cleaned 40 mL vial, the sediment pellet was rinsed with an additional 10 mL of solution, and the rinse solution was decanted into the same vial. The organic and chelated fraction (F3) was extracted by treating the residual sediment with 10 mL of 1.0 M potassium hydroxide (KOH) solution using the same agitation and centrifugation parameters as F2. Subsequent fractions included F4 (12 M HNO_3) and F5 (aqua regia, prepared in situ using 10 mL concentrated HCl and 3.0 mL concentrated HNO_3). All extracts were diluted with ultra-pure water to 30 mL and oxidized with bromine monochloride (BrCl; 1% v/v for F2, F4, and F5; 8% v/v for F3) prior to analysis. Samples were analyzed on a Tekran 2600 (CVAFS)[4]. Field procedural blanks and reagent blanks (e.g. no filter) were all ≤0.20 ng L^{-1} (≤1.0 pM, Table S10). Ongoing precision and recovery (OPR) spikes of 1.0 ng L^{-1} Hg (~5 pM) and 5.0 ng L^{-1} Hg (~20 pM) were tested every 5 samples, with precisions of ± 9.9 % and ± 10.4 % (RSD) respectively. For data interpretation F4 and F5 (12 M HNO_3 and aqua regia) were combined (Fig. 2d, main text), as Hg concentrations in the F4 fraction were at or below detection limits (assessed as 3 s.d. of the reagent blank; <0.20 ng L^{-1} or <1.0 pM) for nearly all samples.

Sequential extractions suggest pTHg is of subglacial origin

A qualitative assessment of pTHg lability utilizing sequential particulate Hg extractions demonstrated that Hg was predominantly associated with the KOH-extractable phase (87.3-96.7 % of the total extracted Hg; Fig. 2d, main text). Given that the organic content of this material was low (seasonal average POC % weight was 0.23 ± 0.11 % , n = 47), we tentatively interpret

the predominance of KOH-extractable Hg as Hg adsorption driven by trace organic matter on suspended particulates, Hg adsorbed to iron oxyhydroxides (found abundant in glacial meltwater)[12], or Hg(II) adsorbed to reactive clay-substrates with a negative charge[13]. The latter mechanism is particularly important in relatively low Hg concentration samples, where soluble Hg absorbs onto mineral surfaces[11]. Microparticles and secondary weathering products are common in subglacial sediments, and we postulate that these particles transport recently weathered, reactive Hg(II) at the ice-bed interface and downstream[14,15].

Supplementary Text 6 | Data analysis

Principal Component Analysis (PCA) was performed using R Studio (version 2024.12.1+563)[16]. Only the daily Qinnguata Kuussua time series samples were selected for PCA. Samples containing missing values, mainly for monomethylmercury (MMHg), were estimated using dummy numbers based on simple interpolation to allow these samples to be included in the analysis. Briefly, for high-MMHg days with concentrations well above detection (e.g. early season), missing values were replaced with the mean of the preceding and following days, whereas for low-MMHg periods (e.g. late season), missing values were set to the analytical detection limit (0.10 pM; 100 fM). All variables were standardized (centered and scaled to unit variance) to ensure comparability across different measurement units. PCA was performed using the prcomp() function with scaling enabled. The first two principal components were visualized using a biplot generated with ggplot2[17] and ggrepel[18] packages. The PCA was performed to assess geochemical analyte correlations to Hg speciation (fTHg, MMHg, and pTHg) and provide context to temporal changes in Hg sources.

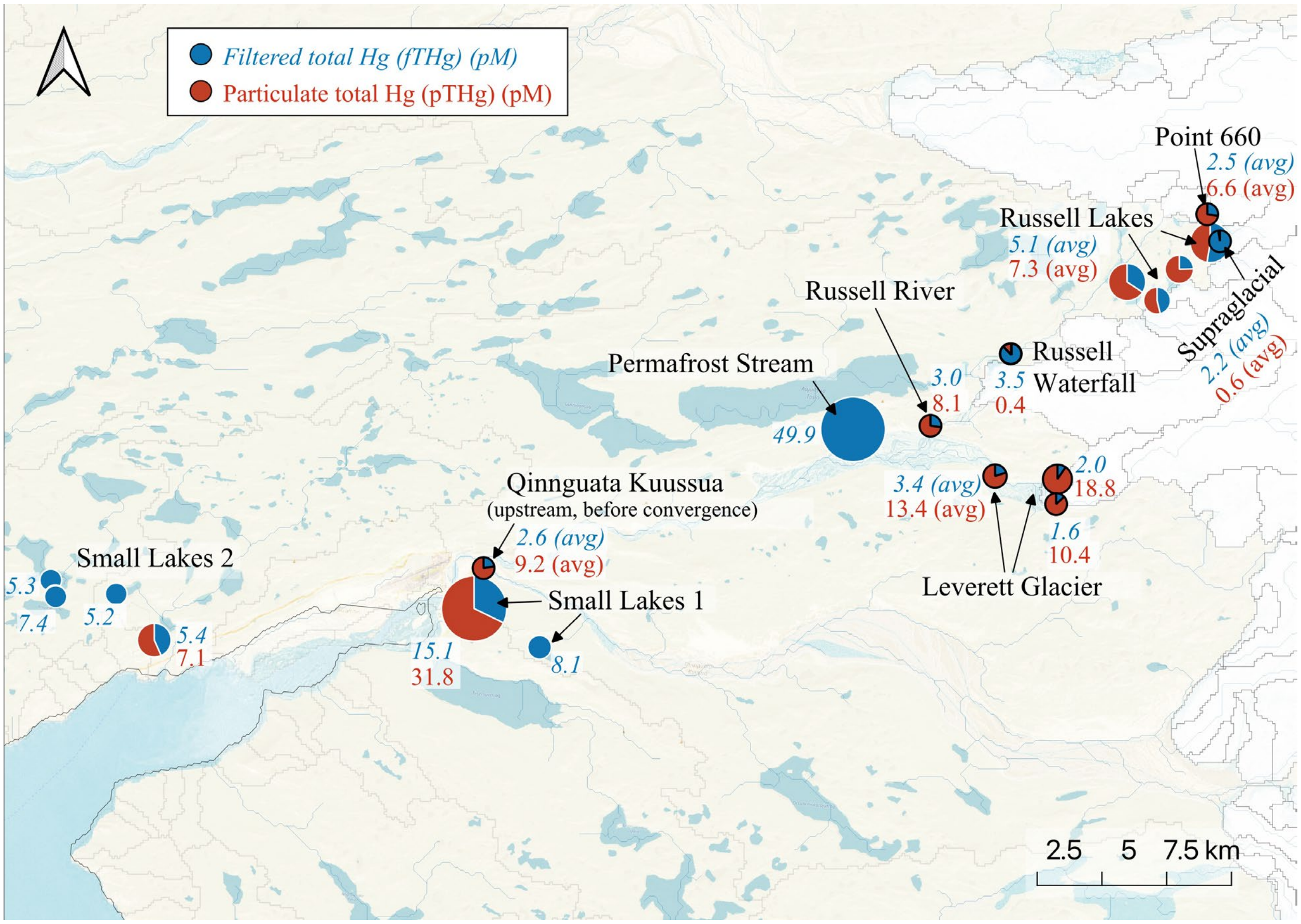

Supplementary Fig. 1. Synoptic samples collected around the Kangerlussuaq catchment for filtered total Hg (fTHg, italics, blue) and particulate total Hg (pTHg, red). The sum of fTHg and pTHg equal the total Hg concentration (uTHg). Glacier samples have a black border, non-glacial samples (i.e. lakes or permafrost streams) have a white border. Samples collected more than once are averaged (avg). The size of each point scales to uTHg (pM). The largest point is scaled to an area of 15 mm, and is equal to ~49 pM uTHg, and the smallest points are scaled at 5 mm in area and range between 2-5 pM uTHg. Grey lines on the ice sheet show glacial catchments[19]. Figure made using QGreenland[20] with basemap data from OpenStreetMap contributors[21].

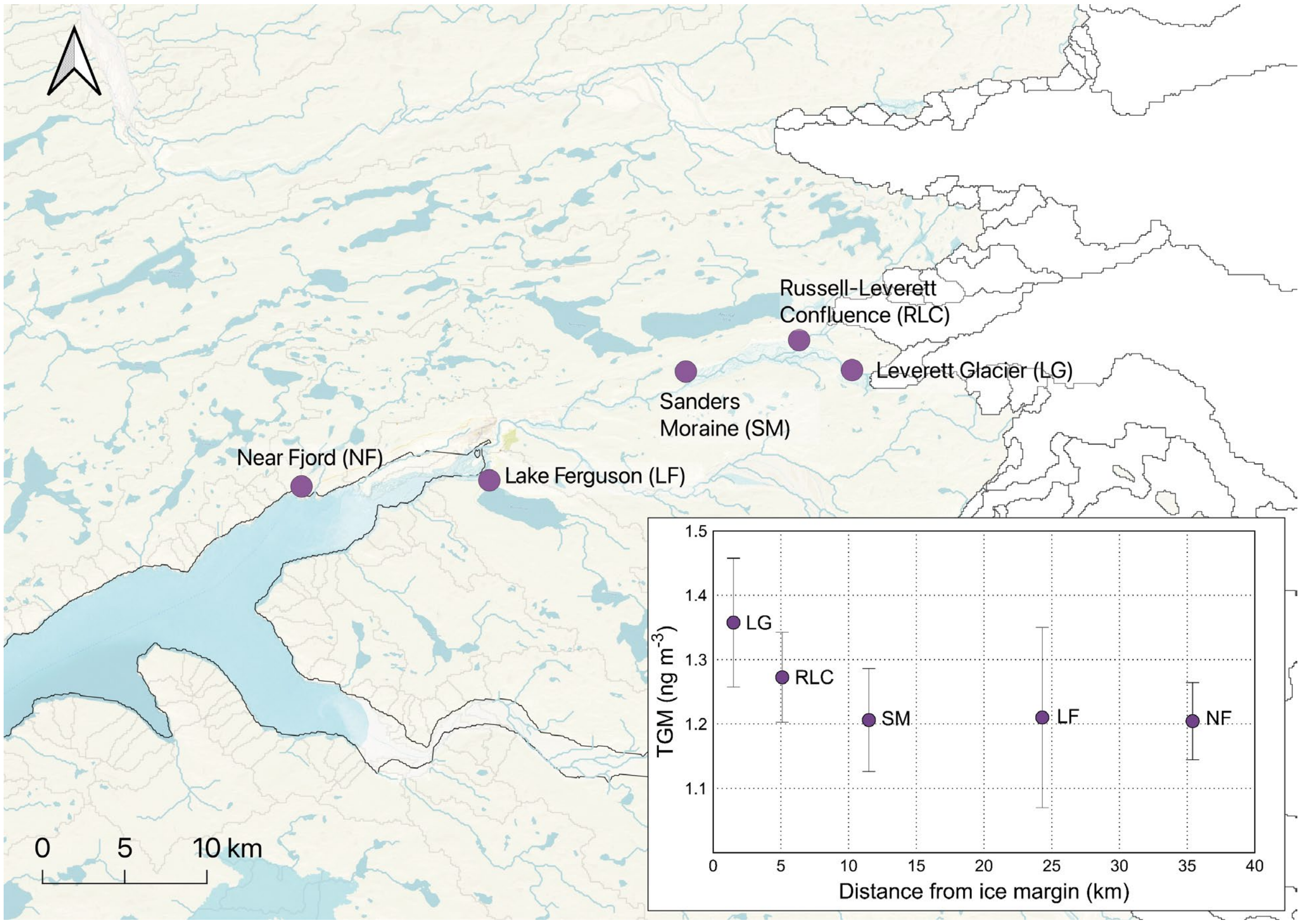

Supplementary Fig. 2. Map of atmospheric Hg concentrations from Tekran low-level Hg air samplers (*Mer*PAS). Graph (bottom right) plots average total gaseous mercury (TGM, ng m^{-3}) for each location against distance from the ice. Samplers were deployed in sets of three and an average concentration was calculated for each location. Relative error was calculated using the range in standard deviation for each set of air samplers. Grey lines on the ice sheet show glacial catchments[19]. Figure made using QGreenland[20] with basemap data from OpenStreetMap contributors[21].

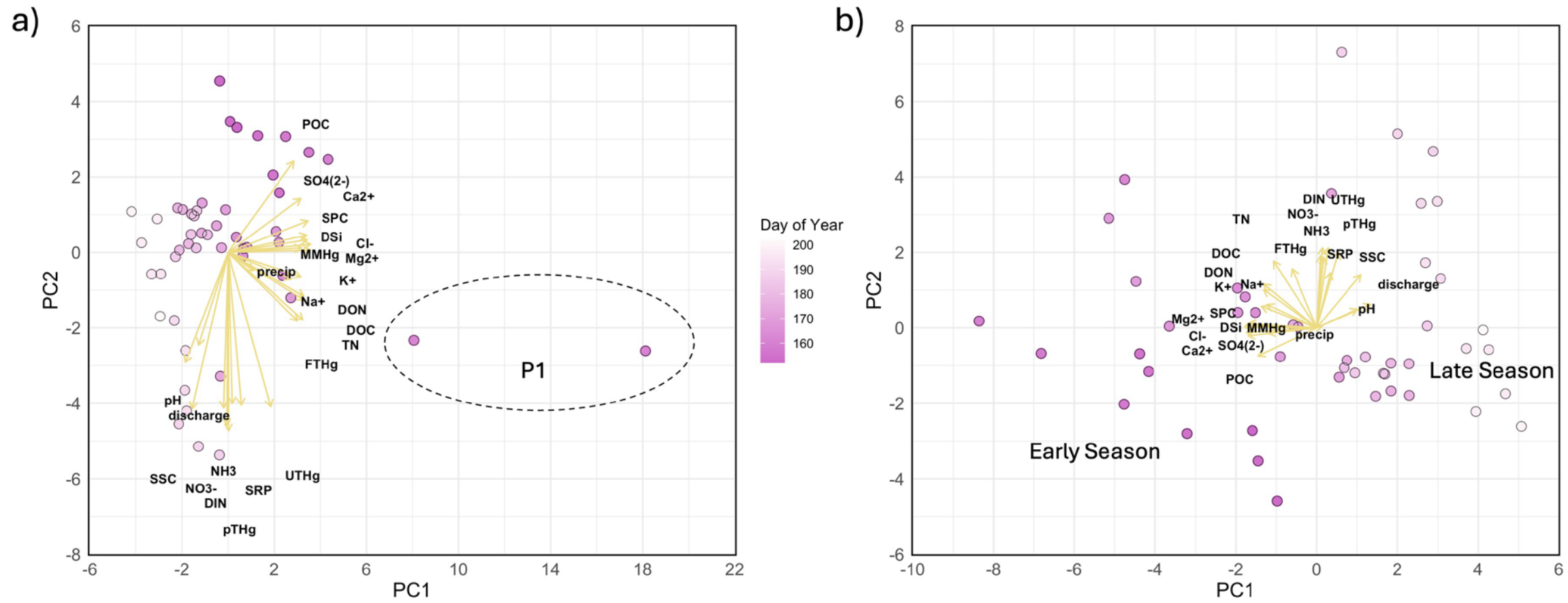

Supplementary Fig. 3. Principal Component Analysis (PCA) for time series samples. a) PCA including P1 outliers (days 161 and 162). PC1 explains 51 % of the geochemical variance and PC2 explains 21 % of the variance. Together, the biplot explains 72 % of the total geochemical variance in the dataset. b) PCA excluding P1 outliers. PC1 explains 43 % of the geochemical variance and PC2 explains 23 % of the variance; both explaining 66 % of the total geochemical variance. Samples in both plots are distinguished by day of year (e.g. pink color gradient). Variables on both plots include: precipitation (precip), discharge, suspended sediment concentration (SSC), pH, specific conductivity (SPC), soluble reactive phosphorous (SRP), dissolved silicon (DSi), dissolved organic carbon (DOC), total nitrogen (TN), ammonia (NH_3), nitrate (NO_3^-), dissolved inorganic nitrogen (DIN), particulate organic carbon (POC), filtered total mercury (fTHg), monomethylmercury (MMHg), unfiltered total mercury (uTHg), particulate total mercury (pTHg), calcium (Ca^{2+}), potassium (K^+), magnesium (Mg^{2+}), chloride (Cl^-), and sulfate (SO_4^{2-}).

Disclaimer: Any use of trade, firm, or product names is for descriptive purposes only and does not imply the endorsement by the U.S. Government.

Supplementary References

1. McLagan, D. S. *et al.* A High-Precision Passive Air Sampler for Gaseous Mercury. *Environ. Sci. Technol. Lett.* **3**, 24–29 (2016).
2. Tate, M. T., Janssen, S. E., Lepak, R. F., Flucke, L. & Krabbenhoft, D. P. National-Scale Assessment of Total Gaseous Mercury Isotopes Across the United States. *J. Geophys. Res. Atmospheres* **128**, e2022JD038276 (2023).
3. U.S. EPA. Method 7473 (SW-846): Mercury in Solids and Solutions by Thermal Decomposition, Amalgamation, and Atomic Absorption Spectrophotometry (Revision 0). in (Washington, DC, 2007).
4. US-EPA. *Method 1631, Revision E: Mercury in Water by Oxidation, Purge and Trap, and Cold Vapor Atomic Fluorescence Spectrometry*. https://www.epa.gov/sites/production/files/2015-08/documents/method_1631e_2002.pdf (2002).
5. Blum, J. D. & Bergquist, B. A. Reporting of variations in the natural isotopic composition of mercury. *Anal. Bioanal. Chem.* **388**, 353–359 (2007).
6. Berg, T. *et al.* Atmospheric mercury species in the European Arctic: Measurements and modelling. *Atmos. Environ.* **35**, 2569–2582 (2001).
7. Gratz, L. E. *et al.* First Measurements of Ambient Total Gaseous Mercury (TGM) at the EvK2CNR Pyramid Observatory in Nepal. *E3S Web Conf.* **1**, 27004 (2013).
8. McLagan, D. S. *et al.* Global evaluation and calibration of a passive air sampler for gaseous mercury. *Atmospheric Chem. Phys.* **18**, 5905–5919 (2018).

9. Quant, M. I. *et al.* Using Passive Air Samplers to Quantify Vertical Gaseous Elemental Mercury Concentration Gradients Within a Forest and Above Soil. *J. Geophys. Res. Atmospheres* **126**, e2021JD034981 (2021).
10. MacSween, K. *et al.* Updated trends for atmospheric mercury in the Arctic: 1995–2018. *Sci. Total Environ.* **837**, 155802 (2022).
11. Bloom, N. S., Preus, E., Katon, J. & Hiltner, M. Selective extractions to assess the biogeochemically relevant fractionation of inorganic mercury in sediments and soils. *Anal. Chim. Acta* **479**, 233–248 (2003).
12. Hawkings, J. R. *et al.* Ice sheets as a significant source of highly reactive nanoparticulate iron to the oceans. *Nat. Commun.* **5**, 3929 (2014).
13. Kim, C. S., Bloom, N. S., Rytuba, J. J. & Brown, G. E. Mercury Speciation by X-ray Absorption Fine Structure Spectroscopy and Sequential Chemical Extractions: A Comparison of Speciation Methods. *Environ. Sci. Technol.* **37**, 5102–5108 (2003).
14. Crompton, J. W., Flowers, G. E., Kirste, D., Hagedorn, B. & Sharp, M. J. Clay mineral precipitation and low silica in glacier meltwaters explored through reaction-path modelling. *J. Glaciol.* **61**, 1061–1078 (2015).
15. Graly, J. A., Humphrey, N. F., Landowski, C. M. & Harper, J. T. Chemical weathering under the Greenland Ice Sheet. *Geology* **42**, 551–554 (2014).
16. R Core Team. R: A language and environment for statistical computing. R Foundation for Statistical Computing (2021).
17. Wickham, H. ggplot2: Elegant Graphics for Data Analysis. (2016).
18. Slowikowski, K. ggrepel: Automatically Position Non-Overlapping Text Labels with ‘ggplot2’. (2024).

19. Mankoff, K. D. *et al.* Greenland liquid water discharge from 1958 through 2019. *Earth Syst Sci Data* **12**, 2811–2841 (2020).

20. Moon, T., Fisher, M., Stafford, T. & Harden, L. QGreenland. Zenodo https://doi.org/10.5281/ZENODO.12823307 (2023).

21. OpenStreetMap contributors. OpenStreetMap. OpenStreetMap Foundation https://openstreetmap.org (2025).
