# Supplementary material for: Mercury mobilization and export from the Greenland Ice Sheet using an ice-to-ocean approach
Source: Commun Earth Environ. 2026 Jul 16;7(1):800. doi: 10.1038/s43247-026-03818-z (PMC13626945; doi:10.1038/s43247-026-03818-z)
Supplement: Supplementary file 3 [file 43247_2026_3818_MOESM3_ESM.docx]

**Description of Additional Supplementary file**

File name: Source data file

Description: Source data for all main-text figures and Supplementary figures are provided in this accompanying Source Data file.
